# Trifunctional electrocatalyst with accurate surface reconstruction for zinc-air batteries and water electrolyzers

**DOI:** 10.1038/s41467-026-74714-5

**Published:** 2026-06-23

**Authors:** Lingjie Yuan, Wei-Hsiang Huang, Zheng Tang, Su-Yang Hsu, Yalei Fan, Beibei Wang, Mingkai Xu, Tsung-Yi Chen, Min-Hsin Yeh, Jin-Ming Chen, Zhiwei Hu, Yinlong Zhu

**Affiliations:** 1 Institute for Frontier Science, Nanjing University of Aeronautics and Astronautics, Nanjing, China; 2 National Synchrotron Radiation Research Center, Hsinchu, Taiwan; 3 Sustainable Electrochemical Energy Development Center, National Taiwan University of Science and Technology, Taipei, Taiwan; 4 Key Laboratory of Precision and Intelligent Chemistry, School of Chemistry and Materials Science, University of Science and Technology of China, Hefei, China; 5 Department of Electrophysics, National Yang Ming Chiao Tung University (NYCU), Hsinchu, Taiwan; 6 Max Planck Institute for Chemical Physics of Solids, Dresden, Germany

**Keywords:** Electrocatalysis, Electrocatalysis, Renewable energy, Electrocatalysis

## Abstract

Exploiting cost-effective trifunctional electrocatalysts toward oxygen evolution reaction, hydrogen evolution reaction and oxygen reduction reaction is important for sustainable energy conversion and storage devices yet challenging. Here, we report a single-phase trifunctional electrocatalyst Sr_2_CoRuO_6-δ_ with well-defined super-exchange double perovskite structure, which can efficiently catalyze oxygen evolution, hydrogen evolution and oxygen reduction under alkaline conditions. As an air electrode, Sr_2_CoRuO_6-δ_ delivers high peak power density of 216 mW cm^−2^, high specific capacity of 748 mAh g^−1^ and long lifespan up to 1000 h for liquid rechargeable zinc-air batteries, as well as broad temperature and deformation adaptability for solid-state flexible zinc-air batteries. Furthermore, an anion exchange membrane water electrolyzer employing Sr_2_CoRuO_6-δ_ as both cathode and anode requires a cell voltage of 1.90 V at the current density of 1 A cm^−2^ and shows a stable and rapid response when coupled with fluctuating solar electricity. Combining complementary in-situ and microscopic techniques, spatiotemporal surface reconstruction behavior of Sr_2_CoRuO_6-δ_ under varied reactions is comprehensively investigated and true active components are identified.

## Abstract (web summary)

Exploiting cost-effective electrocatalysts to drive multiple electrochemical reactions could simplify energy devices, but remains challenging. Here, the authors report a double-perovskite electrocatalyst with accurate surface reconstruction for zinc-air batteries and water electrolyzers.

## Introduction

With the ever-increasing global energy demand and growing concerns over environment pollution, sustainable energy storage and conversion technologies such as water splitting, metal-air batteries and fuel cells, have attracted significant attention^1,2^. All these technologies fundamentally rely on three key electrochemical redox reactions: oxygen evolution reaction (OER), hydrogen evolution reaction (HER) and oxygen reduction reaction (ORR)^3,4^. For instance, the anodic OER and cathodic HER serve as essential half-reactions in electrocatalytic water splitting process, and the ORR and OER dominate the discharge and charge processes in rechargeable metal-air batteries^5,6^. However, these electrochemical reactions are intrinsically sluggish and necessitate efficient electrocatalysts to lower the reaction barriers and accelerate the reaction rates. Currently, noble metal-based electrocatalysts with low onset potentials are deemed as the benchmark electrocatalysts for these reactions, such as Pt/C for the ORR/HER, and RuO_2_/IrO_2_ for the OER. However, their scarcity, high cost and inadequate electrochemical stability impede their widespread commercial applications^7,8^. Besides, in consideration of simplifying the material preparation, saving overall costs and avoiding active site interference^9,10^, it is highly desirable to develop cost-effective trifunctional electrocatalysts with high OER, HER and ORR performance to promote electrode assembly and device integration.

So far, substantial efforts have been devoted to developing trifunctional electrocatalysts, most of which are nanocomposites composed of nanocarbons and various transition metal compounds including alloys, (hydro)oxides, sulfides, selenides, phosphides and nitrides^3,4,10–14^. In these nanocomposites that show promising trifunctional performance, the single-component materials with well-defined structures generally show inferior trifunctional activity. Notwithstanding the trifunctionality in the electrocatalytic performance, the structural and compositional complexity within these nanocomposites make it difficult to accurately identify the real active components and unambiguously uncover the mechanistic origins^15–18^. Thus, exploring trifunctional electrocatalysts with well-defined structures and high overall performance is highly desirable. One promising class of functional materials with well-defined structure is metal oxides including perovskites, pyrochlores and spinels, whose catalytic performance and cost-effectiveness can be optimized by rational selection of constituent elements and coordination spheres^1,5,6,19–21^. In particular, owing to the properties of compositional/structural variety, flexible tunability, high intrinsic activity and chemical stability, perovskite oxides have emerged as advanced electrocatalysts for OER, HER and ORR^22–24^. Despite great potential in the oxygen and hydrogen electrocatalysis, most of perovskite oxides are only efficient for a single electrocatalytic reaction and advancing their trifunctional electrocatalytic performance is still in infancy with very limited explorations^25–27^. Beyond that, comprehensive integration of trifunctional catalysts into practical energy devices is still scarce, highlighting a substantial gap between fundamental research on perovskite catalysts and their practical application.

Motivated by the above considerations, we report a single-phase trifunctional double perovskite electrocatalyst Sr_2_CoRuO_6-δ_ (SCRO) with unique Co^3+^-O-Ru^5+^ super-exchange effect, which enables highly efficient electrocatalysis toward OER, HER and ORR in alkaline solution. Benefiting from its trifunctional electrocatalytic activity, SCRO was further assembled into zinc–air battery (ZAB) and anion exchange membrane water electrolyzer (AEMWE) devices. As an air electrode for ZABs, SCRO-based liquid ZAB exhibits a high peak power density of 216 mW cm^−2^, a high specific capacity of 748 mAh g^−1^, and a long stability of 1000 h, comparable to the benchmark Pt/C + RuO_2_ electrode. Also, solid-state flexible ZAB assembled with SCRO exhibits broad temperature adaptability (from 60 °C to −40 °C) and notable mechanical flexibility (from 0° to 180°). Furthermore, the assembled AEMWE system employing SCRO double perovskite as both HER and OER electrocatalysts achieves a cell performance comparable to RuO_2_ || 20% Pt/C, reaching the industrial current of 1000 mA cm^−2^ at a low voltage of 1.90 V and steadily operating for 100 h at 500 mA cm^−2^. When integrated with a commercial solar cell, the SCRO-based AEMWE device demonstrates a stable and rapid response under fluctuating output voltage. Moreover, the trifunctional SCRO catalyst enables the construction of a self-powered overall water-splitting device driven by ZABs, utilizing the same catalyst. Combining a suite of in situ/operando characterizations including in situ X-ray absorption spectroscopy (XAS) and X-ray diffraction (XRD) together with high-resolution transmission electron microscopy (HRTEM), we investigated the spatiotemporal surface reconstruction behavior of SCRO and identified the true active components under different electrochemical processes, which could be induced by the intrinsic electric field resulted from super-exchange interactions between asymmetric Co/Ru charge distribution. This work provides an important guidance for the design of multifunctional electrocatalysts for sustainable energy storage and conversion technologies.

## Results

### Synthesis and structural characterization

**Fig. 1:**
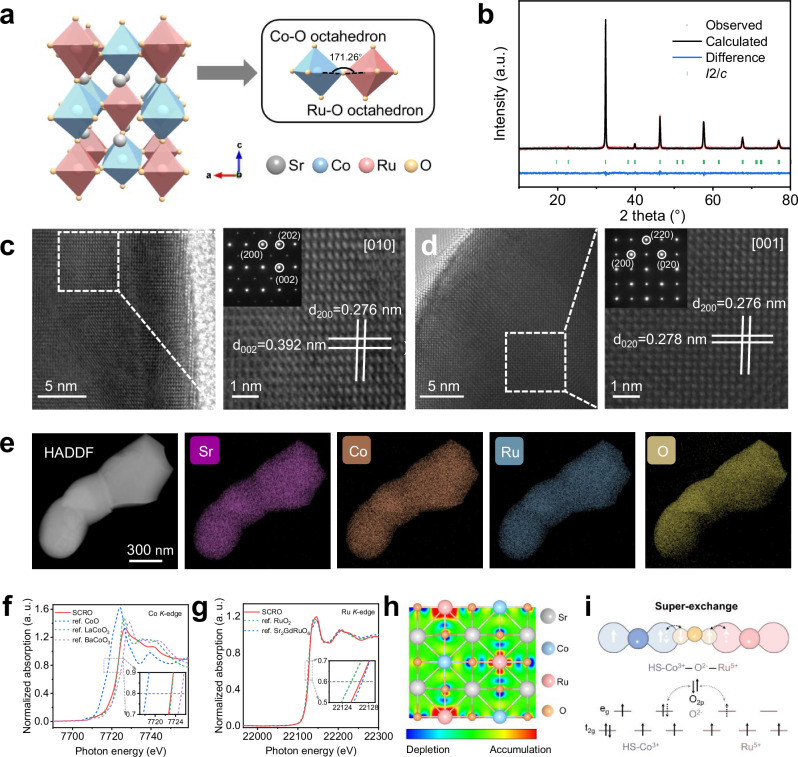
Synthesis and structural characterization. **a** Schematic representation of SCRO double perovskite structure. **b** Rietveld refined XRD pattern of SCRO. HRTEM images and the corresponding SAED patterns along **c** [010] and **d** [001] zone axes for SCRO. **e** HAADF-STEM and the corresponding elemental mappings. **f** Co *K*-edge and **g** Ru *K*-edge XANES spectra of SCRO along with several reference materials for comparison. The inset images are magnified views of the corresponding spectrum and share the same x-axis and y-axis labels and units as the main plot. **h** Charge distribution plots for SCRO. **i** Schematic illustration of super-exchange interaction in SCRO. Source data for Fig. 1 are provided as a Source data file.

Single-phase SCRO with a double perovskite structure (A_2_BB’O_6_) was synthesized via a facile solid-state reaction method. As illustrated in Fig. 1a, double perovskite SCRO features an ordered arrangement of two B-site cations, consisting of corner-sharing RuO_6_ and CoO_6_ units in a three-dimensional structure^28^. XRD and corresponding Rietveld refinement confirm that SCRO was successfully prepared comprising a pure monoclinic structure in the space group *I*2/*c* without any impurity phases (Fig. 1b and Supplementary Tables 1 and 2). The crystal structure of SCRO was further verified by two HRTEM images and the corresponding selected area electron-diffraction patterns along [010] and [001] zone axes (Fig. 1c, d). Additionally, micrometer-sized particles were observed in the scanning electron microscopy (SEM) images (Supplementary Fig. 1), demonstrating the bulk nature of SCRO synthesized by solid-state reaction method. High-angle annular dark-field scanning transmission electron microscopy combined with energy-dispersive X-ray spectroscopy elemental mapping also indicate the homogeneous distribution of Sr, Co, Ru and O elements in the as-synthesized SCRO (Fig. 1e).

To systematically study the local atomic and electronic structures of Co and Ru in SCRO, synchrotron-based XAS techniques including X-ray absorption near edge structure (XANES) and extended X-ray absorption fine structure (EXAFS) were carried out^29,30^. Figure 1f, g show the Co and Ru *K*-edge XANES curves of SCRO, as well as several reference materials with different valence states. The energy position of Co-*K* XANES spectra (at the normalized intensity of 0.8) for SCRO is similar to that of LaCoO_3_, indicating a Co^3+^ valence state in SCRO (Fig. 1f, Supplementary Fig. 2a and Supplementary Note 1)^31^. The white line of Ru-*K* XANES spectra in SCRO is very close to that of Sr_2_GdRuO_6_ reference, confirming dominated Ru^5+^ state in SCRO (Fig. 1g and Supplementary Fig. 2b)^28^. Besides, the local structural information (e.g., coordination numbers and bond lengths) of SCRO was explored by the EXAFS analysis as well^32,33^. As depicted in Supplementary Fig. 3 and Supplementary Table 3, the fitting results of EXAFS suggest that both Co and Ru ions are nearly six-coordinated with the average bond lengths of 2.09 Å for Co–O and 1.97 Å for Ru–O, respectively. The electronic structure of Co and Ru ions in SCRO were further investigated by XAS at the Co *L*_3_-edge and Ru *L*_3_-edge, whose multiple spectral features and energy position are sensitive to the valence states^34^ and spin states^35^. As shown in Supplementary Fig. 4a, both energy position and spectral feature of Co *L*_3_-edge XAS in SCRO is very similar to that of standard Sr_2_CoRuO_6_ reference, indicating high-spin Co^3+^ (HS Co^3+^) state in SCRO. The Ru *L*_3_-edge XAS of SCRO together with that of Sr_2_GdRu^5+^O_6_ as reference are shown in Supplementary Fig. 4b, which also determine the Ru^5+^ state in SCRO. Utilizing density functional theory (DFT), the Bader charge analysis of Co and Ru sites within SCRO were further conducted (Fig. 1h, Supplementary Fig. 5 and Supplementary Data 1). The calculated Bader charge of Co and Ru are +1.48 |e| and +1.96 |e| in SCRO, respectively, compared to +1.53 |e| for Co in SrCoO_3_ and +1.67 |e| for Ru in SrRuO_3_ (Supplementary Table 4 and Supplementary Note 2), implying the electron donation from Ru to Co ions in SCRO, consistent with the valence state of Co^3+^and Ru^5+^ in SCRO as discussed above. Considering the variation in the oxidation states of Co and Ru in SCRO, a charge redistribution between Co and Ru ions is inferred (Co^4+^+Ru^4+^→HS Co^3+^+Ru^5+^), which is induced by super-exchange interactions between adjacent Co and Ru sites (as schematically illustrated in Fig. 1i). On the basis of previous reports, super-exchange effect within SCRO could give rise of a short range Co^3+^-O-Ru^5+^ ferromagnetic coupling according to G-K rule, which is beneficial for better electron transport and conduction^31,36^. Besides, the formation of monoclinic phase induced by super-exchange interactions in SCRO makes the bond angle of Co-O-Ru closely approach to 180°, which could minimize the local strain and thus be favorable for electron conduction as well^31^.

### Evaluation of trifunctional electrocatalytic performance

**Fig. 2:**
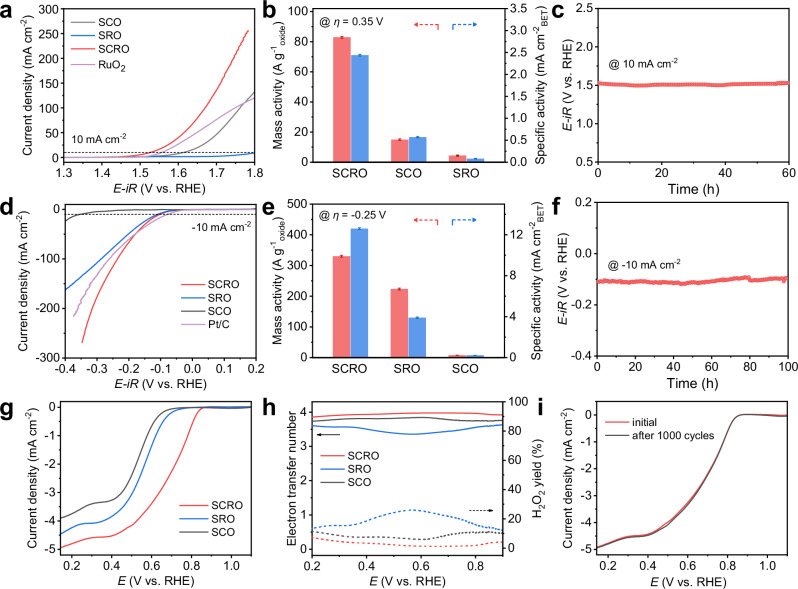
Evaluation of trifunctional electrocatalytic performance. **a** OER polarization curves with *iR*-correction of SCRO, SCO, SRO and RuO_2_ in O_2_-saturated 1.0 M KOH, where solution resistance (*R*_sol_) values were measured to be 4.75 ± 0.1 Ω for SCRO, 4.92 ± 0.1 Ω for SRO, 4.79 ± 0.1 Ω for SCO and 4.71 ± 0.1 Ω for RuO_2_. **b** Mass activities and specific activities at an overpotential of 350 mV. The error bars represent standard deviations from three independent measurements. **c** Chronopotentiometry response of SCRO at a constant OER current density of 10 mA cm^−2^. **d** HER polarization curves with *iR*-correction of SCRO, SCO, SRO and Pt/C in Ar-saturated 1.0 M KOH, where solution resistance (*R*_sol_) values were measured to be 4.64 ± 0.1 Ω for SCRO, 4.59 ± 0.1 Ω for SRO, 4.87 ± 0.1 Ω for SCO and 4.73 ± 0.1 Ω for Pt/C. **e** Mass activities and specific activities at an overpotential of −250 mV. The error bars represent standard deviations from three independent measurements. **f** Chronopotentiometry response of SCRO at a constant HER current density of −10 mA cm^−2^. **g** ORR polarization curves of SCRO, SCO and SRO on RRDE at 1600 rpm in O_2_-saturated 0.1 M KOH. **h** H_2_O_2_ yield and electron transfer number of SCRO, SCO and SRO from RRDE tests. **i** Polarization curves of SCRO initially and after 1000 CV cycles during accelerated durability tests. The ORR data are non-*iR*-corrected. Source data for Fig. 2 are provided as a Source data file.

To experimentally confirm the effect of super-exchange interactions on trifunctional electrocatalytic performance for OER/HER/ORR, SrCoO_3-δ_ (SCO) and SrRuO_3-δ_ (SRO) samples with single B-site elements were also synthesized via the same solid-state reaction method (Supplementary Fig. 6). The electrocatalytic performance of the as-prepared catalysts was evaluated in a standard three-electrode system in O_2_-saturated 1.0 M KOH for OER, Ar-saturated 1.0 M KOH for HER and O_2_-saturated 0.1 M KOH for ORR, respectively. Unless otherwise noted, all electrode potentials reported in this work were corrected with respect to reversible hydrogen electrode (RHE, see Supplementary Fig. 7 for calibration). As seen in the polarization curves obtained from linear sweep voltammetry (LSV) (Fig. 2a and Supplementary Fig. 8), SCRO necessitates a much lower OER overpotential (*η*_10_) of 297 mV to deliver 10 mA cm^−2^ compared to SCO and SRO and commercial RuO_2_, suggesting an improved alkaline OER activity. To further explore the OER kinetics, Tafel plots as required from the polarization curves were drawn. The Tafel slope for SCRO (89 mV dec^−1^) is relatively smaller than those for SCO (132 mV dec^−1^) and SRO (182 mV dec^−1^), implying faster OER kinetics of SCRO (Supplementary Fig. 9). It is commonly recognized that the apparent activity of electrocatalysts is determined by the intrinsic activity of individual active site along with the density of active sites^37,38^. To evaluate the intrinsic activity of SCRO, we calculated the specific activity by normalizing its apparent electrocatalytic activity to the real oxide surface area (ROSA) and the electrochemical surface area (ECSA). The ROSA and ECSA values of electrocatalysts were determined from Brunauer–Emmett-Teller (BET) method (Supplementary Fig. 10) and double-layer capacitance (*C*_dl_) measurements (Supplementary Fig. 11), respectively. As shown in Supplementary Figs. 12 and 13, whether the apparent activity normalized to the ECSA or ROSA, the SCRO catalyst exhibits a much higher specific activity as compared with SRO and SCO. Specifically, the SCRO catalyst exhibits a specific activity of 2.44 mA cm^−2^_BET_ at *η* = 0.35 V, nearly 4.26 and 30.5 times higher than those of SCO and SRO, respectively (Fig. 2b). Also, the mass activity of SCRO is 82.98 A g_oxide_^−1^, which is approximately 5.54 and 18.7 times higher than those of SCO (14.97 A g_oxide_^−1^) and SRO (4.43 A g_oxide_^−1^), respectively. In addition to the electrocatalytic activity, the long-term stability is also an important parameter to assess its potential for practical applications. The overpotential for SCRO displays negligible change during 60 h long-term chronopotentiometry (CP) test, indicating its robust durability of SCRO for alkaline OER (Fig. 2c).

Besides OER, electrocatalytic HER and ORR performances of SCRO were also assessed in alkaline media. For HER, the required overpotential to reach −10 mA cm^−2^ and related Tafel slopes were measured to be 101 mV and 71 mV dec^−1^, respectively, lower than those of SCO and SRO (Fig. 2d and Supplementary Figs. 14 and 15). Noticeably, the HER current of SCRO can largely exceed that of the benchmark Pt/C catalyst beyond −0.17 V. Besides, SCRO exhibits much higher specific activity and mass activity compared with SCO and SRO as well (Fig. 2e and Supplementary Figs. 16–18). In addition, the excellent electrochemical stability of SCRO during HER was also confirmed in Fig. 2f, showing only slight changes of overpotential during 100 h of continuous CP operation. With regard to the ORR, SCRO exhibits high ORR activity with onset and half-wave potentials of 0.725 V and 0.699 V, respectively, which are more positive than those of SCO and SRO (Fig. 2g). According to RRDE measurement, SCRO delivers an electron-transfer number of 3.89–4.00 over the ORR potential range (0.2–0.9 V), accompanied by a H_2_O_2_ yield below 10% (Fig. 2h), certifying that SCRO follows a highly selective four-electron transfer pathway for ORR^39^. Additionally, after 1000 CV cycles, SCRO presents an almost identical polarization curve with initial one, reflecting its superior electrochemical stability than SCO and SRO (Fig. 2i and Supplementary Fig. 19). Overall, in consideration of activity and durability, SCRO as an excellent trifunctional electrocatalyst is highly promising for practical energy device applications, as will be demonstrated below.

### Exploration of surface reconstruction behavior and true active components

**Fig. 3:**
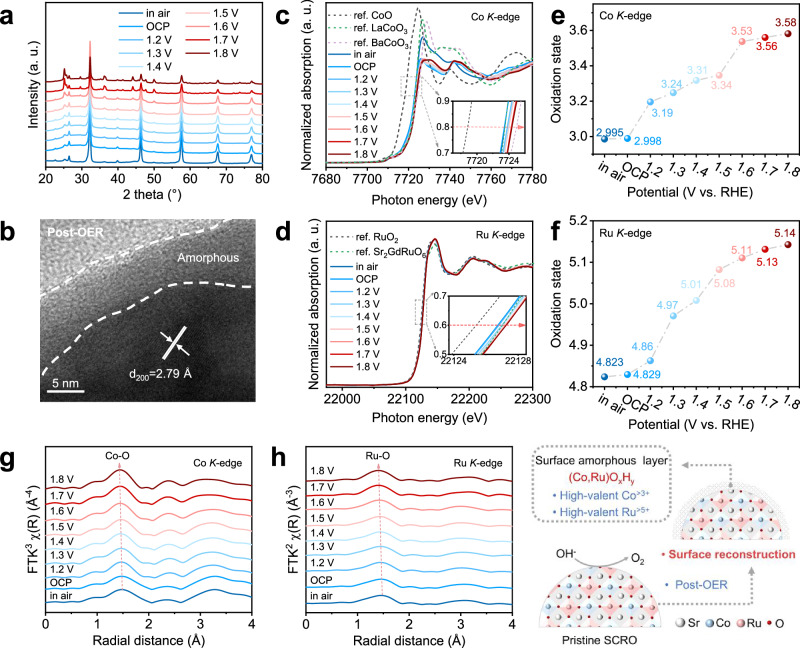
Surface reconstruction behavior and true active components for catalyzing OER. **a** In situ XRD patterns of SCRO during OER. **b** HRTEM image of post-OER SCRO. **c** Co-*K* edge and **d** Ru-*K* edge XANES spectra of SCRO at different applied OER potentials. The inset images are magnified views of the corresponding spectrum and share the same *x*-axis and *y*-axis labels and units as the main plot. Formal oxidation states of **e** Co and **f** Ru in SCRO at different OER potentials obtained from the normalized XANES spectra. In situ FT-EXAFS patterns of **g** Co-*K* edge and **h** Ru-*K* edge spectra as a function of applied OER potential. **i** Schematic diagram of the reconstructed active amorphous (Co,Ru)O_x_H_y_ surface phase in SCRO for catalyzing OER. Source data for Fig. 3 are provided as a Source data file.

It is well known that the asymmetric charge distribution commonly generates an intrinsic electric field^40,41^, which can accelerate the transfer of electrons and lower the energy barrier for the reconstruction process^42–44^. Considering the asymmetric Bader charge values of +1.48 |e| and +1.96 |e| for Co and Ru sites in SCRO (Supplementary Fig. 20), an intrinsic local electric field is expected to exist within SCRO for the propensity of reconstruction. Besides, the O 2*p* band center of SCRO is much closer to the Fermi level than SRO and SCO (Supplementary Fig. 21), implying its decreased oxide stability and improved electron transport capability, which is favorable for surface reconstruction under electrocatalytic reaction conditions^45,46^. Therefore, a series of in situ and ex situ studies were conducted to initially explore possible change of crystal/electronic structure and real active components of SCRO under OER conditions. As shown in Fig. 3a and Supplementary Fig. 22, XRD patterns recorded during in situ potential-dependent synchrotron radiation (SR)-based XRD measurements indicate a gradual attenuation of diffraction intensities without the emergence of additional peaks, which may be attributed to the formation of surface amorphous layer^32^. Consistent with the results of in situ XRD, HRTEM images obtained after the OER provide a direct reflection of reconstructed surface amorphous layer with approximately 5 nm thickness in SCRO (Fig. 3b and Supplementary Fig. 23). To gain in-depth insights into the evolutions of valence state and local coordination environment, in situ XANES at Co-*K* and Ru-*K* edges under OER potentials are presented. Here CoO, LaCoO_3_ and BaCoO_3_ with varied Co valences of Co^2+^, Co^3+^ and Co^4+^ are employed as the references. The energy positions of SCRO at air and open circuit potential (OCP) are very close to those of LaCoO_3_, indicating the Co^3+^ valence state in pristine SCRO (Fig. 3c). With the applied voltage increasing, the energy position of Co-*K* XANES spectra gradually shifts to higher energies, suggesting a transformation from Co^3+^ to Co^4+^. Following the determination of the valence transition in Co elements under OER conditions, the valence state of Ru element was also examined. Figure 3d presents the in situ Ru-*K* XANES spectra of SCRO under OER potentials, together with RuO_2_ and Sr_2_GdRuO_6_ as Ru^4+^ and Ru^5+^ references, respectively. Similar to Co ions under OER conditions, the valence state of Ru ions in SCRO continuously increases as a function of applied OER potential, indicating the transition from Ru^5+^ (at air and OCP) to unusual high-valence Ru^6+^. The accurate valence state of Co and Ru ions at different potentials were calculated by comparing the energy position with corresponding references at a jump height of 0.8/0.6 (*E*_0.8_/*E*_0.6_) (Supplementary Figs. 24 and 25)^47^. Specifically, the average Co and Ru valence states of SCRO were calculated to be +3.58 and +5.14 at 1.8 V, which are higher than those of initial SCRO (Fig. 3e, f). The local environments of active Co and Ru ions in SCRO under OER condition were further investigated by EXAFS. The Fourier transform patterns of Co-*K* and Ru-*K* edge spectra as a function of applied OER potential are shown in Fig. 3g, h. With the increased potential from OCP to 1.8 V, the Co–O and Ru–O bond lengths gradually decrease, which can be attributed to the increased valence states of Co and Ru ions^18^. As previously reported, the shortened Co–O and Ru–O bond lengths can enhance the metal-oxygen covalency, which play a significant role in boosting OER performance^48,49^. To summarize, by combining in situ XRD and XAS techniques with HRTEM experiments, the actual active components for catalyzing OER of SCRO could be identified as an amorphous (Co,Ru)O_x_H_y_ surface phase featuring high Co/Ru valence states and shortened Co/Ru–O bond lengths, as schematically illustrated in Fig. 3i.

**Fig. 4:**
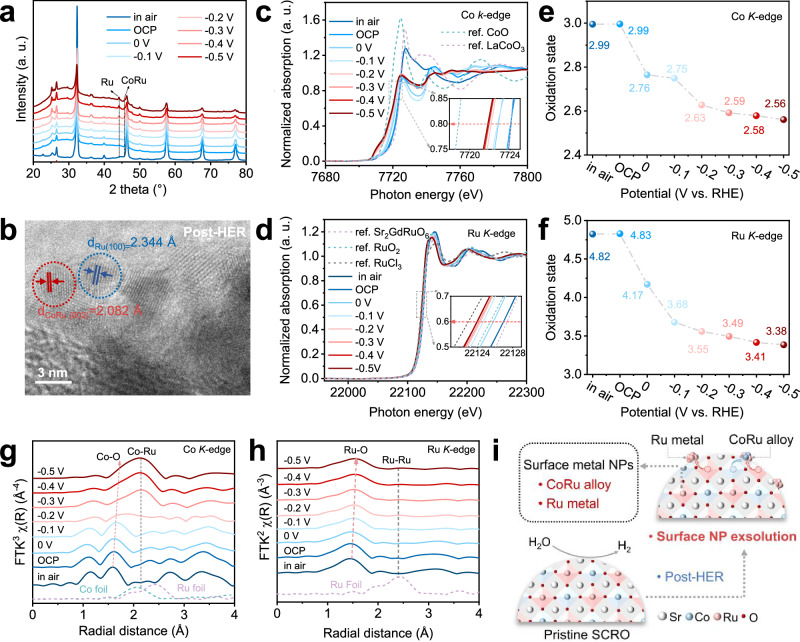
Surface reconstruction behavior and true active components for catalyzing HER. **a** In situ XRD patterns of SCRO recorded during HER. **b** HRTEM image of post-HER SCRO. **c** Co-*K* edge and **d** Ru-*K* edge XANES spectra of SCRO at different applied HER potentials. The inset images are magnified views of the corresponding spectrum and share the same *x*-axis and *y*-axis labels and units as the main plot. Formal oxidation states of **e** Co and **f** Ru in SCRO at different HER potentials obtained from the normalized XANES spectra. In situ FT-EXAFS patterns of **g** Co-*K* edge and **h** Ru-*K* edge spectra as a function of applied HER potential. **i** Schematic diagram of the exsolved active surface nanoparticles in SCRO for catalyzing HER. Source data for Fig. 4 are provided as a Source data file.

In addition to the OER, the reconstruction behavior of SCRO during HER was also investigated. Figure 4a depicts the in situ SR-based XRD patterns of SCRO under reaction potentials ranging from 0 to −0.5 V. At the potential of 0 V, an additional diffraction peak ascribing to Ru metal immediately emerges, and at a reductive potential of −0.3 V, a diffraction peak ascribing to CoRu alloy was observed as well, suggesting the reduction of Co and Ru species and the exsolution of surface metallic nanoparticles (NPs) under HER conditions^28^. Besides, on the basis of HRTEM examination, Ru metal and CoRu alloy NPs with particle size about 5 nm were directly seen on the surface of post-HER SCRO (Fig. 4b and Supplementary Fig. 26). The electronic structural evolution of SCRO under HER conditions was further studied by the in situ XANES. As shown in Fig. 4c, d, the Co *K*-edge and Ru *K*-edge XANES spectra both exhibit obvious shift toward lower energy from OCP to −0.5 V, also confirming that Co^3+^ and Ru^5+^ ions within SCRO are partially exsolved into Ru metal and CoRu alloy NPs. Compared with the standard references, the average Co and Ru valence state of SCRO at −0.5 V were calculated to be +2.56 and +3.38, respectively (Fig. 4e, f), supporting the presence of metallic Co^0^ and Ru^0^ in post-HER SCRO (Supplementary Figs. 27 and 28). Based on the changes in the average valence states relative to pristine SCRO, the apparent fractions of CoRu alloy and Ru metal were estimated to be ~14.4% and 15.6%, respectively. Further, the exsolution of surface metallic NPs can be verified by the FT-EXAFS results. In Fig. 4g, h, the peak intensities of CoRu alloy and Ru metal increase, and the Co–O-related and Ru–O-related signals simultaneously decrease with the decreased reaction potential. Also, the distance for Co–O and Ru–O bonds increase with the applied potential, which is in line with the reduced valence states. Collectively, above analysis demonstrates that the exsolved CoRu alloy and Ru metal NPs on the surface of SCRO possibly function as dual active components for alkaline HER (Fig. 4i).

**Fig. 5:**
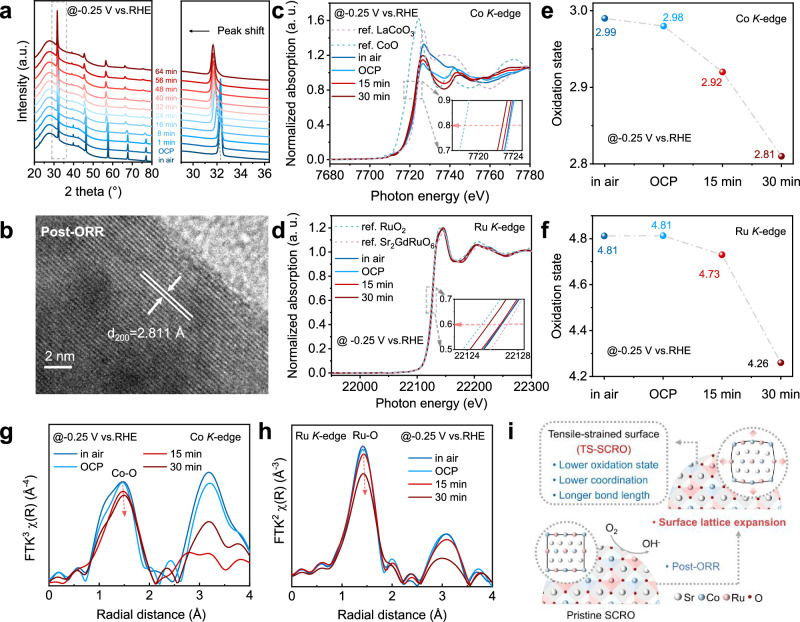
Surface reconstruction behavior and true active components for catalyzing ORR. **a** In situ XRD patterns of SCRO recorded during ORR at −0.25 V (from 1 to 64 min). **b** HRTEM image of post-ORR SCRO. In situ time-dependent **c** Co-*K* edge and **d** Ru-*K* edge XANES spectra of SCRO. The inset images are magnified views of the corresponding spectrum and share the same *x*-axis and *y*-axis labels and units as the main plot. Formal oxidation states of **e** Co and **f** Ru in SCRO at different reaction times during ORR obtained from the normalized XANES spectra. In situ time-dependent FT-EXAFS patterns of **g** Co-*K* edge and **h** Ru-*K* edge spectra. **i** Schematic diagram of tensile-strained active surface in SCRO for catalyzing ORR. Source data for Fig. 5 are provided as a Source data file.

The electronic and structural changes of SCRO during ORR were investigated as well. As shown in Fig. 5a, in situ time-dependent XRD patterns of SCRO were recorded at the potential of −0.25 V in 0.1 M KOH. During 64-min test, a clear leftward shift of the diffraction peaks was observed without additional peaks emerging, indicating the lattice expansion of SCRO under ORR conditions^50^. Also, HRTEM images obtained after ORR directly reflect the lattice expansion in post-ORR SCRO (Fig. 5b and Supplementary Fig. 29). Compared to the initial SCRO, HRTEM of post-ORR SCRO displays an expanded lattice with a *d*-spacing of 0.281 nm for (200) crystal planes, which is larger than the initial *d*-spacing of 0.276 nm. Moreover, the evolution of valence states and structural changes during ORR were investigated using in situ time-dependent XAS. From OCP to 30 min, the energy positions of Co-*K* and Ru-*K* XANES spectra in SCRO both shift to lower energies, suggesting a continuous decrease in Co/Ru valence states upon the applied reductive potential of −0.25 V (Fig. 5c, d). The specific variations in Co and Ru valence states were further calculated by comparing with standard CoO and LaCoO_3_ references (Supplementary Figs. 30 and 31). As seen from Fig. 5e, f, under ORR conditions, the average Co valence state in SCRO decreases to +2.81, and Ru valence state lowers to +4.26 after 30 min. In addition to the valence state, the possible changes regarding the local environment of Co and Ru elements in SCRO were examined by in situ EXAFS. Figure 5g, h show that the Co–O and Ru–O bond lengths both increase with reaction time, consistent with the expansion of SCRO lattice under ORR conditions. Also, the intensity of the Co–O-related and Ru–O-related peaks gradually decrease with reaction time increasing, implying a reduced coordination number of Co and Ru elements in post-ORR SCRO, in line with the trend of reduced valence state. It was previously found that the expansions of crystal lattice are conducive to the adsorptions of reaction intermediates, thereby improving catalytic ORR performance^51,52^. Consequently, the real active components for catalyzing ORR could be determined as a tensile-strained SCRO (TS-SCRO), which is induced by lattice expansion during ORR process, possessing lower Co/Ru valence states, reduced coordination numbers and longer bond lengths (Fig. 5i).

### Practical ZAB performance with multi-condition adaptability

**Fig. 6:**
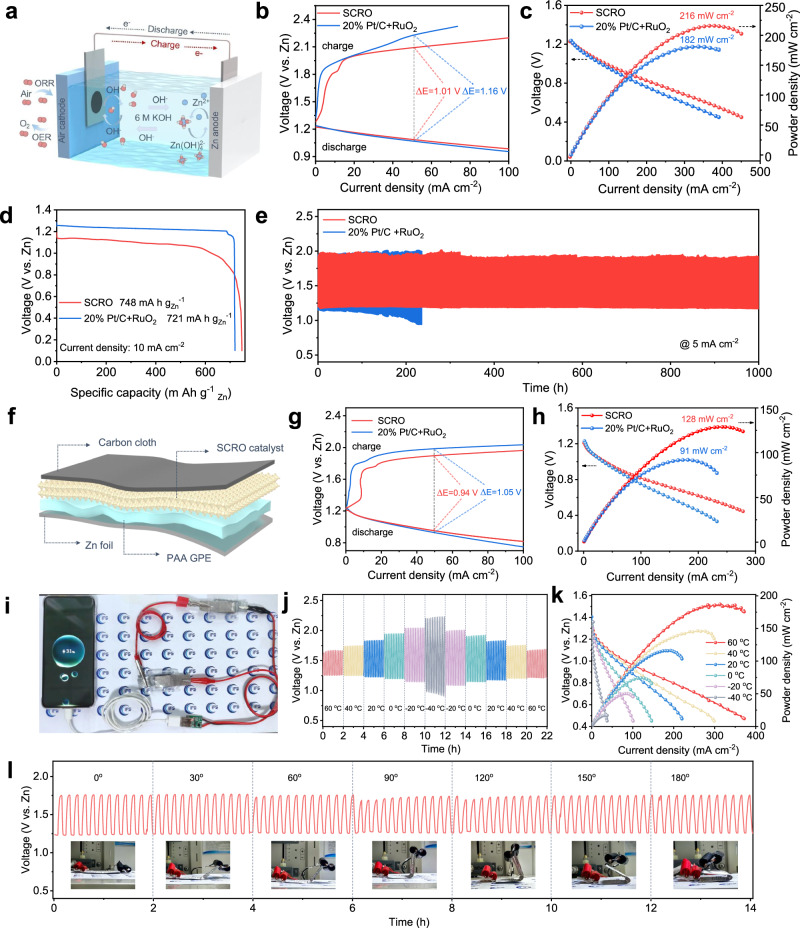
Practical zinc–air battery performance with multi-condition adaptability. **a** Schematic representation of rechargeable liquid ZABs. **b** Charge/discharge polarization curves of liquid ZABs based on SCRO and 20% Pt/C + RuO_2_. **c** Discharge polarization and corresponding power density curves. **d** Specific capacity curves at 10 mA cm^−2^. **e** Charge-discharge cycling curves at 5 mA cm^−2^ of liquid ZABs. **f** Schematic diagram of flexible solid-state ZABs. **g** Charge/discharge polarization curves, and **h** power density curves of the flexible ZABs based on SCRO and 20% Pt/C + RuO_2_ at room temperature. **i** Application for charging a mobile phone utilizing two series-connected SCRO-based ZABs. **j** Galvanostatic charge/discharge cycling of SCRO-based ZAB under various temperatures (from 60 to −40 °C) at 1 mA cm^−2^. **k** Power density curves of SCRO-based ZABs from 60 to −40 °C. **l** Cycling test of SCRO-based flexible ZAB in different bending states at current density of 1 mA cm^−2^. Insert images are photographs of flexible ZAB operating under different bending states. Source data for Fig. 6 are provided as a Source data file.

To investigate the practical applicability of SCRO in electrochemical energy devices, we assembled SCRO into the rechargeable ZABs. Figure 6a presents a schematic diagram of homemade liquid ZABs used in this study, with a Zn foil as anode, SCRO supported on carbon cloth as cathode and 6 M KOH + 0.2 M ZnCl_2_ as electrolyte. A reference ZAB was also assembled for comparison using the benchmark 20% Pt/C + RuO_2_ (1:1 mass ratio) catalyst as air cathode. Notably, the open-circuit voltage (OCV) reaches 1.41 V for liquid ZAB composed of SCRO (Supplementary Fig. 32), which is comparable to the benchmark 20% Pt/C + RuO_2_. Meanwhile, the ZAB based on SCRO exhibits higher discharge currents and lower charge–discharge voltage differences than commercial 20% Pt/C + RuO_2_, especially at higher current densities (Fig. 6b). On the basis of the discharge curve, the maximum power density of SCRO-based ZAB was calculated to be 216 mW cm^−2^, which is higher than 182 mW cm^−2^ of Pt/C + RuO_2_-based ZAB (Fig. 6c). Besides, the SCRO-based ZAB exhibits a high specific capacity of 748 mAh g_Zn_^−1^, comparable to that (721 mAh g_Zn_^−1^) of 20% Pt/C + RuO_2_-based device (Fig. 6d). According to the rate performance of SCRO-based ZAB device, the discharge voltage returns to the initial level when the current density gets back to 1 mA cm^−2^, suggesting the reversible rate behavior of SCRO (Supplementary Fig. 33). To assess the stability of SCRO-based ZAB, the cycling charge-discharge performance was explored at a current density of 5 mA cm^−2^ for 10 min each cycle, including 5 min for charging and another 5 min for discharge. As shown in Fig. 6e, the ZAB driven by SCRO electrocatalyst exhibits a small charge-discharge voltage gap of 0.77 V and robust operational durability up to 1000 h (6000 cycles) at 5 mA cm^−2^, surpassing the device based on 20% Pt/C + RuO_2_ catalyst (250 h), further confirming outstanding long-term durability of SCRO. Even under a higher current density of 10 mA cm^−2^, SCRO-based liquid ZAB still maintains stable operation for 300 h (1800 cycles) without noticeable decay (Supplementary Fig. 34). In addition, three integrated ZABs can provide adequate power to keep the light-emitting diode (LED) panel on, indicating its feasible practical application (Supplementary Fig. 35). More importantly, to realize dynamic combination of our trifunctional electrocatalyst, a self-powered water splitting system composed of two rechargeable ZABs and a two-electrode water splitting device was assembled, using SCRO as trifunctional electrocatalyst. Two SCRO-based ZABs connected in series exhibit an open-circuit voltage of 2.78 V (Supplementary Fig. 36), which can easily drive up the water splitting. As displayed in Supplementary Fig. 37 and Supplementary Movie 1, the bubbles of H_2_ and O_2_ could be apparently observed on the carbon papers at two electrodes, demonstrating the potential of SCRO as a promising trifunctional electrocatalyst for future integrated energy devices.

With the increased diversity of flexible and wearable electronics, there is an urgent demand for flexible energy storage devices with reliable power density and durability. Motivated by promising electrocatalytic activity and liquid ZAB performance of SCRO as demonstrated above, a rechargeable solid-state flexible ZAB was constructed using only SCRO coated carbon cloth as the air-cathode (catalyst loading: 0.5 mg cm^−2^), flexible Zn foil as anode and alkaline hydrogel as solid-state electrolyte (the ZAB configuration is schematically illustrated in Fig. 6f). The alkaline solid-state electrolyte was prepared by immersing the poly(acrylic acid) (PAA) hydrogel in a 6 M KOH + 0.2 M ZnCl_2_ solution for 24 h, which delivers considerable mechanical flexibility and ionic conductivity for flexible ZABs. As shown in Supplementary Fig. 38, SCRO-based flexible ZAB exhibits a high open-circuit voltage of 1.32 V, which is close to that of benchmark 20% Pt/C + RuO_2_-based ZAB. From charge-discharge polarization curves, the flexible ZAB with SCRO cathode exhibits a smaller charge voltage gap (0.94 V) compared to that (1.05 V) of 20% Pt/C + RuO_2_, implying the improved reversibility (Fig. 6g). Notably, SCRO-based flexible ZAB delivers a maximum power density of 128 mW cm^−2^, compared with the result for 20% Pt/C + RuO_2_ (91 mW cm^−2^) (Fig. 6h). At the current density of 1 mA cm^−2^, SCRO-based flexible ZAB maintains charge-discharge cycling for 35 h, outperforming that (15 h) of 20% Pt/C + RuO_2_ counterpart as well (Supplementary Fig. 39). Moreover, galvanostatic discharge test presents that SCRO-based ZAB can continuously discharge for 47 h at 1 mA cm^−2^ (Supplementary Fig. 40). Compared to liquid ZABs as discussed above, the solid-state flexible batteries display a relatively reduced durability and performance, primarily due to limited ionic conductivity, water loss, and the carbonation of PAA hydrogel electrolyte^53,54^. Nevertheless, the overall liquid and solid-state flexible ZAB performance of SCRO cathodes are comparable to many state-of-the-art electrodes for ZABs reported to date (Supplementary Table 5). With such outstanding performance, the solid-state flexible ZAB is able to power a mobile phone using two cells connected in series (Fig. 6i), which exhibits an open-circuit voltage of 2.55 V (Supplementary Fig. 41), demonstrating great potential of SCRO-based solid-state ZABs as a flexible energy supply. In order to evaluate the application potential of SCRO-based flexible ZABs in different scenarios, temperature adaptability and mechanical flexibility were further tested. When cycling at 60, 40, 20, 0, −20 and −40 °C, the flexible ZAB exhibits favorable voltage gap recovery ability (Fig. 6j). The SCRO-based ZAB presents a cycling charge-discharge voltage difference of 0.42 V at 60 °C. Due to the limited ionic conductivity of hydrogel electrolyte at low temperature, the voltage gap increases to 1.28 V with temperature dropping to −40 °C^55,56^. Specifically, the voltage gap of SCRO-based flexible ZAB can recover to 0.43 V as the temperature reverts to 60 °C. Besides, the peak power densities of SCRO-based flexible ZAB are higher than those of 20% Pt/C + RuO_2_ counterpart across temperature ranges from −40 to 60 °C (Fig. 6k and Supplementary Fig. 42). To investigate the deformation adaptability, the as-prepared flexible ZAB based on SCRO cathode is intentionally bent to various angles (from 0° to 180°). As illustrated in Fig. 6l, the charge and discharge voltage remain nearly constant at a current density of 1 mA cm^−2^ under different bending angles, demonstrating its excellent flexibility and application potentiality in wearable devices.

### Practical steady-state and fluctuating AEMWE performance

**Fig. 7:**
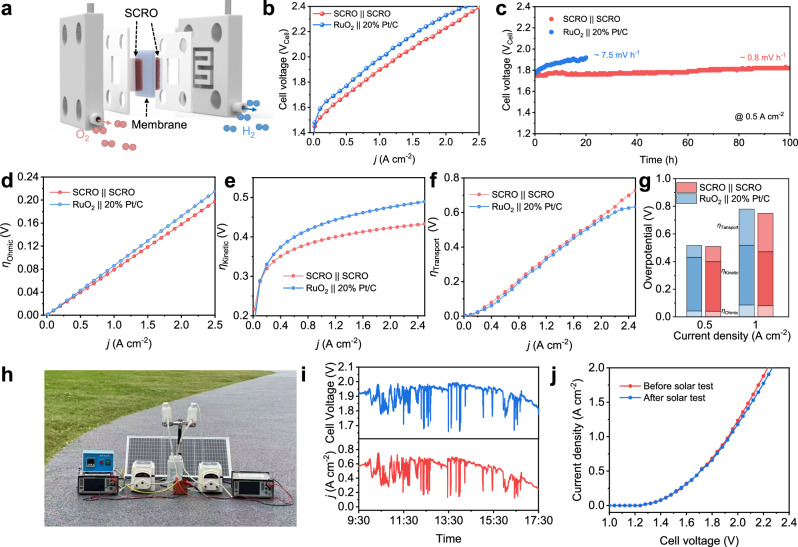
Practical steady-state and fluctuating AEMWE performance. **a** Schematic illustration of the AEMWE system. **b** Polarization curves and **c** durability tests of AEMWE with RuO_2_ || 20% Pt/C and bifunctional SCRO electrodes. Electrochemical analysis of overvoltage for AEMWE: **d** ohmic overvoltage (*η*_Ohmic_), **e** kinetic overvoltage (*η*_Kinetic_) and **f** transport overvoltage (*η*_Transport_). **g** The contributions of *η*_Ohmic_, *η*_Kinetic_ and *η*_Transport_ to the AEMWE with RuO_2_ || 20% Pt/C and bifunctional SCRO electrodes at 0.5 A and 1 A cm^−2^. **h** Photo image of integrated solar cell-AEMWE system. **i** Output voltage and current density obtained from the integrated solar cell-AEMWE system under natural sunlight conditions. **j** LSV curves of AEMWE before and after outdoor experiment. Source data for Fig. 7 are provided as a Source data file.

Encouraged by bifunctional electrocatalytic activity and durability of SCRO for OER and HER, we have constructed a lab-scale AEMWE with the architecture presenting in Fig. 7a, using SCRO as both anode and cathode in 1.0 M KOH solution. As shown in Fig. 7b, the steady polarization curves of AEMWE operated at 60 °C based on SCRO | | SCRO reaches the industrial current density of 1 A cm^−2^ at a lower cell voltage of 1.90 V than benchmark RuO_2_ || 20% Pt/C (1.99 V), demonstrating better AEMWE performance. With regard to operation durability, the bifunctional SCRO electrolysis cell can run robustly for 100 h under 0.5 A cm^−2^ at 60 °C (Fig. 7c). Specifically, the SCRO-based AEMWE system maintains stable performance with a decay rate of 0.8 mV h^−1^, much smaller than that (7.5 mV h^−1^) of RuO_2_ || 20% Pt/C-based AEMWE. As evidenced by post-mortem SEM and ICP analyses (Supplementary Figs. 43 and 44), the voltage decay of SCRO-based AEMWE may be attributed to gradual catalyst dissolution accompanied by partial delamination of the catalyst layer during long-term operation. To investigate the factors contributing to high electrochemical performance of AEMWE with bifunctional SCRO-based electrodes (Supplementary Fig. 45 and Supplementary Note 3), the breakdown of voltage losses is divided into three resistances: ohmic overvoltage (*η*_Ohmic_), kinetic overvoltage (*η*_Kinetic_) and transport overvoltage (*η*_Transport_)^37^. In Nyquist plot analysis, the high frequency intercept of electrochemical impedance spectroscopy (EIS) with real impedance axis represents *η*_Ohmic_ of the electrolysis system, primarily arising from the electronic and ionic resistance within the AEMWE system^57^. As seen in Fig. 7d, the *η*_Ohmic_ of SCRO | | SCRO-based AEMWE system is slightly lower than that of RuO_2_ || 20% Pt/C-based AEMWE system, indicating reduced ohmic resistance. Besides, electrochemical kinetic loss was evaluated to investigate the influence of electrocatalysts on electrode activation losses in AEMWEs. Figure 7e presents the *η*_Kinetic_ of AEMWE systems, in which SCRO | | SCRO displays a lower kinetic overvoltage than that of RuO_2_ || 20% Pt/C, consistent with the steady polarization curves present in Fig. 7b. In addition, two AEMWEs exhibit similar transport overvoltage, implying that the ionomer distribution within the electrode catalyst layer has a minimal influence on gas-liquid mass transport (Fig. 7f). Obviously, the performance difference of AEMWE between bifunctional SCRO and benchmark RuO_2_ || 20% Pt/C primarily originates from the ohmic overvoltage and kinetic overvoltage (Fig. 7g). Taken together, these results confirm that the enhanced performance of AEMWE equipped with bifunctional SCRO electrodes relative to RuO_2_ || 20% Pt/C is due to its reduced ohmic resistance and enhanced kinetic activity.

More importantly, to demonstrate the feasibility of carbon-free hydrogen generation driven by renewable electricity, bifunctional SCRO-based AEMWE device was integrated with a commercial solar cell (Fig. 7h). As shown in Fig. 7i, the performance of the integrated solar cell-AEMWE system was assessed through an outdoor experiment conducted from 09:30 to 17:30 under natural sunlight in Nanjing, China. As is well known, the intensity of sunlight fluctuates throughout a day, causing the corresponding variations in the output current and voltage of solar cells^58,59^. Even so, the AEMWE still maintains a rapid response and stable operation through the whole day under outdoor conditions, achieving consistent hydrogen production (Supplementary Movie 2). As previously reported, current fluctuation is a common issue in renewable energy-powered electrolyzers, which accelerate the degradation of electrode catalysts in AEMWEs^60–62^. Notably, LSV curves recorded before and after outdoor experiment exhibit negligible performance degradation of the AEMWE (Fig. 7j), indicating that SCRO electrode can serve as a viable bifunctional electrode candidate for practical AEMWEs application driven by fluctuating renewable energy. Hydrogen production driven by renewable energy sources is regarded as an environmentally friendly and sustainable energy pathway. This work pioneeringly demonstrates the feasibility of integrating solar cells with AEMWE equipped with bifunctional perovskite catalyst for renewable hydrogen production.

## Discussion

In summary, one single-phase SCRO with well-defined super-exchange double perovskite SCRO with unusual super-exchange effect was successfully synthesized, which demonstrates notable trifunctional electrocatalytic performance for alkaline OER, HER and ORR. Benefiting from its trifunctional electrocatalysis, we further assembled the SCRO into practical energy devices including ZABs and AEMWEs. As an air cathode of liquid ZABs, SCRO exhibits a high maximum power density of 216 mW cm^−2^, high specific capacity of 748 mAh g^−1^ and long lifespan up to 1000 h, outperforming the benchmark 20% Pt/C + RuO_2_. When employed as the air electrode for flexible solid-state ZABs, the corresponding batteries exhibit broad temperature and deformation adaptability. In addition, the AEMWE based on bifunctional SCRO electrodes exhibits comparable performance to the benchmark RuO_2_ || 20% Pt/C, achieving an industrial-level current density of 1 A cm^−2^ at a low cell voltage of 1.90 V and maintaining stable operation at 500 mA cm^−2^ for 100 h. When integrated the electrolyzer with a commercial solar cell, SCRO-based AEMWE demonstrates a stable and rapid response under fluctuating electricity. Dramatically, the combination of multiple in situ characterizations and microscopic techniques comprehensively reveal the spatiotemporal reconstruction behavior of SCRO and identify the real active components under different electrocatalytic reactions. This work provides instructive insights into the design and development of high-efficiency trifunctional electrocatalysts for futuristic energy devices with multi-condition adaptability.

## Methods

### Chemicals

Strontium carbonate (Sinopharm Chemical Reagent Co., Ltd, SrCO_3_, ≥99.5%), cobalt oxide (Sinopharm Chemical Reagent Co., Ltd, Co_3_O_4_, ≥99.9%), ruthenium oxide (Alfa Aesar, RuO_2_, ≥99.9%), potassium hydroxide (Aladdin Reagent (Shanghai) Co., Ltd, KOH, analytical reagent), oxygen (Nanjing Special Gas Factory Co., Ltd, O_2_, 99.999%), argon (Nanjing Special Gas Factory Co., Ltd, Ar, 99.999%) and Nafion solution (Shanghai Geli New Energy Technology Co., Ltd, 5 wt%). Deionized (DI) water was produced using a Millipore ultrapure water system.

### Catalyst synthesis

SCRO, SCO and SRO were synthesized via a solid-phase reaction method. Taking the synthesis of SCRO as an example, stoichiometric amounts of SrCO_3,_ Co_3_O_4_ and RuO_2_ powders were firstly dispersed in ethanol media and mixed using a high-energy ball mill (Fritsch, Pulverisette 6) under 400 rpm for 1 h. The homogeneous precursor mixture was then dried and calcined at 1100 °C in air for 10 h to obtain SCRO powders.

### Basic characterizations

XRD patterns were acquired using a Bruker D2 PHASER X-ray diffractometer operating at 30 kV with filtered Cu K*α* radiation (λ = 1.54184 Å). Rietveld refinements were performed using FullProf software. SEM images were recorded via a TESCAN LYRA3 GM scanning electron microscope. HRTEM, STEM and elemental mapping images were obtained using JEM-F200 operating at 200 kV. Nitrogen adsorption isotherms were recorded on JW-BK300 at 77 K and the specific surface areas were calculated by the BET methods. XAS data at the Co and Ru *K*-edges were collected at the Taiwan Photon Source (TPS) 44 A beamline of the National Synchrotron Radiation Research Center (NSRRC) in Taiwan, which employs a quick-scanning monochromator (Q-mono) system for real-time XAS data collection with one spectrum of 0.5 s and provides high temporal resolution down to the millisecond level. In situ XAS experiments were performed under the desired conditions with a custom-built operando XAS instrument. Ex-situ XAS at the Co *L*_3_-and Ru *L*_3_-edge were carried out Taiwan Light Source beamline 11 A of the NSRRC. In situ SR-based XRD data were acquired at the 01C2 beamline in the NSRRC. In situ XRD measurements were conducted in a self-assembled Teflon cell sealed with a Kapton tape window (1 cm × 1 cm). The incident X-ray beam was transmitted through the Kapton window and the electrolyte to collect data signals. All in situ experiments were conducted in a standard three-electrode system using a CHI 660 electrochemical workstation.

### Electrochemical measurements

All electrochemical tests (OER, HER, ORR) were performed with rotating disk electrode (RDE) or rotating ring-disk electrode (RRDE) in a three-electrode system using CHI760E electrochemical workstation (Shanghai Chenhua) at room temperature. A glassy carbon RDE (5 mm in diameter, Pine, geometric area: 0.196 cm^2^) or a glassy carbon RRDE (5.6 mm in diameter, Pine, geometric area: 0.246 cm^2^) was used as the working electrode. The Hg/HgO electrode (filled with 1.0 M KOH solution) was employed as a reference electrode. The counter electrode was a Pt wire in the ORR and OER measurement or a graphite rod in the HER. Details of the working electrode preparation are as follows: 10 mg of catalyst and 10 mg of carbon black were dispersed in a mixed solution consisting of 1 mL ethanol and 50 μL Nafion solution (5 wt%) and ultrasonicated for at least 60 min. Next, the homogeneous ink was pipetted onto the glassy carbon electrode surface and dried in air at room temperature, yielding a working electrode with a catalyst loading of ~0.34 mg cm^−2^. OER polarization curves were carried out in an O_2_-saturated 1.0 M KOH solution with a scan rate of 5 mV s^−1^. HER polarization curves were carried out in an Ar-saturated 1.0 M KOH solution with a scan rate of 5 mV s^−1^. ORR polarization curves were carried out in an O_2_-saturated 0.1 M KOH solution at 1600 rpm and a scan rate of 5 mV s^−1^. The solution resistance (*R*_sol_) was determined by electrochemical impedance spectroscopy (EIS), and OER and HER polarization curves were *iR*-corrected accordingly. All three-electrode measurements were performed in a five-neck glass electrochemical cell containing 150 mL of electrolyte. All the potentials in this study were calibrated to the reversible hydrogen electrode (RHE). The Hg/HgO reference electrode was calibrated against the RHE in H_2_-saturated KOH solution using a Pt electrode. Linear sweep voltammetry was performed three times around the hydrogen evolution/oxidation equilibrium potential, and the average potential at zero current was taken as 0 V versus RHE. The measured conversion factors were 0.84287 V in 0.1 M KOH (pH = 12.79 ± 0.02) and 0.90906 V in 1.0 M KOH (pH = 13.84 ± 0.05). All electrolyte solutions were freshly prepared before electrochemical measurements using DI water and the corresponding salts. The prepared electrolytes were stored in sealed polypropylene bottles at room temperature and used within the same day. The ECSA was estimated from the double-layer capacitance (*C*_dl_) measurements. Cyclic voltammetry was performed in a non-faradaic potential region at different scan rates (20, 40, 60, 80, 100 mV s^−1^). The *C*_dl_ value was obtained from the slope of Δ*j*/2 at a selected potential versus scan rate, the formula for ECSA calculation is given below:

1 $${{\rm{ECSA}}}={C}_{{{\rm{dl}}}}/{C}_{{{\rm{s}}}}$$

where *C*_s_ is the specific capacitance of a flat electrode surface, which is generally in the range of 20–60 μF cm^−2^. In our calculations, a commonly used *C*_s_ value of 40 μF cm^−2^ was adopted.

### Liquid ZAB measurements

In order to evaluate the performance of SCRO, a homemade ZAB was assembled. For aqueous ZAB, a polished zinc foil was used as the anode (electrode size of 2.0 cm^2^, thickness: 0.5 mm), a catalyst-loaded carbon cloth was used as the air cathode (size of carbon cloth: 3.14 cm^2^, catalyst-coated circular area: 0.196 cm^2^, catalyst loading mass of 0.5 mg cm^−2^), and a solution of 6 M KOH with 0.2 M ZnCl_2_ was used as the electrolyte. No separator was used in the aqueous ZAB cell. The battery was operated under ambient static air without external air flow, and no electrolyte flow was applied. Charge/discharge and power density tests were performed on CHI 760E electrochemical workstation (Shanghai Chenhua), and capacity, rate performance and constant current cycling tests were performed on CT-4008Tn-5V1A-S1-F battery testing system (Shenzhen NEWWARE). The tests were conducted at room temperature, approximately 25 °C and the ambient relative humidity during the tests was approximately 42%. The stability of rechargeable ZAB was tested with a charge/discharge cycle interval of 10 min (5 min charge, 5 min discharge). The formulas for specific capacity and power density are given below:

2 $${C}={I}\times {t}/{M}$$

where *C*, *I*, *t* and *M* are specific capacity (mAh g_Zn_^−1^), discharging current, testing time and the mass of consumed zinc, respectively.

3 $${P}={I}\times {V}$$

where *P* and *V* are power density (W cm^−2^) and the measured voltage, respectively.

### Solid-state flexible ZAB measurements

Solid-state ZABs used a polished flexible zinc foil as the anode and a carbon cloth as the air cathode, which is loaded with 0.5 mg cm^−2^ of electrocatalyst. The PAA gel immersed in 6 M KOH containing 0.2 M ZnCl_2_ for 24 h was used as alkaline solid-state electrolyte. Batteries were tested with CHI 760E electrochemical workstation (Shanghai Chenhua) and CT-4008Tn-5V1A-S1-F battery testing system (Shenzhen NEWWARE). Tests of flexible solid-state batteries at different temperatures were performed in BPH-060C thermostatic test chamber (Shanghai YIHENG). Unless otherwise stated, the tests were conducted at room temperature, approximately 25 °C and the ambient relative humidity during the tests was approximately 42%. For the mobile-phone charging demonstration, two solid-state flexible ZABs were connected in series to deliver an open-circuit voltage of 2.55 V, and the output was further boosted to a regulated 5.0 V through a DC–DC converter for charging the mobile phone.

### AEMWE testing

Lab-scale AEMWE comprises an anode (electrode size of 4.0 cm^2^), a cathode (electrode size of 4.0 cm^2^), an anion exchange membrane (AEM, MTCP-50, thickness: 50 μm, membrane size of 6.25 cm^2^), gaskets, flow channels, current collectors and end plates. The membrane was immersed in 1.0 M KOH solution for at least 24 h before use. The catalyst coated substrate (CCS) technology was adopted to prepare the electrodes. The cathode and anode ink solution was prepared by mixing 200 mg SCRO catalysts with 40 mg conductive carbon black and 300 mg 5 wt.% ionomer solution in 16 g solvent (DI water:isopropyl alcohol = 3:8 at mass ratio) and ultrasonicated at room temperature for 1 h. The as-prepared cathode and anode catalyst inks were then spray-deposited onto the Ni felt using an ultrasonic spraying instrument, with the mass loading of cathodes and anodes being 1 mg cm^−2^. The AEMWE was assembled by integrating cathode, membrane, and anode between two Ti bipolar plates with a torque of 8 N m to complete an AEMWE device. Fluorine rubber gaskets with a thickness of 3 mm were placed on both the anode and cathode side to ensure that the electrodes were not over-compressed. Eight fastening bolts were tightened diagonally to ensure proper sealing of the cell. RuO_2_ || 20 wt% Pt/C system was also prepared for comparison. 1.0 M KOH solution was used as the electrolyte and supplied at a flow rate of 35 mL min^−1^ for the AEMWE system. The operating temperature was set at ~60 °C. Polarization curves of AEMWE devices were obtained by using a DC power supply (Fueiceel® DC Power Supply F3020). The voltage and current outputs of integrated solar cell-AEMWE system were recorded using a Keithley 2450 source meter.

## Supplementary information

Supplementary information

Description of Additional Supplementary Files

Supplementary Data 1

Supplementary Movie 1

Supplementary Movie 2

Transparent Peer Review file

## Source data

Source Data

## Data Availability

The data supporting the findings of this study are presented in the paper, Supplementary Information, Supplementary Data 1 and the Source data file. Source data are provided with this paper.
