# Supplementary material for: Trifunctional electrocatalyst with accurate surface reconstruction for zinc-air batteries and water electrolyzers
Source: Nat Commun. 2026 Jun 23;17:7890. doi: 10.1038/s41467-026-74714-5 (PMC13444124; doi:10.1038/s41467-026-74714-5)
Supplement: Supplementary file 1 [file 41467_2026_74714_MOESM1_ESM.pdf]

Supplementary Information

Trifunctional Electrocatalyst with Accurate Surface Reconstruction for Zinc Air Batteries and Water Electrolyzers

I. Supplementary Figures

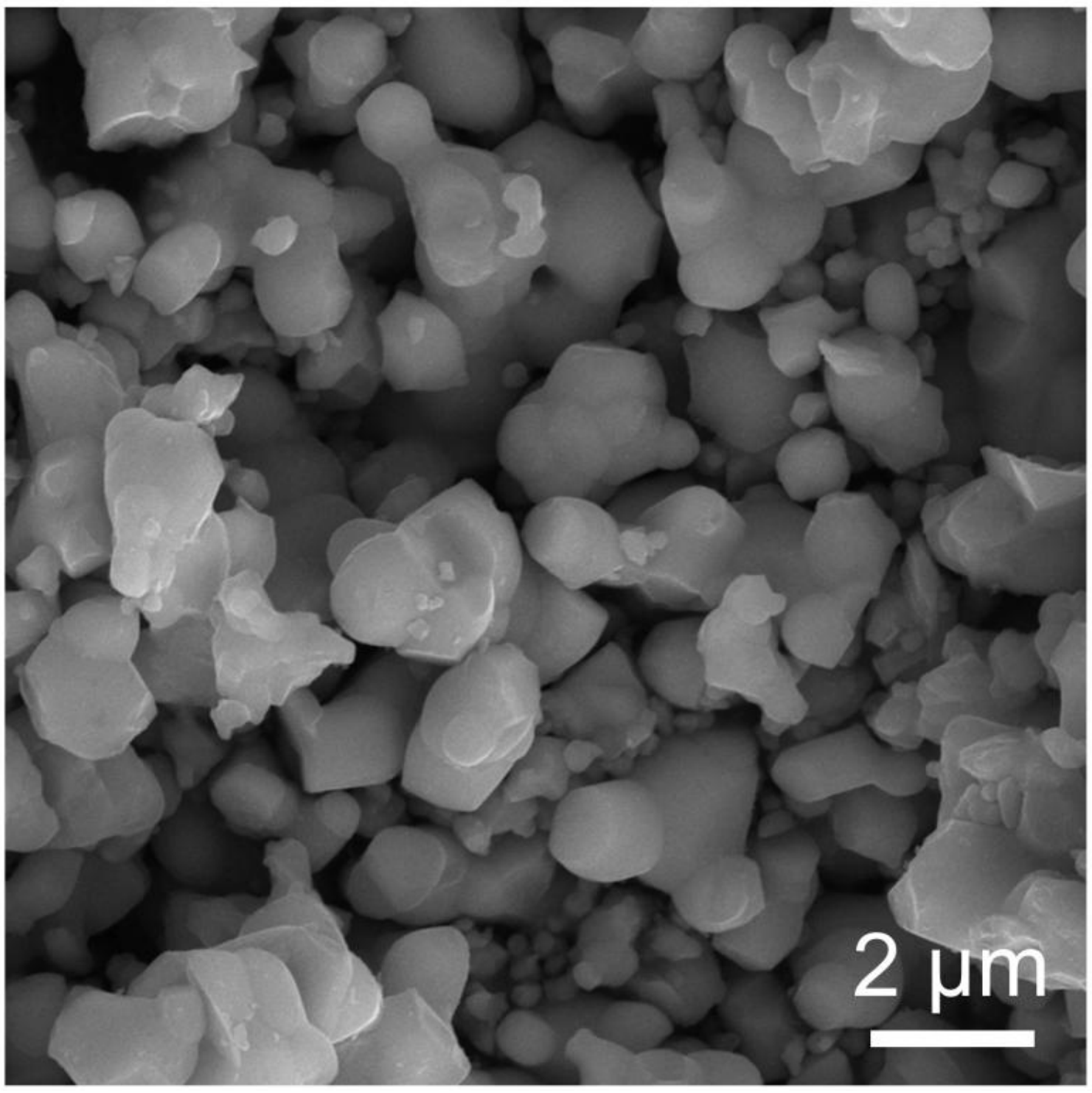

Supplementary Figure 1. SEM image of SCRO.

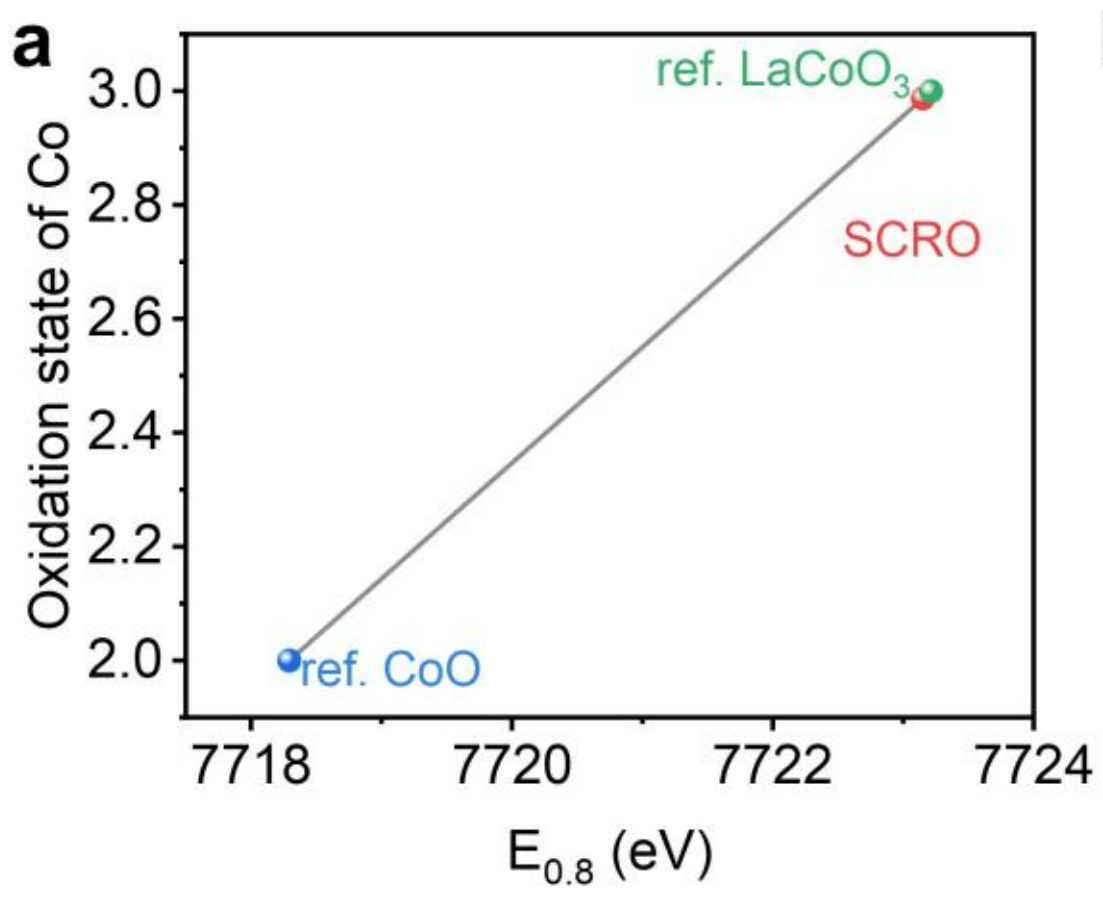

Supplementary Figure 2. Oxidation states of (a) Co and (b) Ru in SCRO as a function of edge energy at a 0.8/0.6 jump height of ($E_{0.8}$/$E_{0.6}$), obtained from the normalized XANES spectra at the Co/Ru *K*-edge.

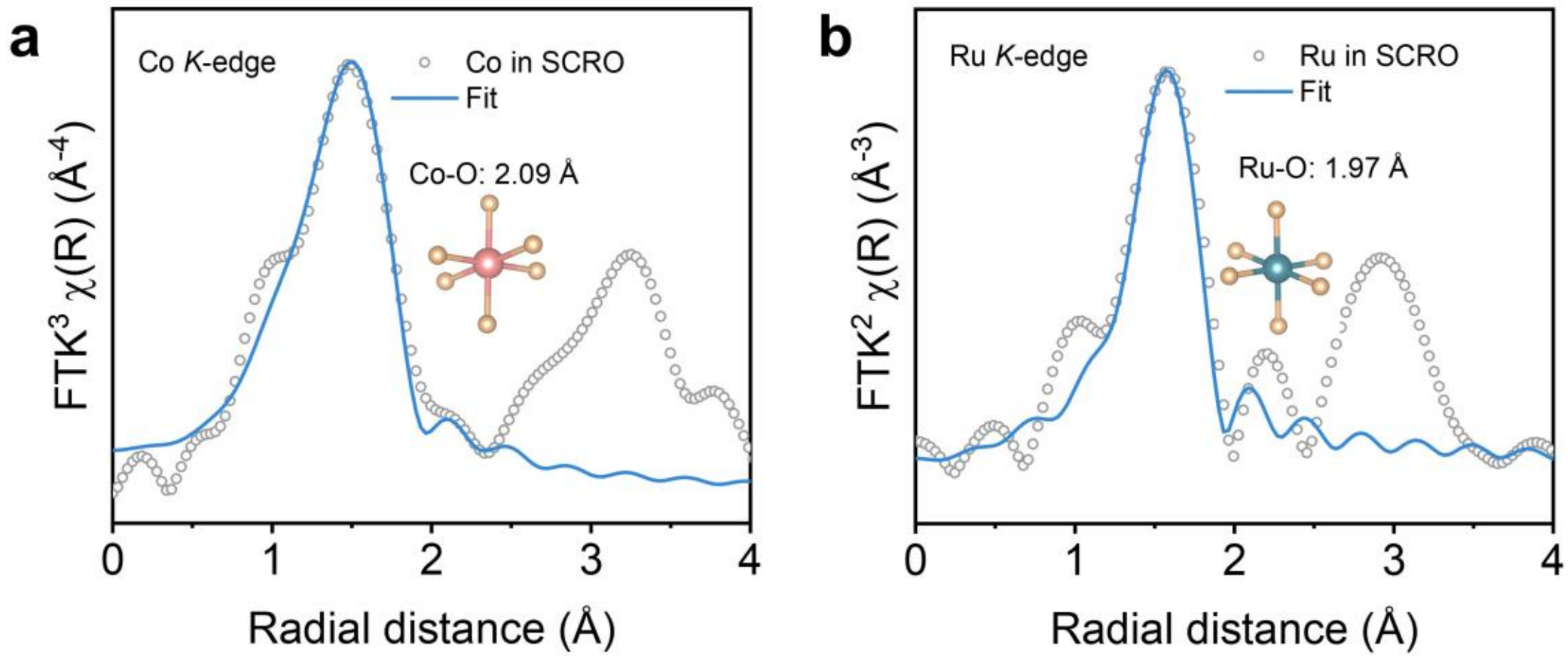

Supplementary Figure 3. Fourier transform (FT) EXAFS fitting curves of SCRO at (a) Co and (b) Ru *K*-edge.

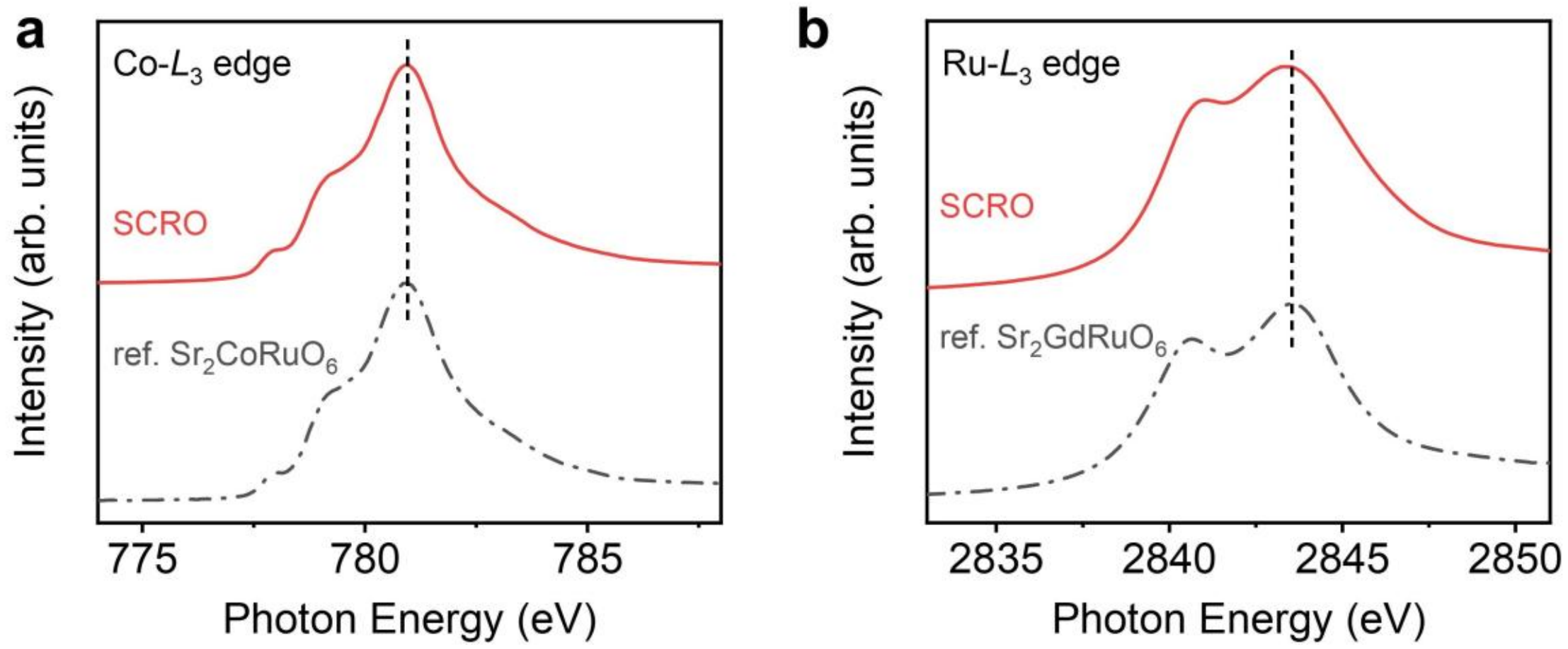

Supplementary Figure 4. (a) Co L_3-edge XAS spectra of SCRO together with that of standard Sr_2CoRuO_6 as references for HS-Co^{3+}. (b) Ru L_3-edge of the pristine SCRO together with that of Sr_2GdRuO_6 as references for Ru^{5+}.

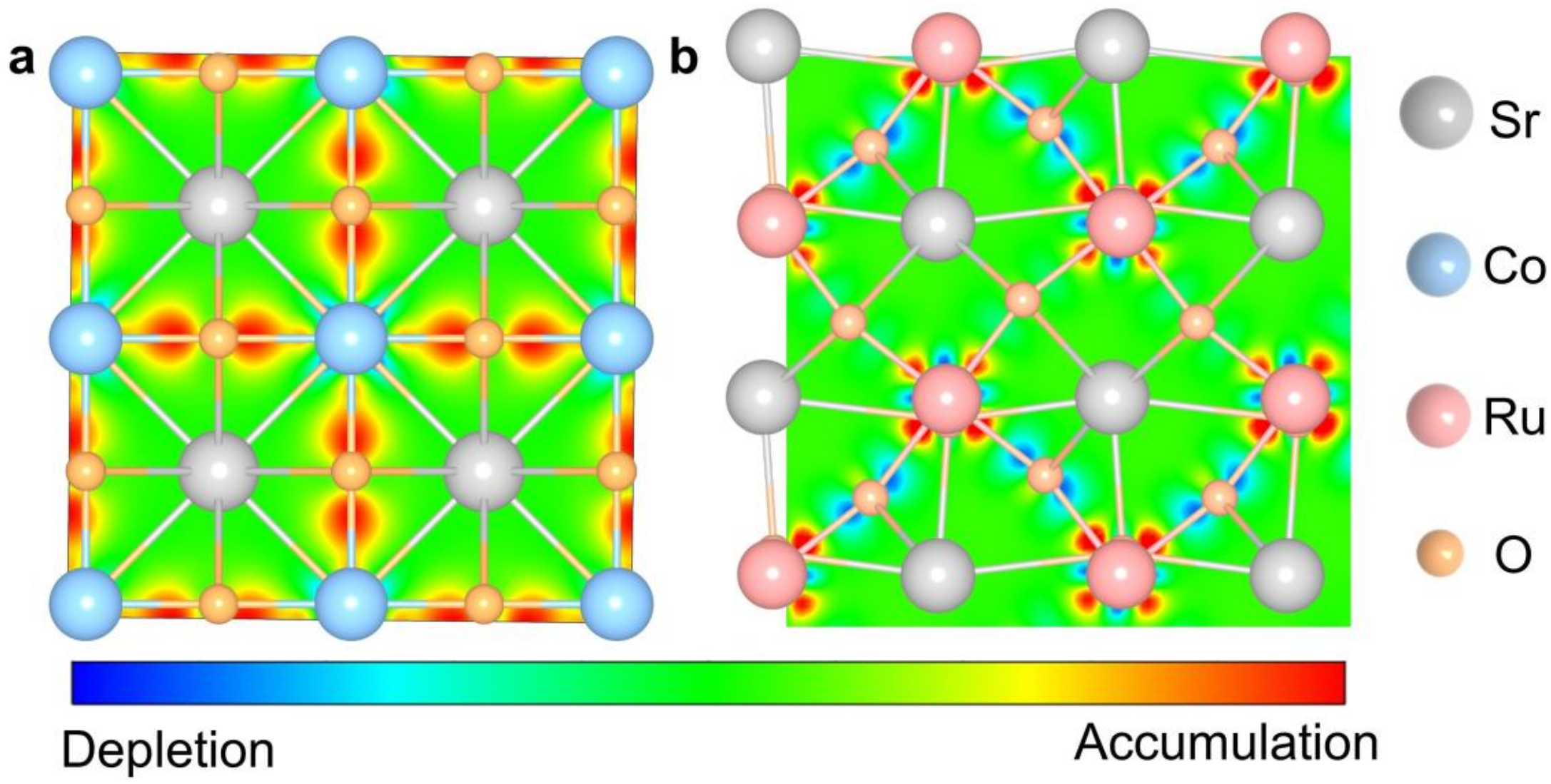

Supplementary Figure 5. Charge distribution plots of (a) SCO and (b) SRO.

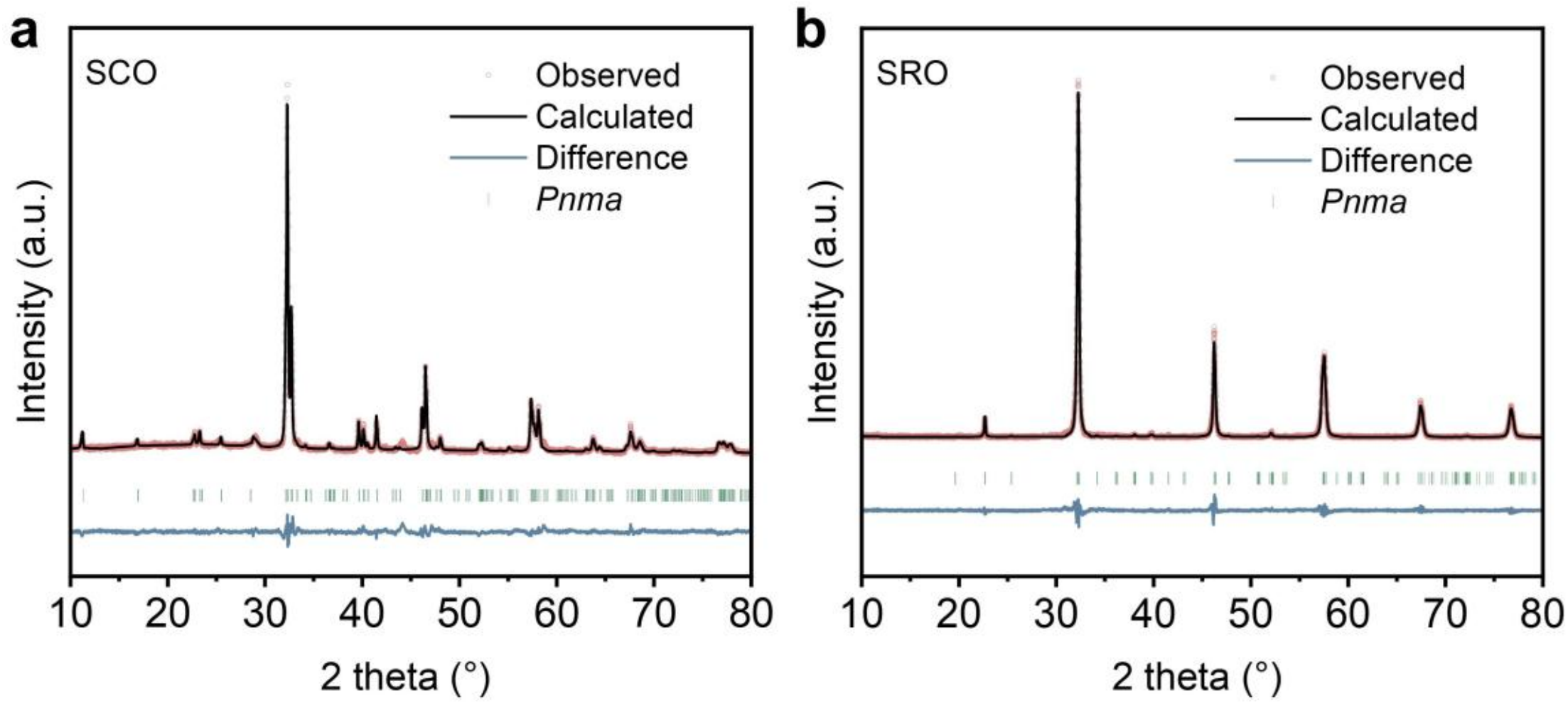

Supplementary Figure 6. Refined XRD profiles of (a) SCO and (b) SRO.

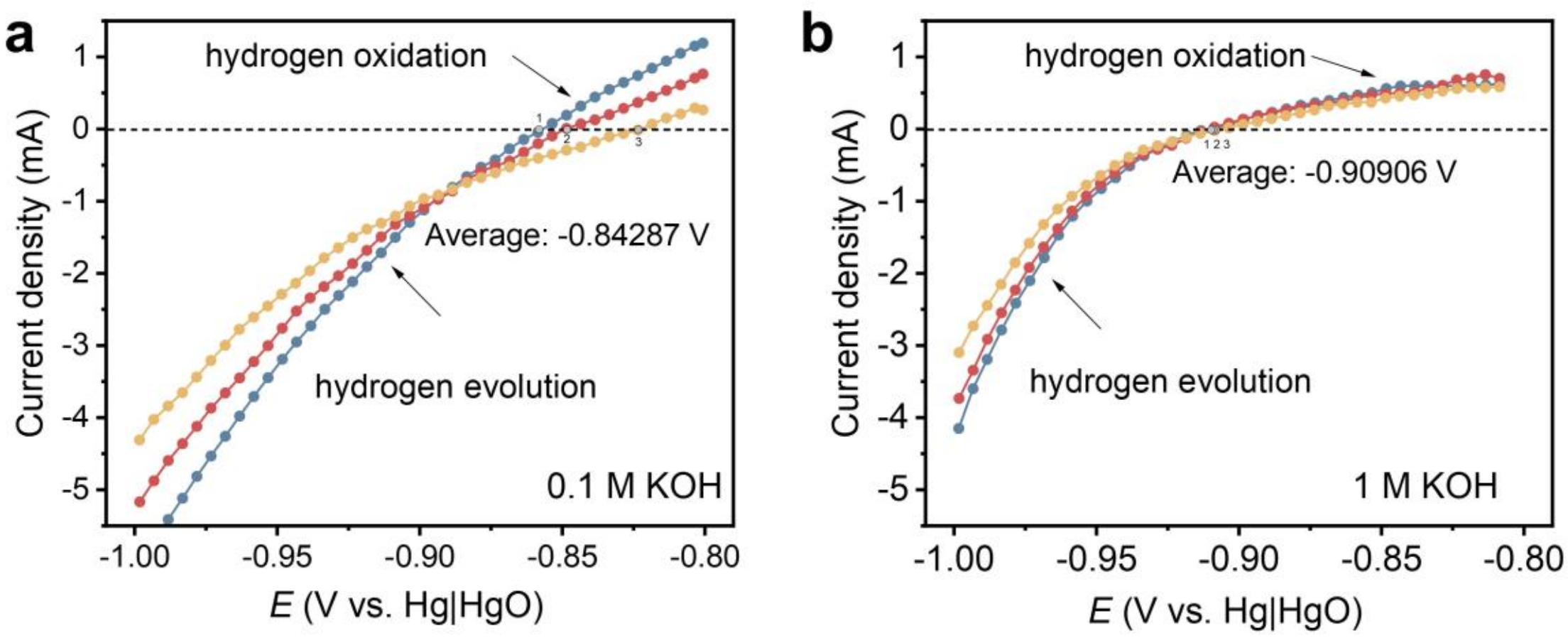

Supplementary Figure 7. RHE calibration of the Hg|HgO reference electrode in (a) 0.1 M KOH and (b) 1 M KOH.

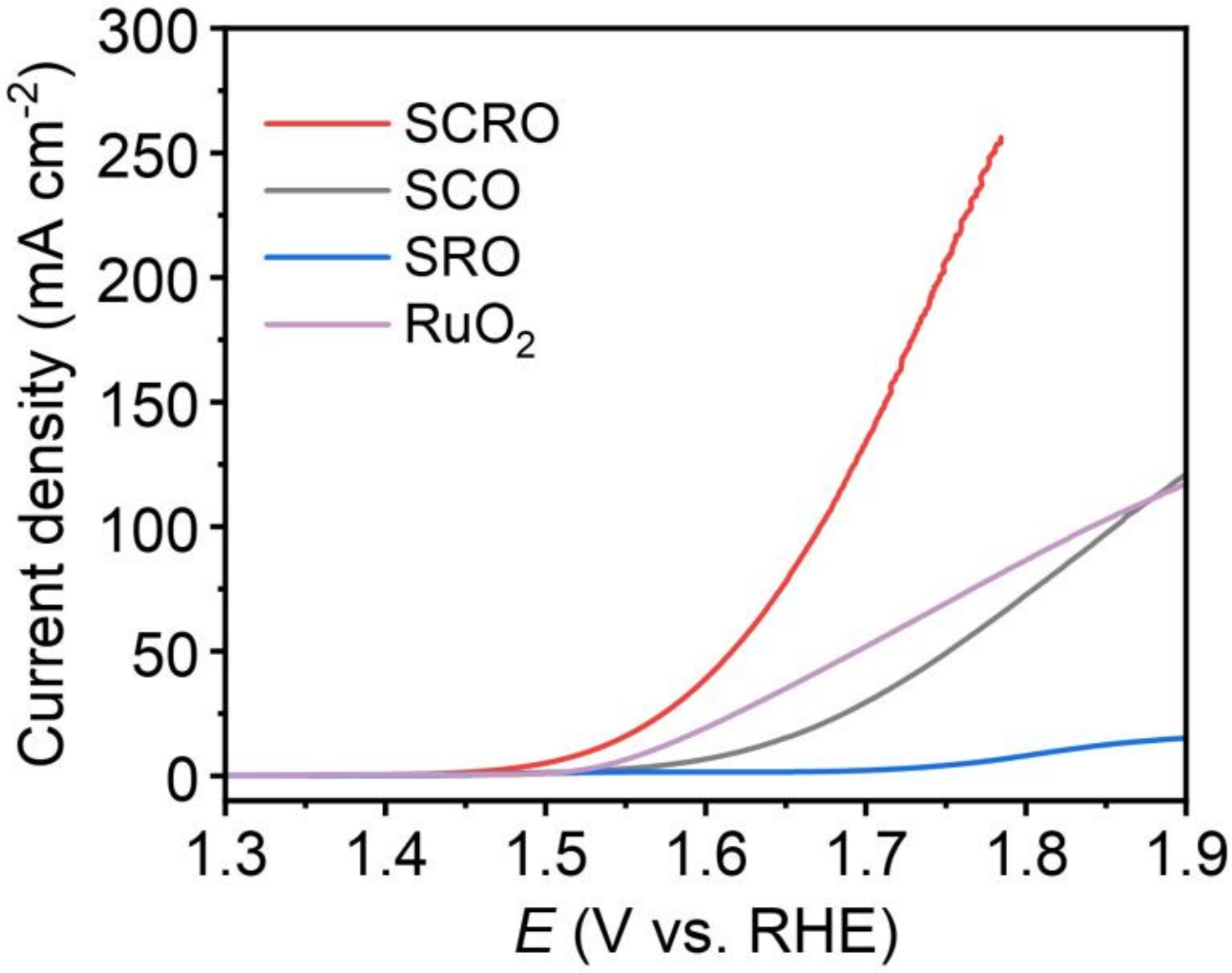

Supplementary Figure 8. Non-*iR*-corrected OER polarization curves of SCRO, SCO, SRO and RuO_2 in O_2-saturated 1 M KOH.

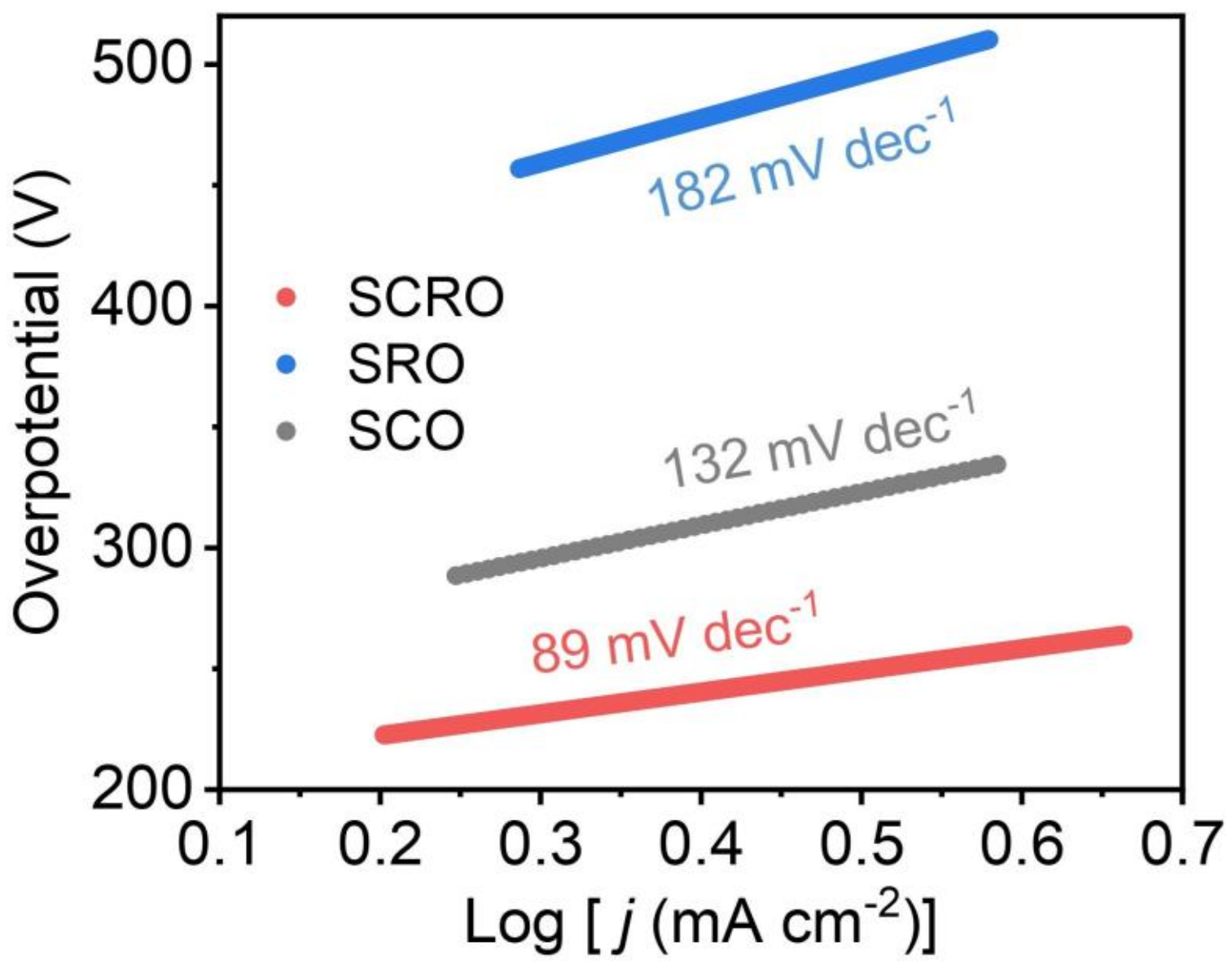

Supplementary Figure 9. Tafel plots for SCRO, SCO and SRO for OER in 1 M KOH.

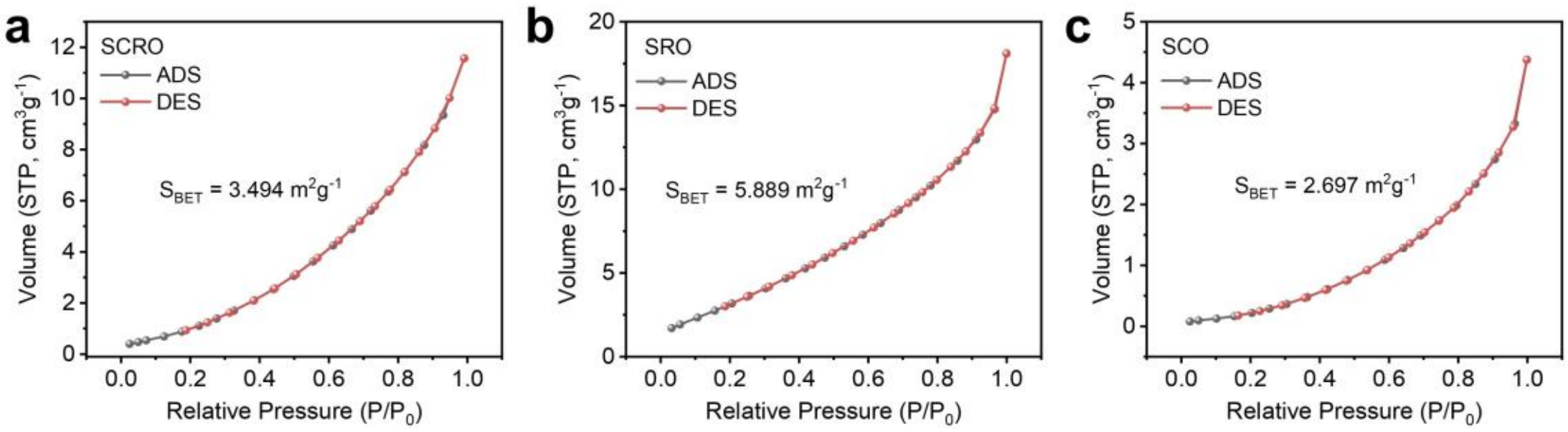

Supplementary Figure 10. N_2 adsorption-desorption isotherm of (a) SCRO, (b) SCO (c) SRO and the corresponding specific surface areas as estimated from Brunauer-Emmett-Teller (BET) measurements.

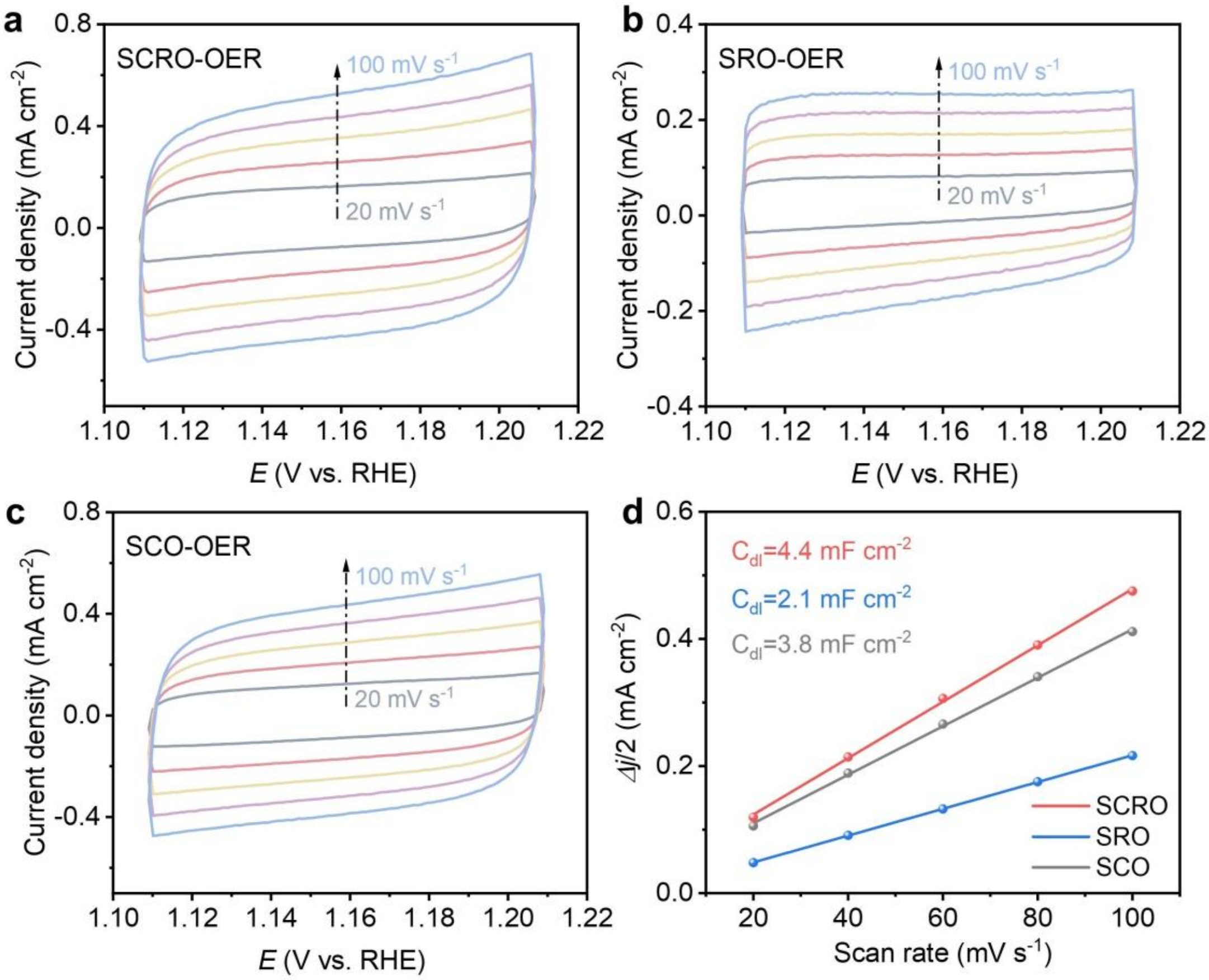

Supplementary Figure 11. Electrochemical CV scans of (a) SCRO, (b) SRO and (c) SCO in the non-faradaic potential region for OER. (d) Calculated double-layer capacitances extracted from the linear fitting of the capacitive currents versus CV scan rates for SCRO, SRO and SCO.

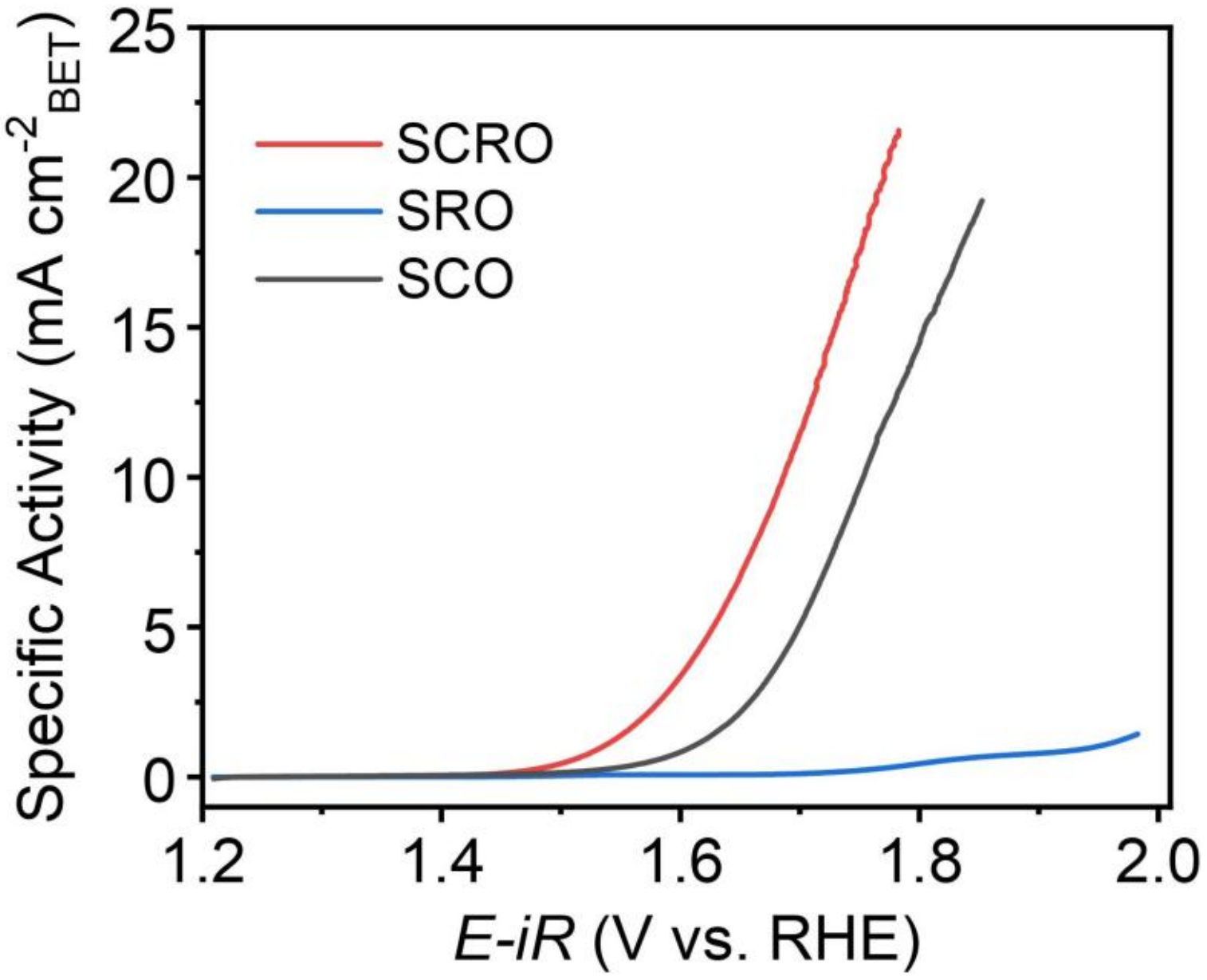

Supplementary Figure 12. Specific activity for OER normalized to BET of SCRO, SCO and SRO as a function of applied potentials.

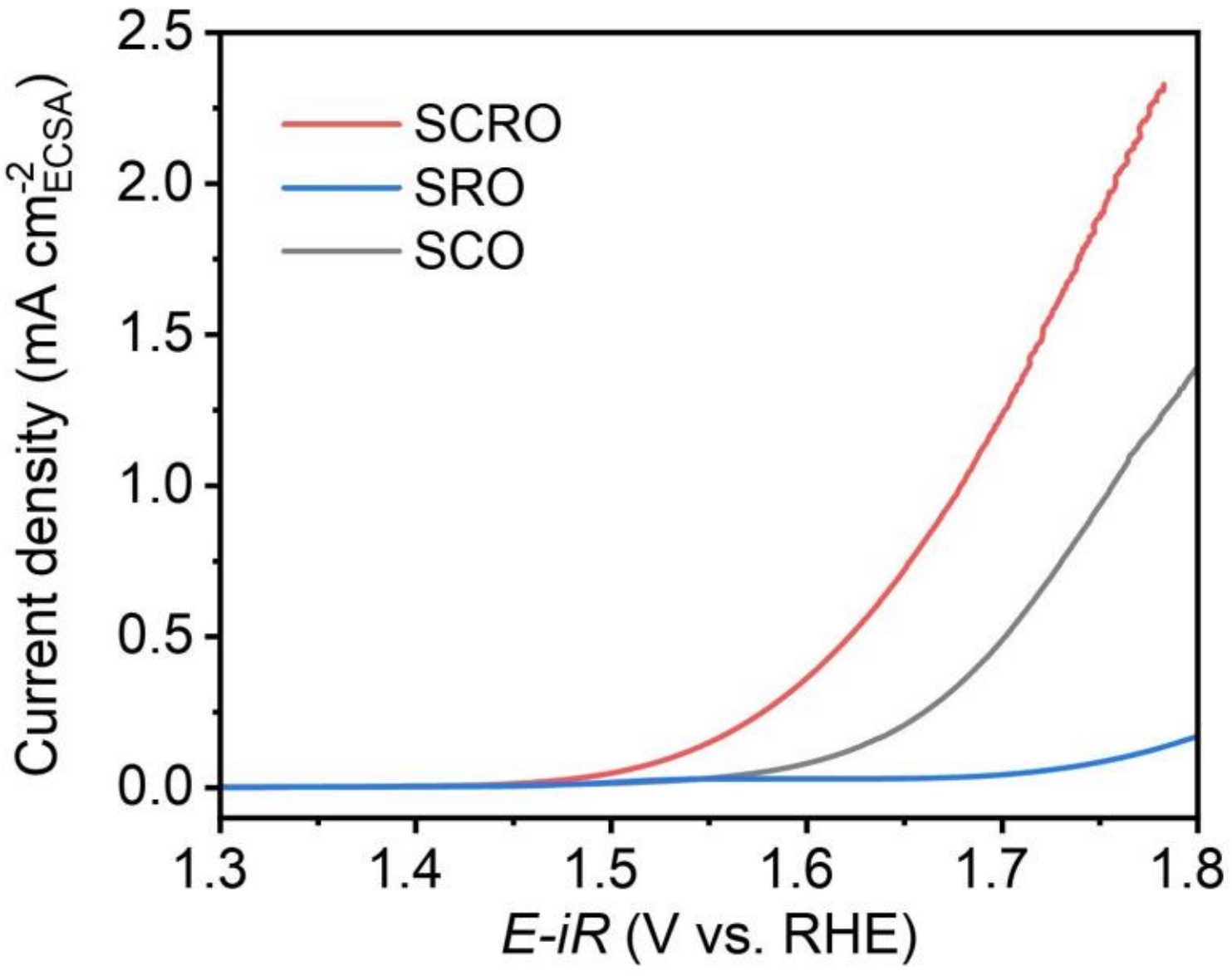

Supplementary Figure 13. Specific activity for OER normalized to ECSA of SCRO, SCO and SRO as a function of applied potentials.

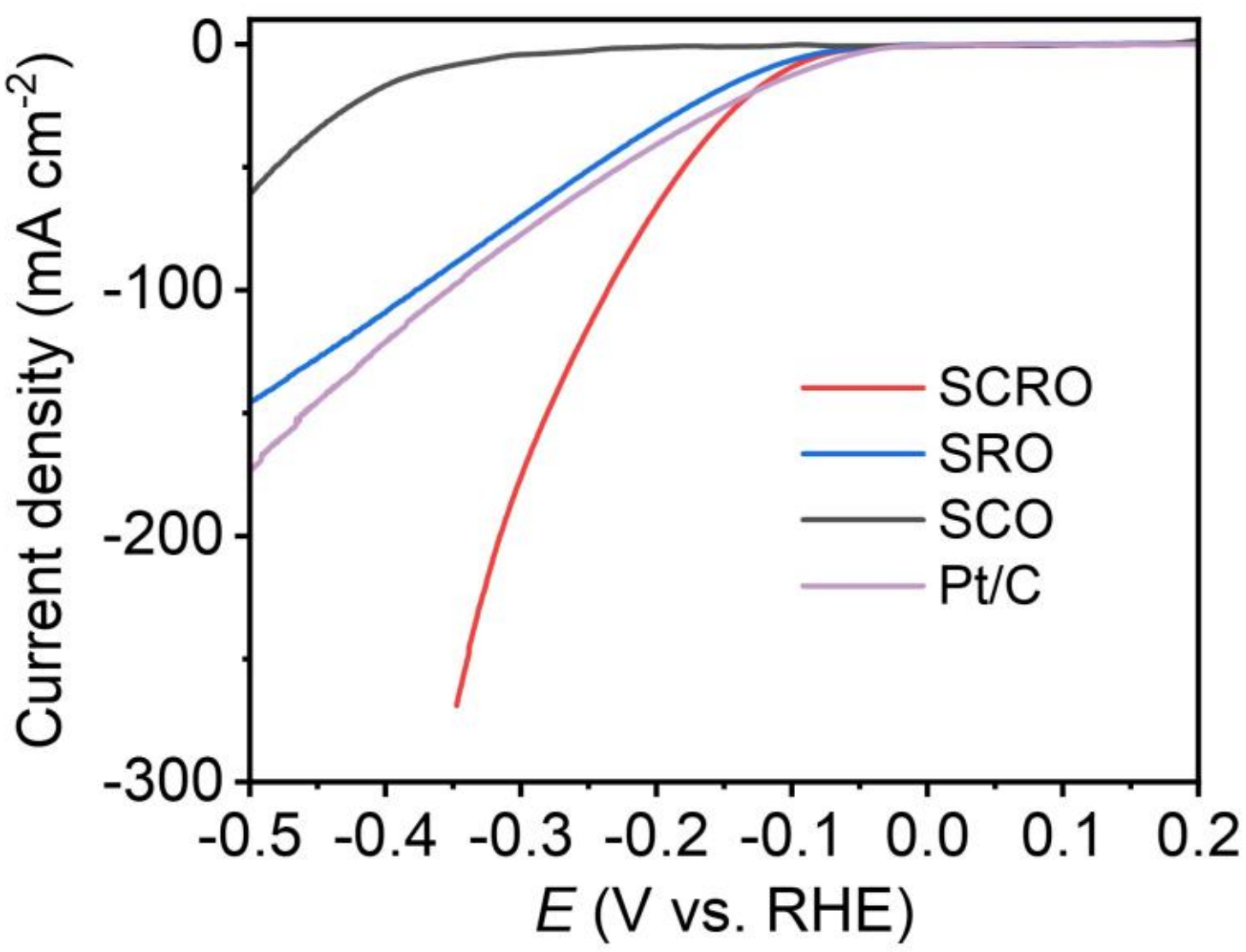

Supplementary Figure 14. Non-*iR*-corrected HER polarization curves of SCRO, SRO, SCO and Pt/C in Ar-saturated 1 M KOH.

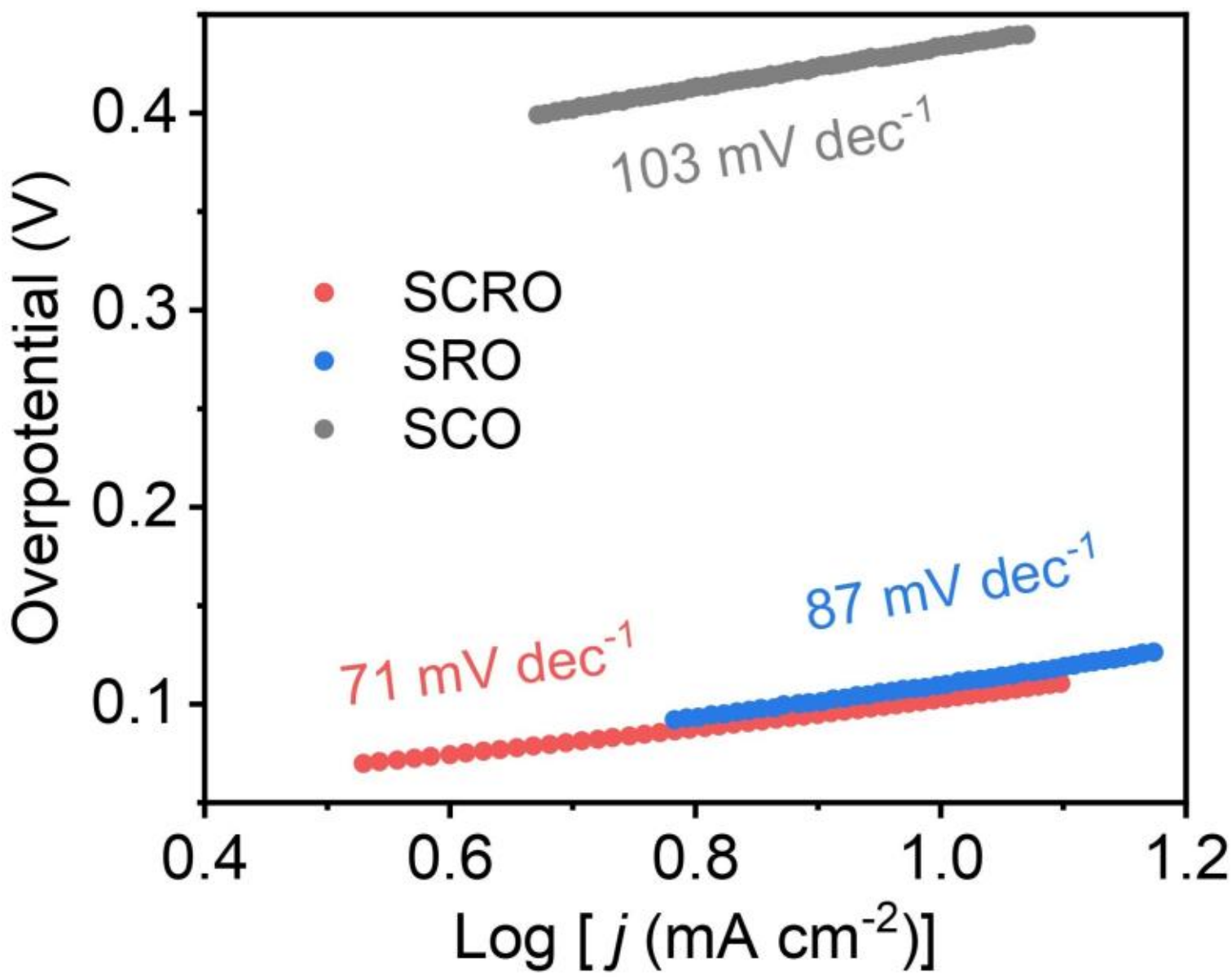

Supplementary Figure 15. Tafel plots for SCRO, SCO and SRO for HER in 1.0 M KOH.

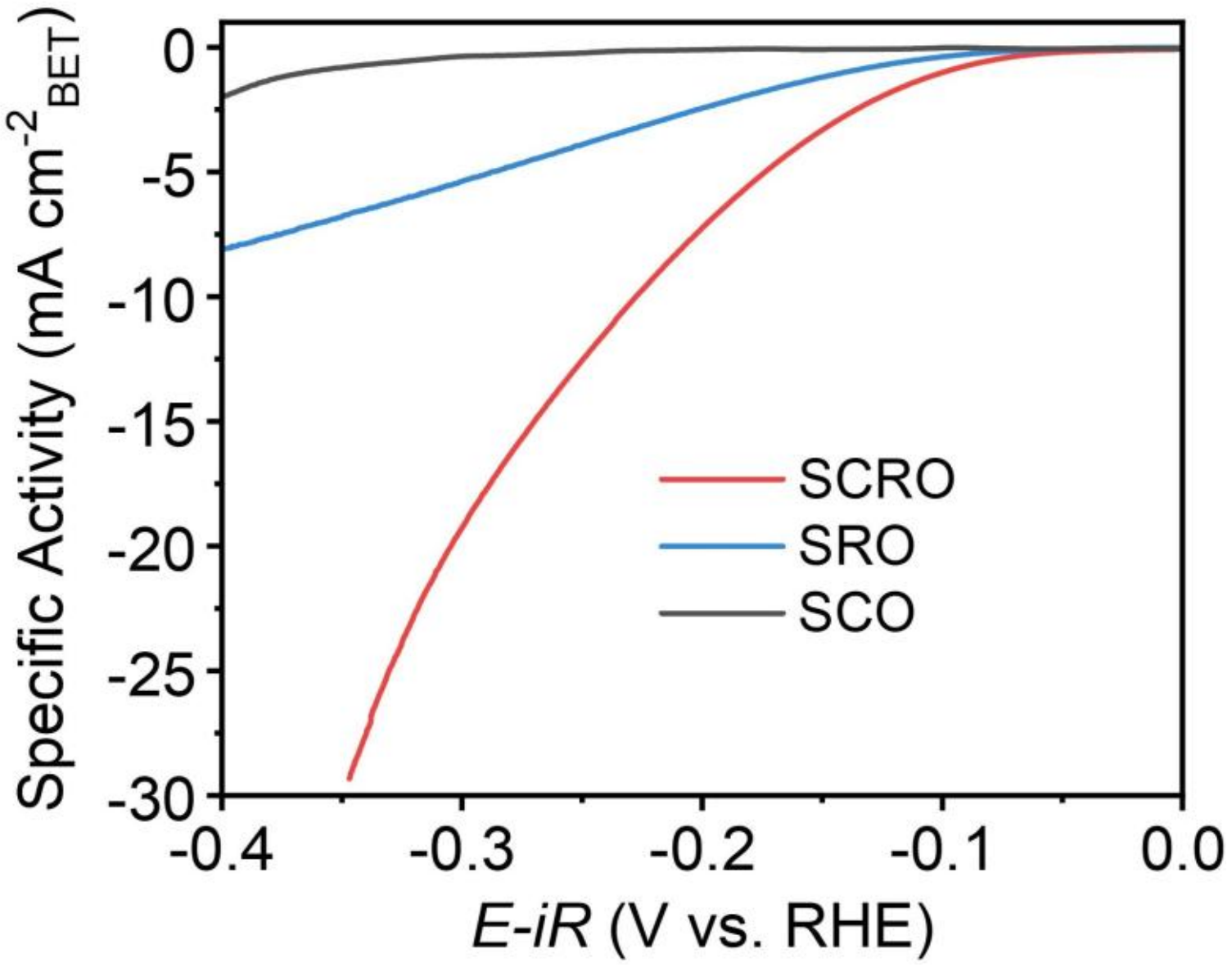

Supplementary Figure 16. Specific activity for HER normalized to BET of SCRO, SCO and SRO as a function of applied potentials.

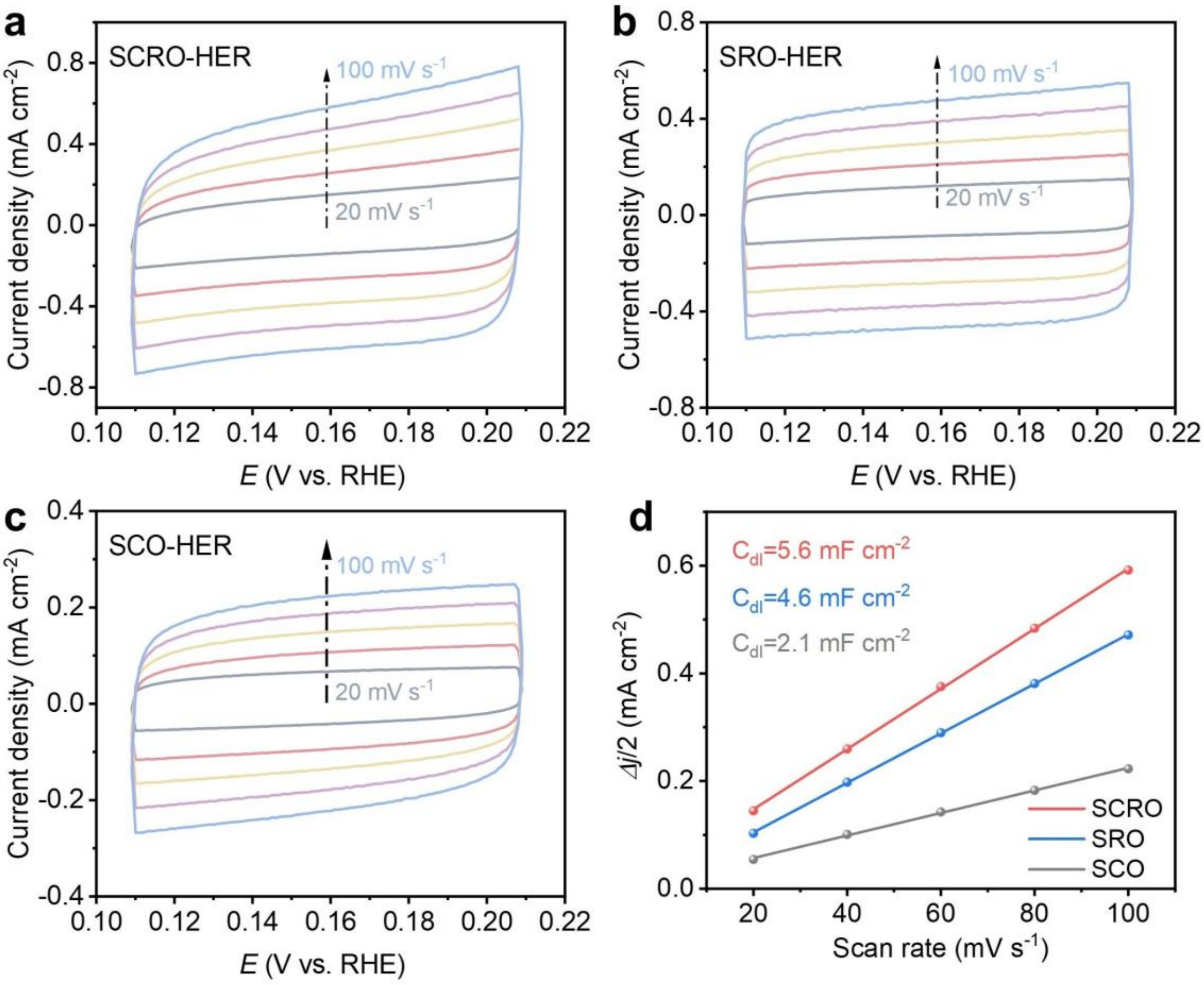

Supplementary Figure 17. Electrochemical CV scans of (a) SCRO, (b) SRO and (c) SCO in the non-faradaic potential region for HER. (d) Calculated double-layer capacitances extracted from the linear fitting of the capacitive currents versus CV scan rates for SCRO, SRO and SCO.

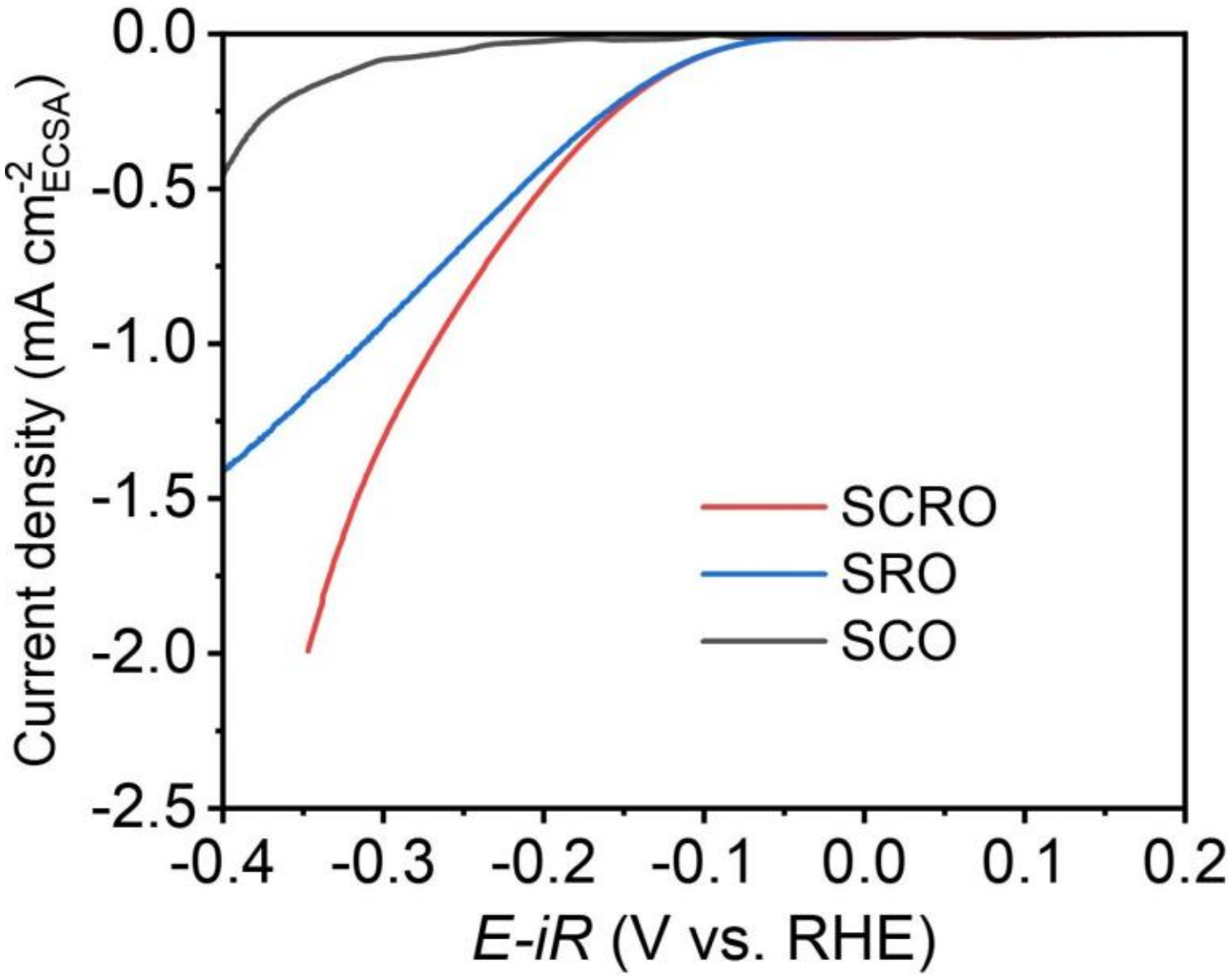

Supplementary Figure 18. Specific activity for HER normalized to ECSA of SCRO, SCO and SRO as a function of applied potentials.

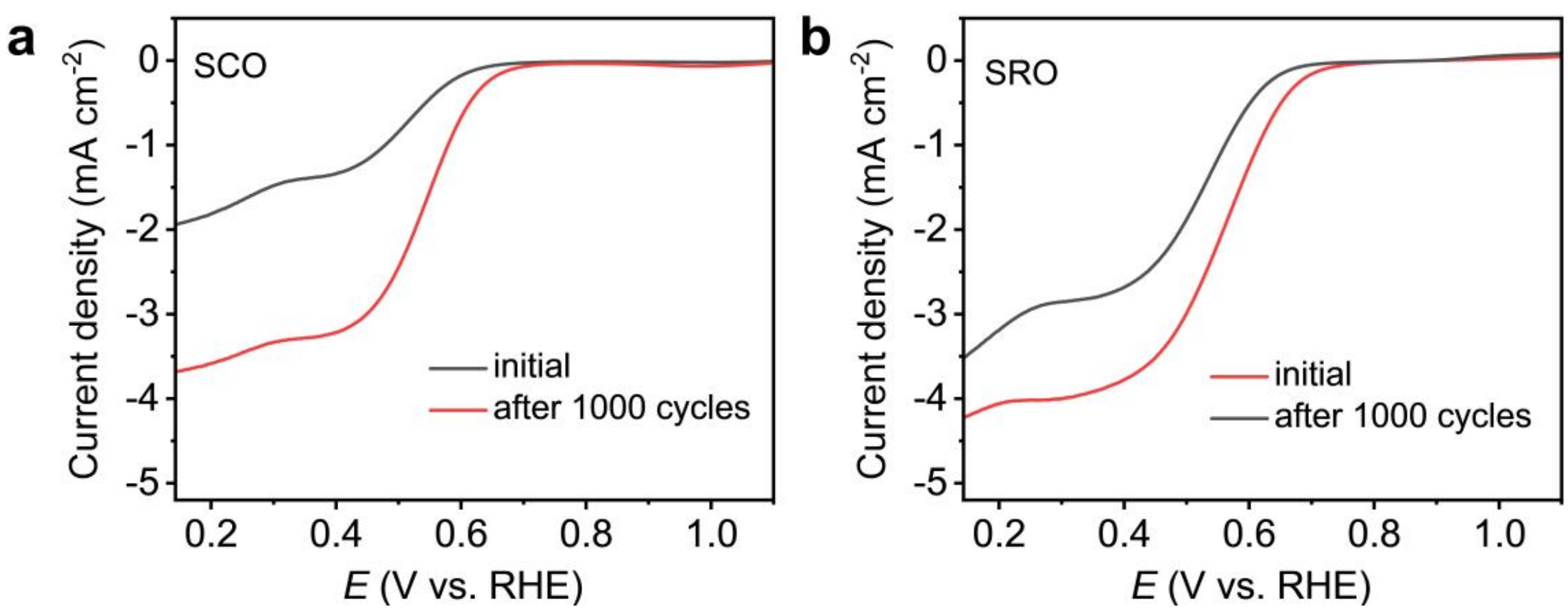

Supplementary Figure 19. Polarization curves of (a) SCO and (b) SRO initially and after 1000 cycles during accelerated durability tests.

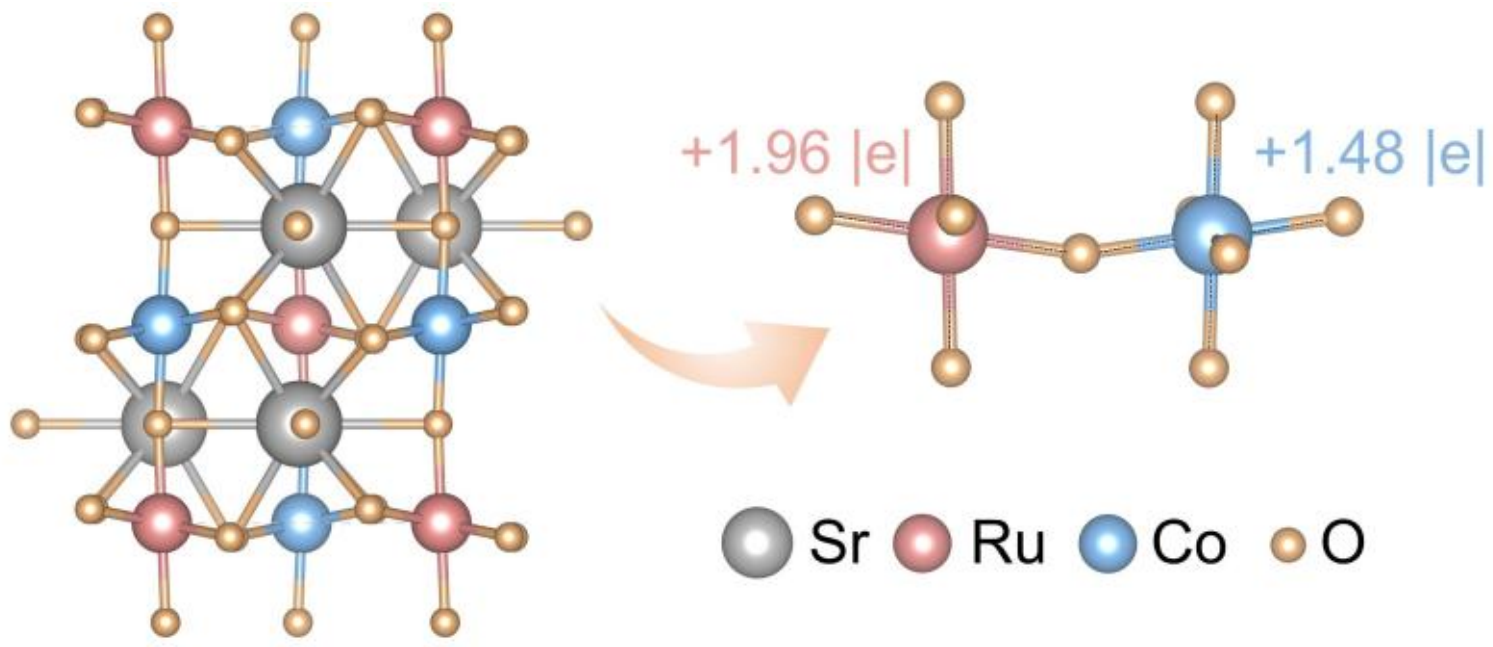

Supplementary Figure 20. Structural analysis and Bader charges at different sites of Co and Ru in SCRO calculated from the DFT calculations.

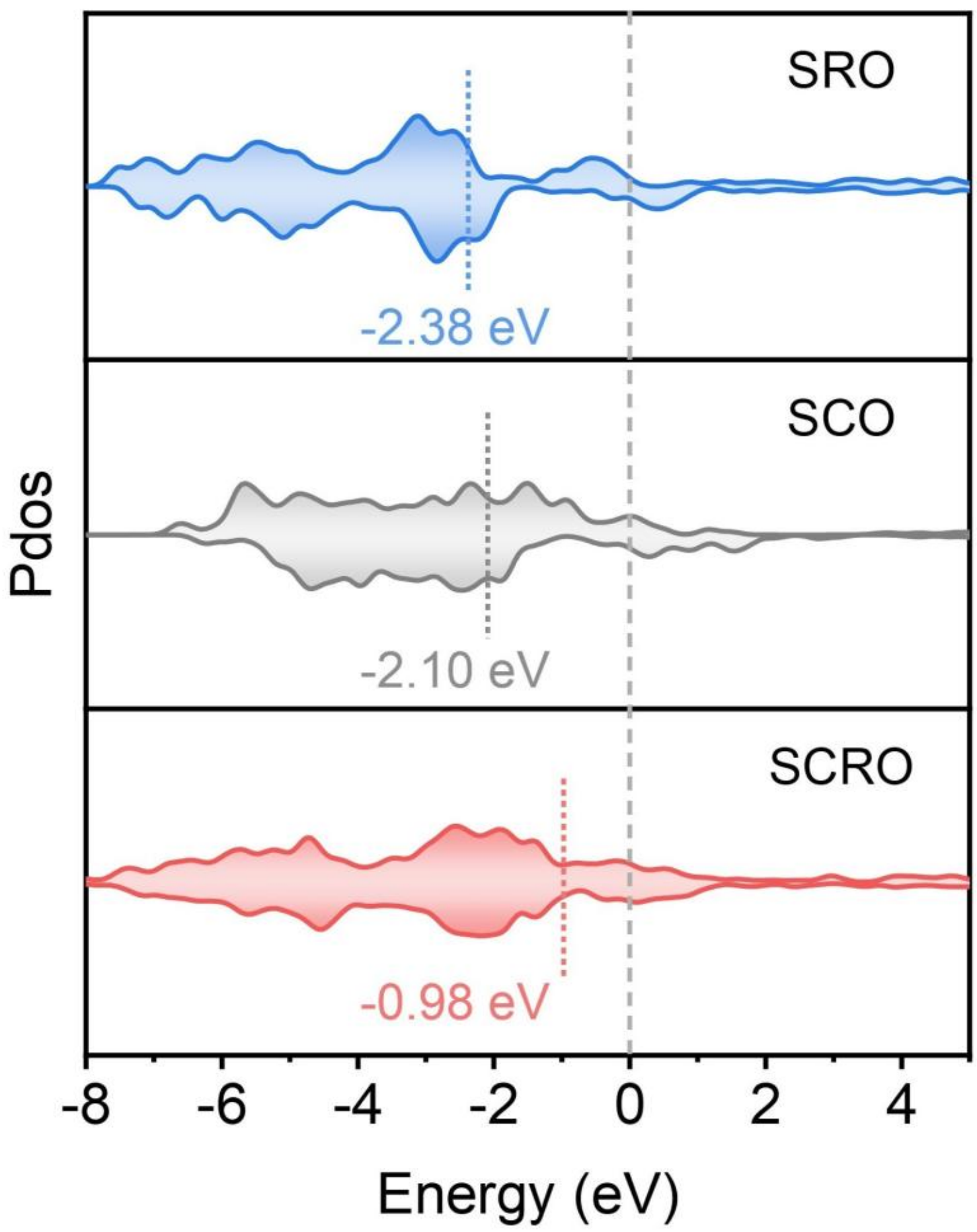

Supplementary Figure 21. PDOS of O 2*p* orbitals for SCRO, SRO, and SCO.

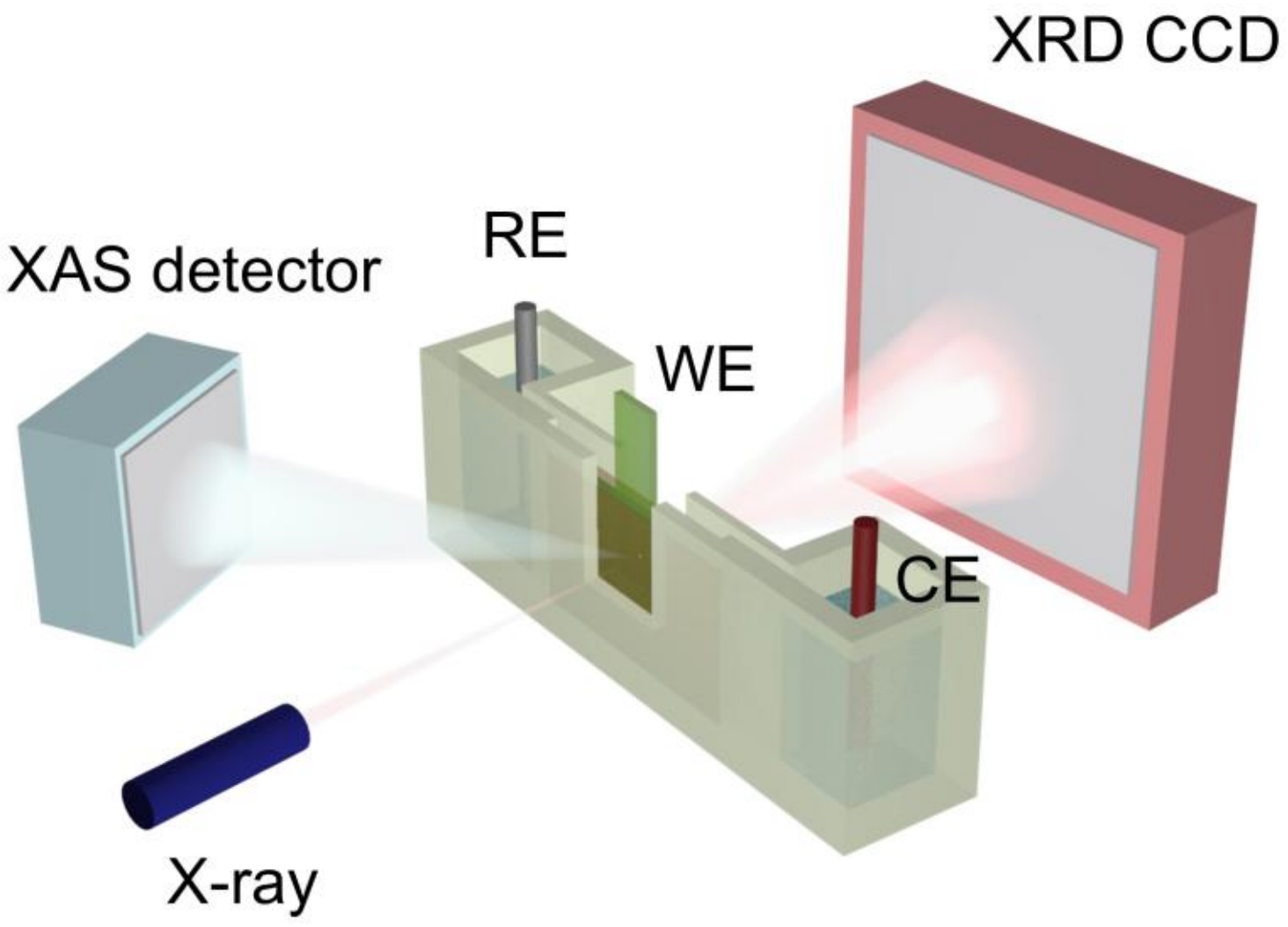

Supplementary Figure 22. Schematic illustration of the custom-built electrochemical set-ups for *in-situ* XAS and XRD measurements.

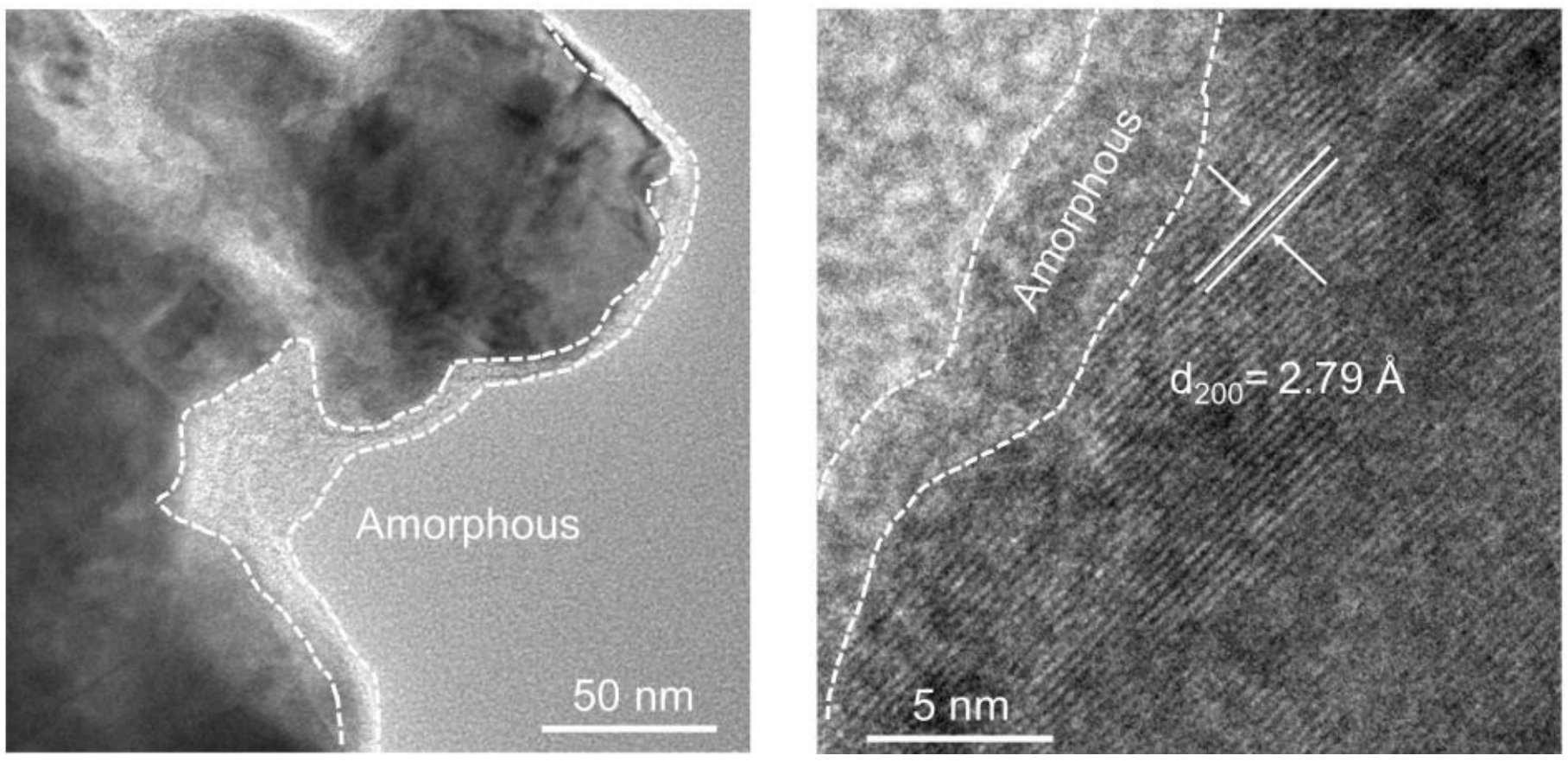

Supplementary Figure 23. HRTEM images of SCMO after OER at other zone.

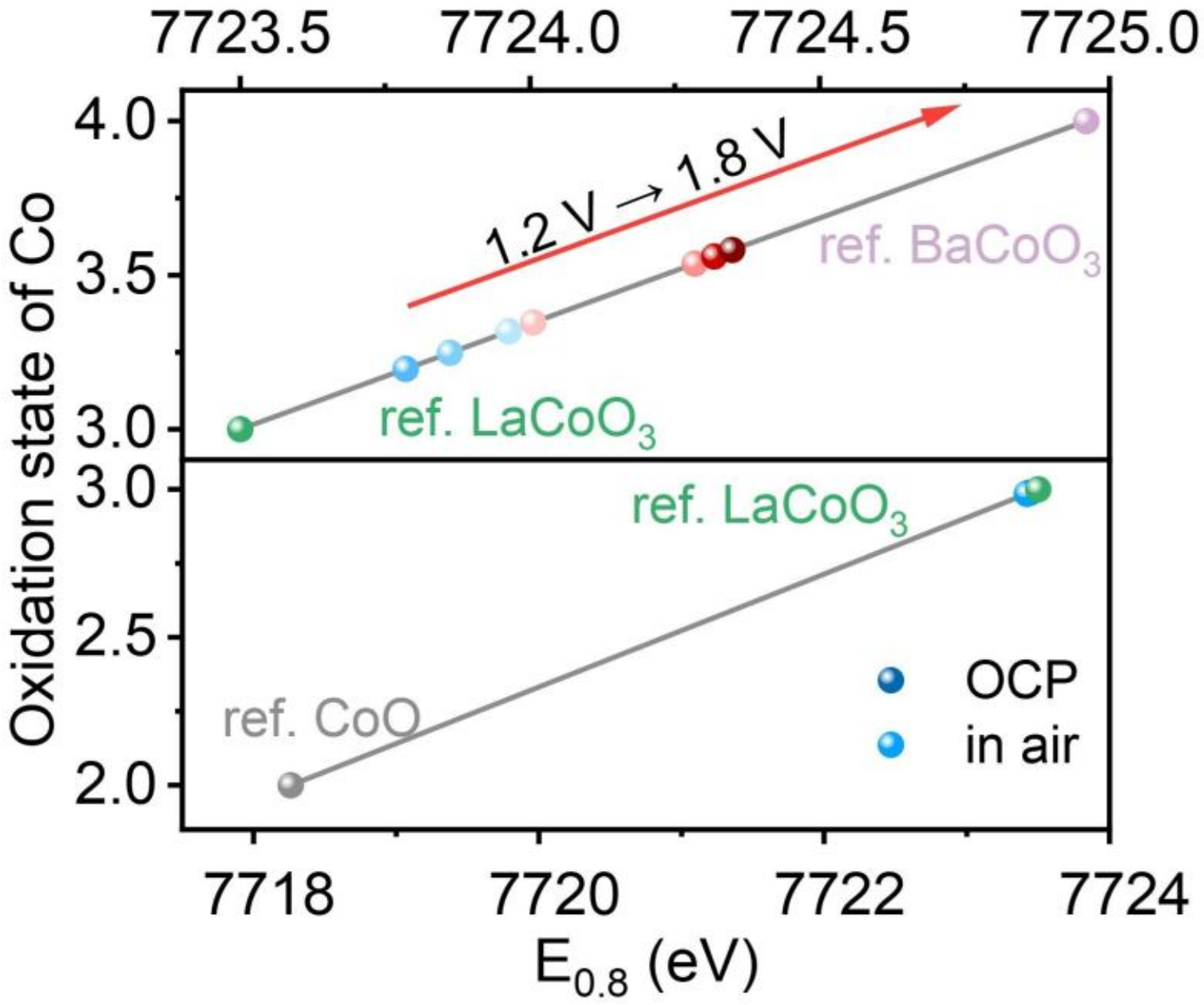

Supplementary Figure 24. Oxidation states of Co during OER derived from the normalized XANES spectra at the Co *K*-edge.

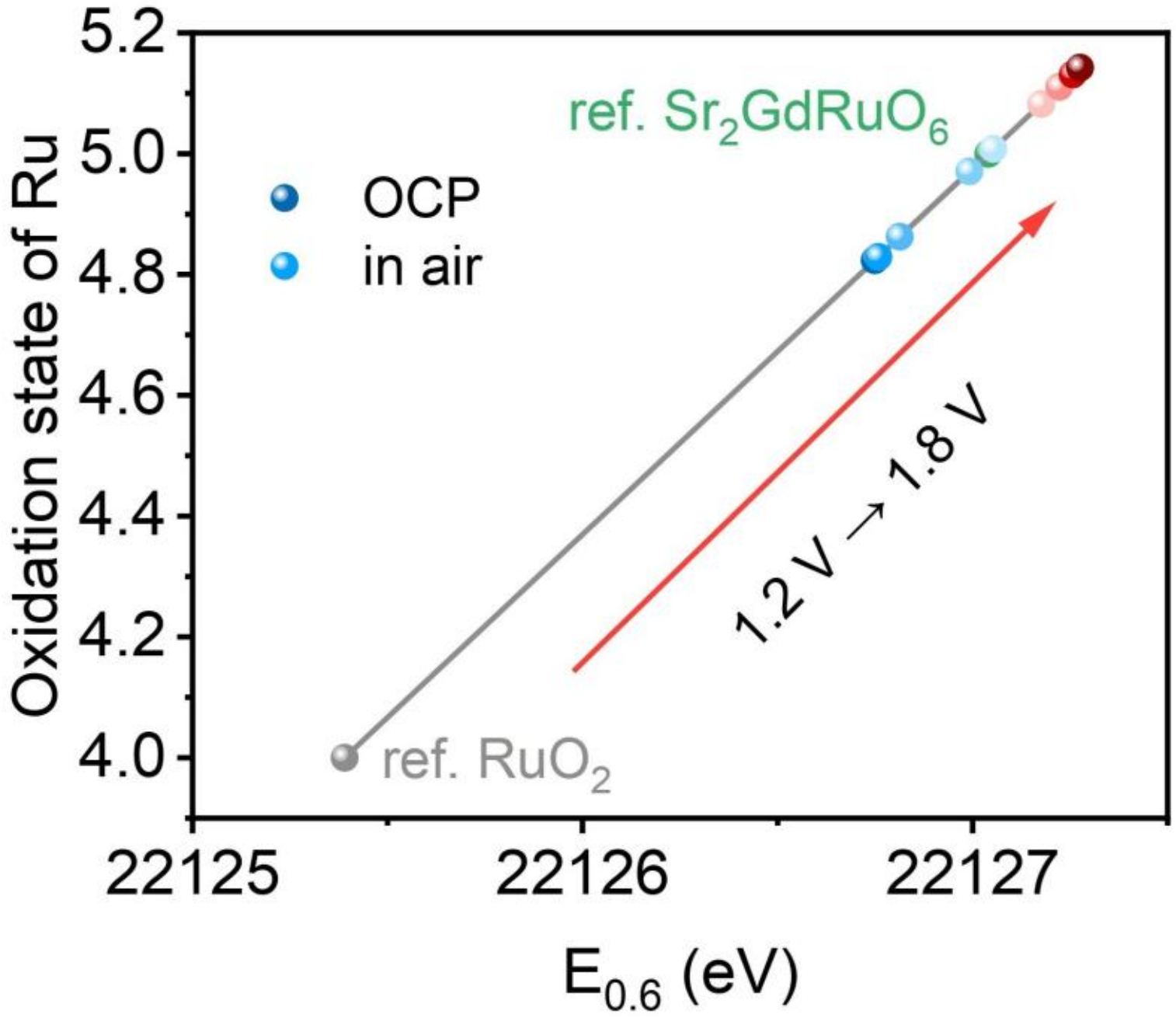

Supplementary Figure 25. Oxidation states of Ru during OER derived from the normalized XANES spectra at the Ru *K*-edge.

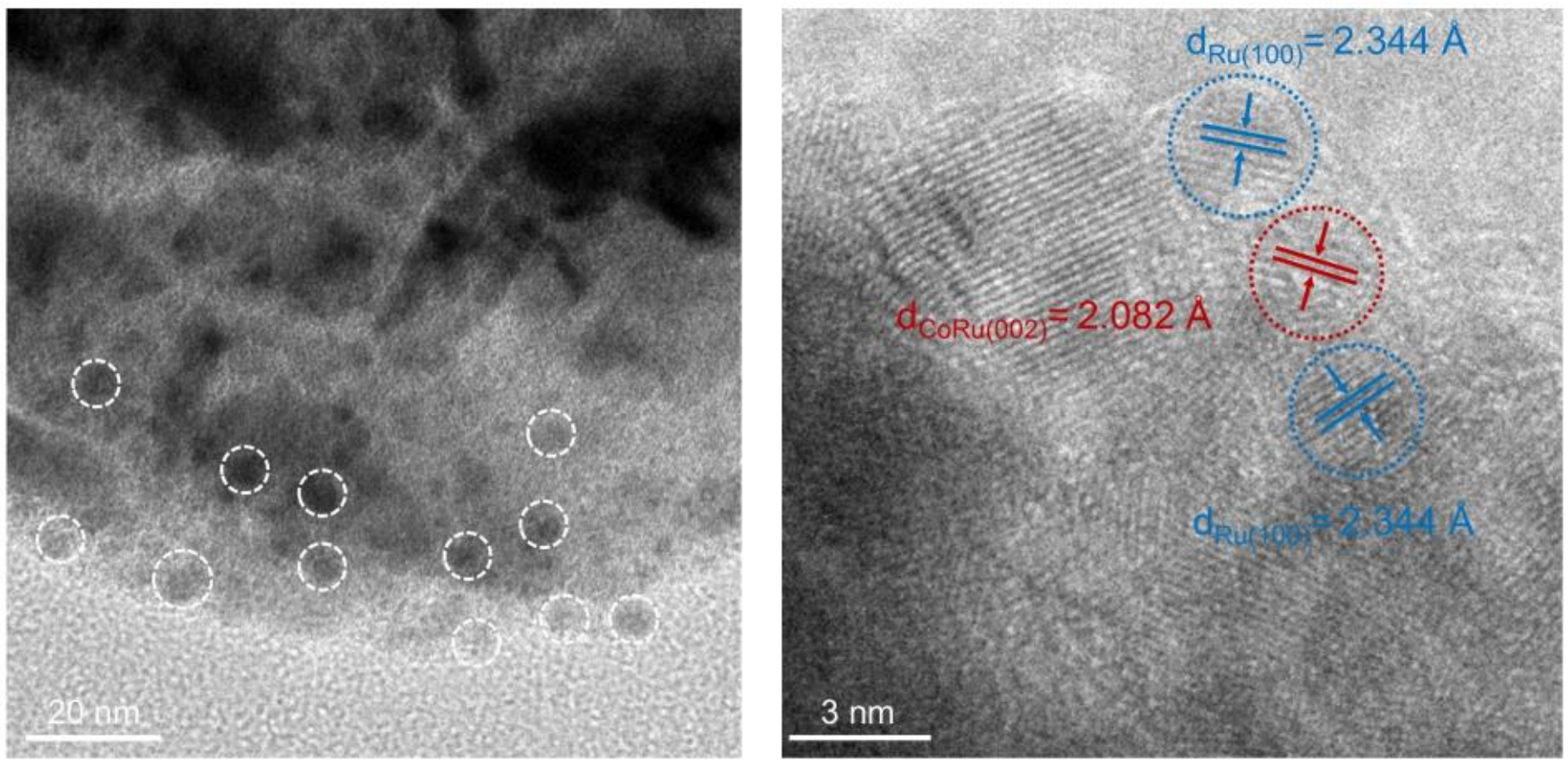

Supplementary Figure 26. HRTEM images of SCMO after HER at other zone.

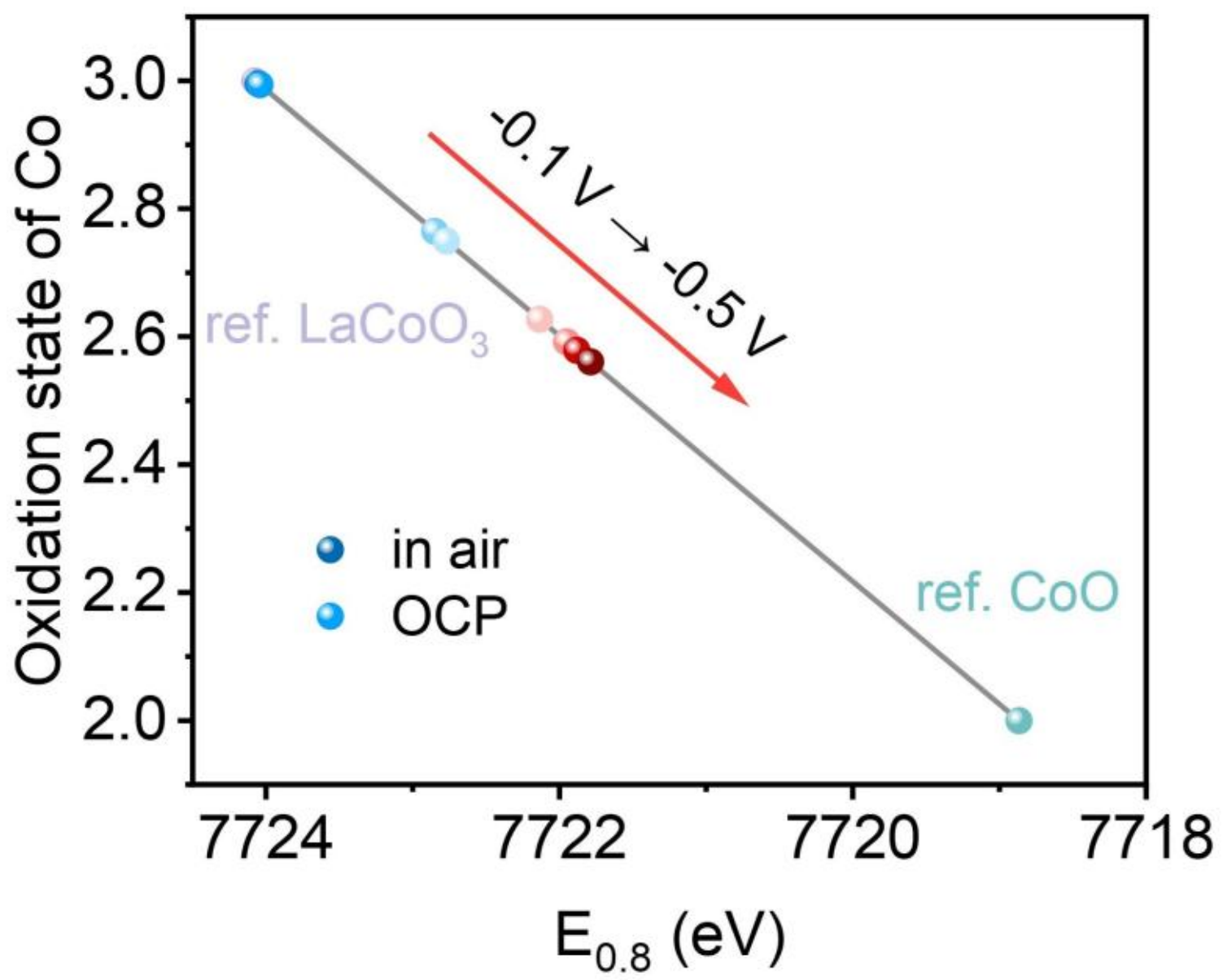

Supplementary Figure 27. Oxidation states of Co during HER derived from the normalized XANES spectra at the Co *K*-edge.

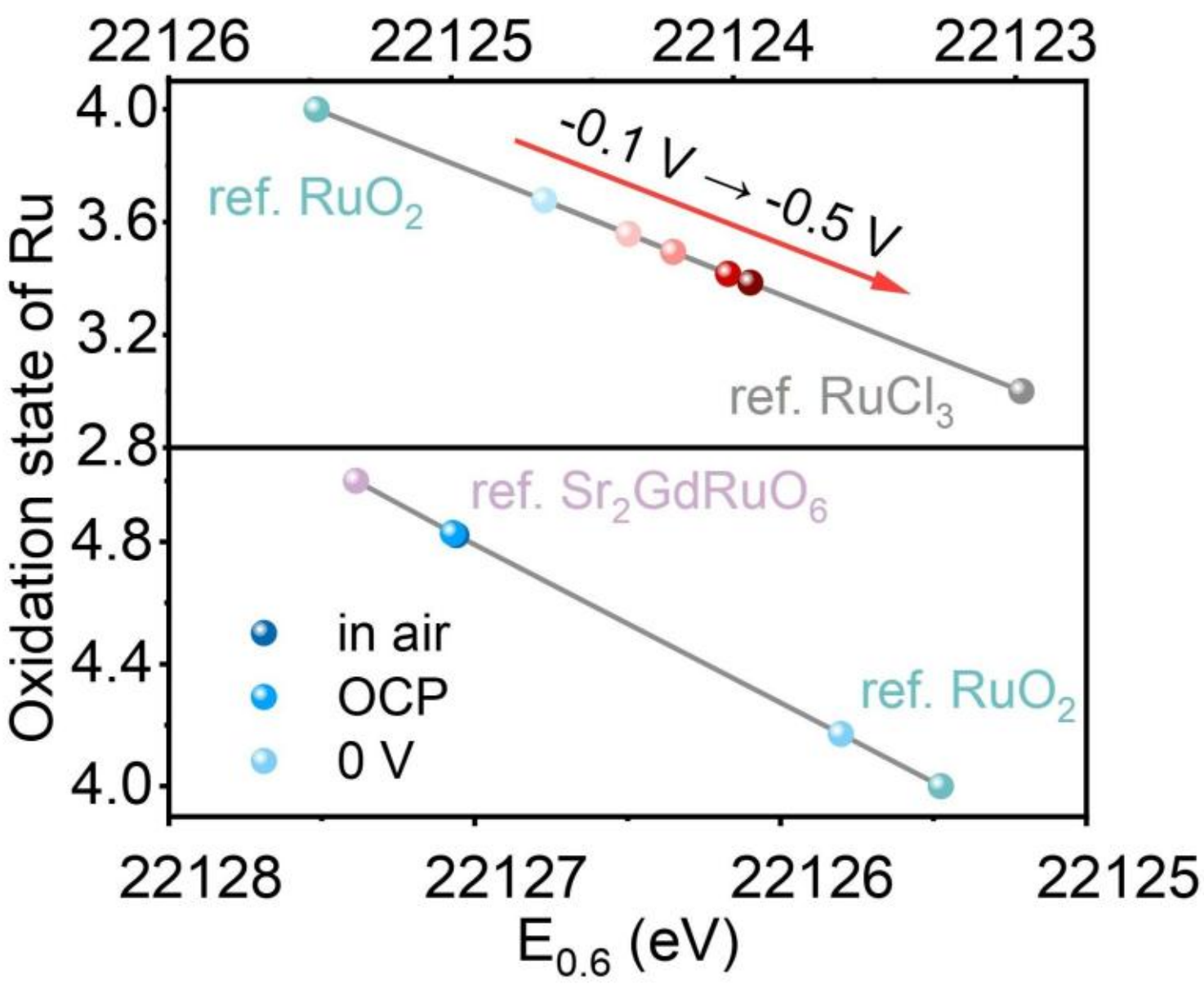

Supplementary Figure 28. Oxidation states of Ru during HER derived from the normalized XANES spectra at the Ru *K*-edge.

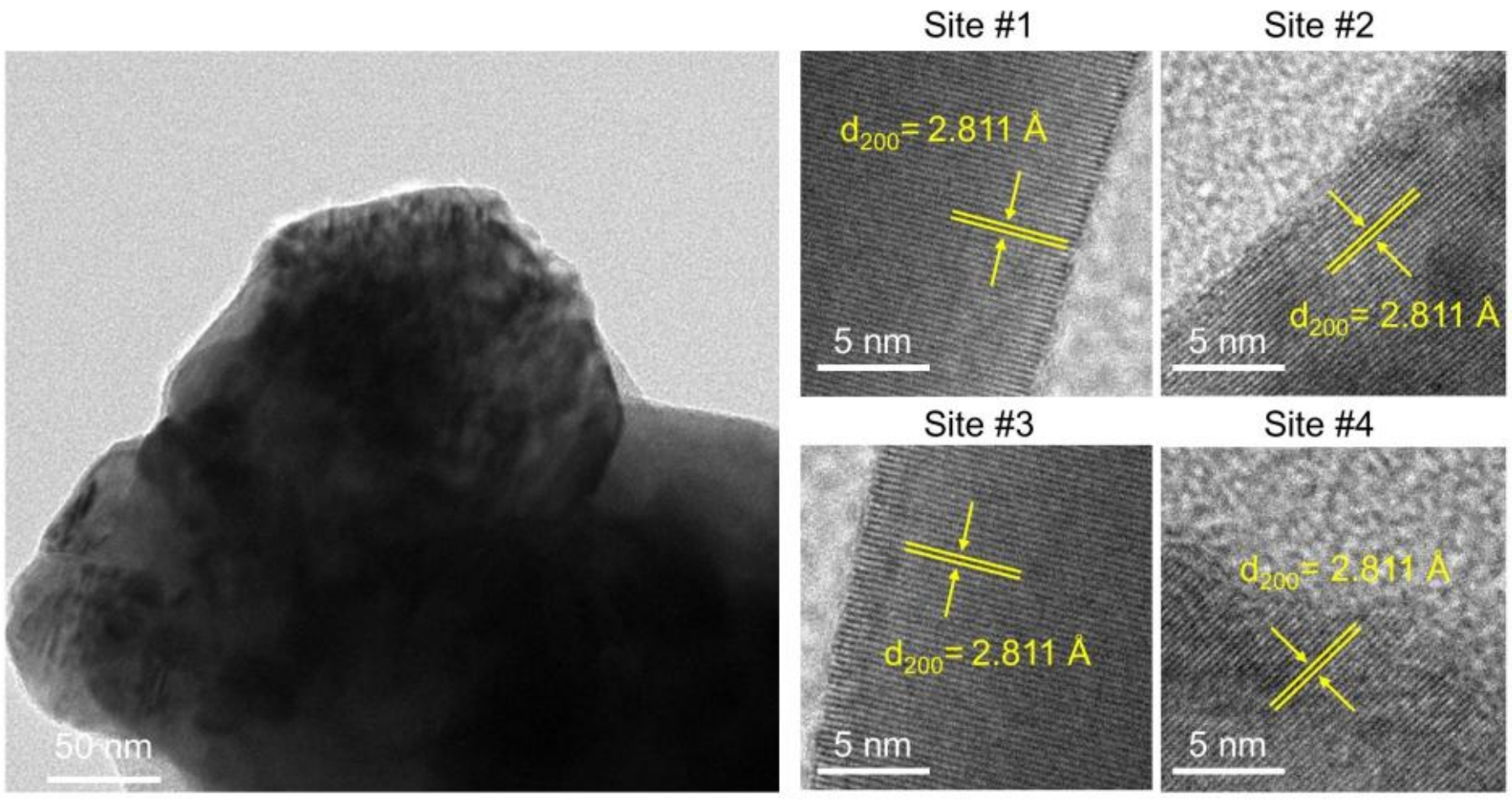

Supplementary Figure 29. HRTEM images of SCMO after ORR at other zone.

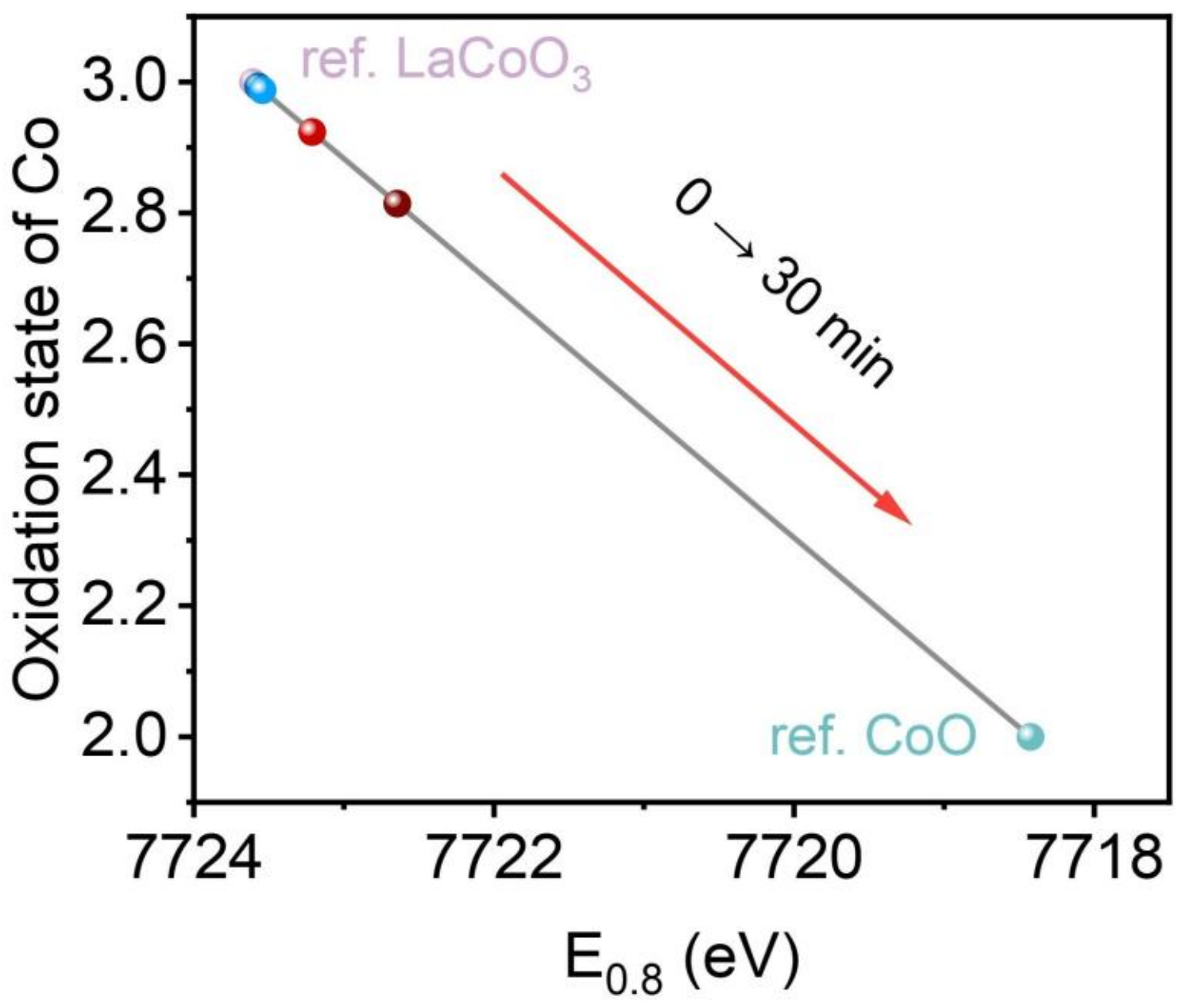

Supplementary Figure 30. Oxidation states of Co during ORR derived from the normalized XANES spectra at the Co *K*-edge.

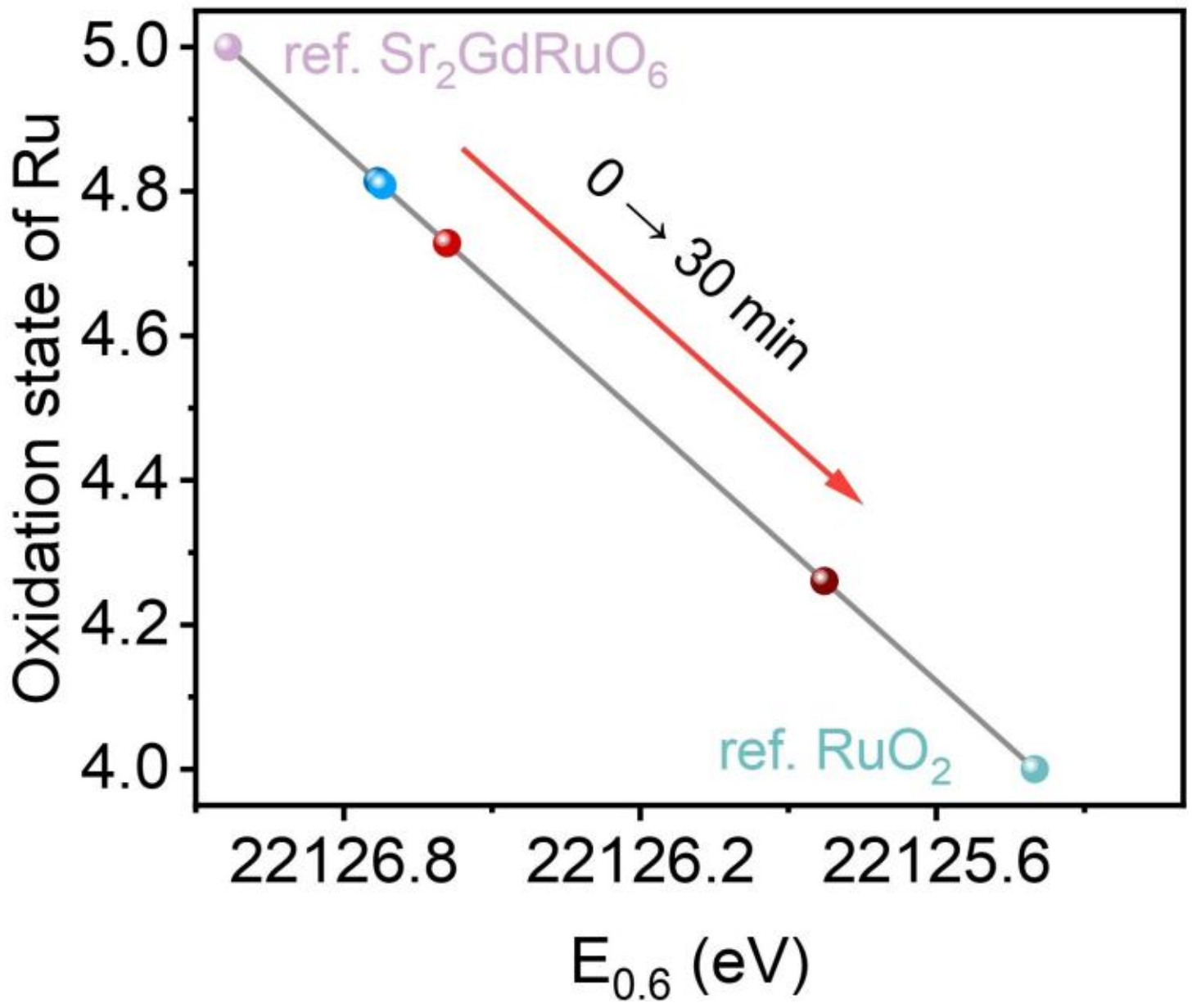

Supplementary Figure 31. Oxidation states of Ru during ORR derived from the normalized XANES spectra at the Ru *K*-edge.

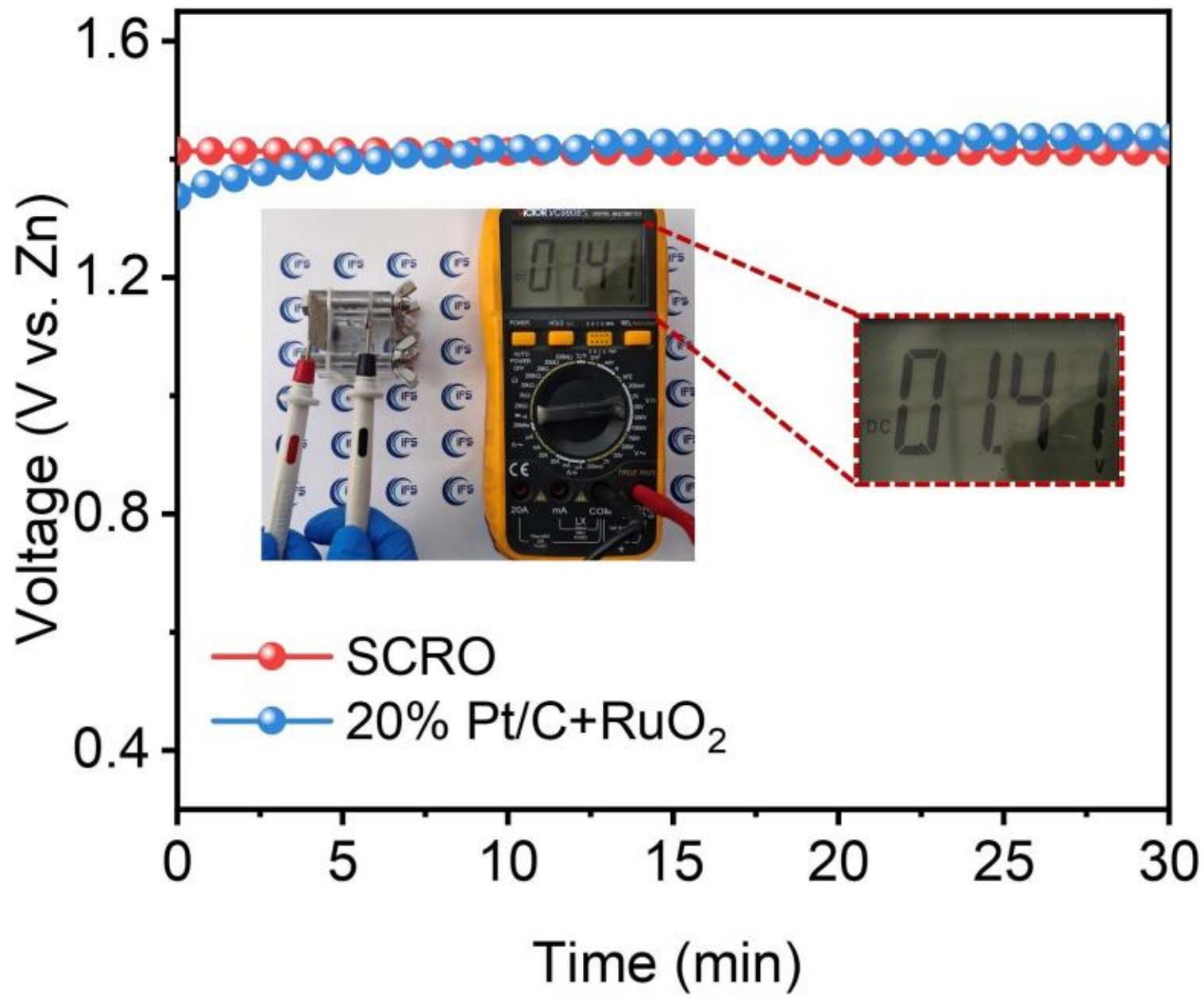

Supplementary Figure 32. Open circuit voltage of the liquid ZABs based on the SCRO and benchmark 20% Pt/C+RuO_2.

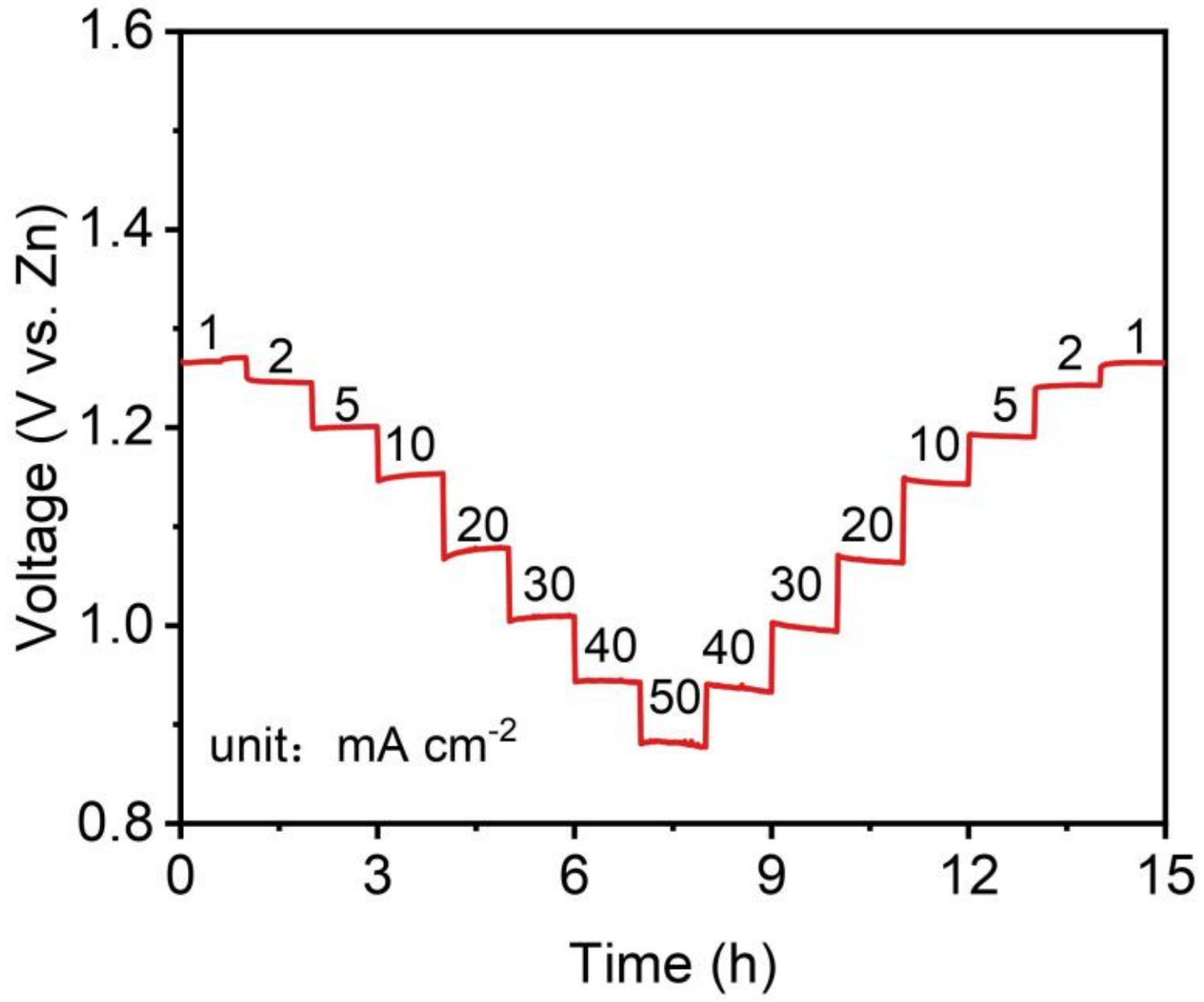

Supplementary Figure 33. Galvanostatic discharge curves at different current densities (1, 2, 5, 10, 20, 30, 40, 50 mA cm^{-2}).

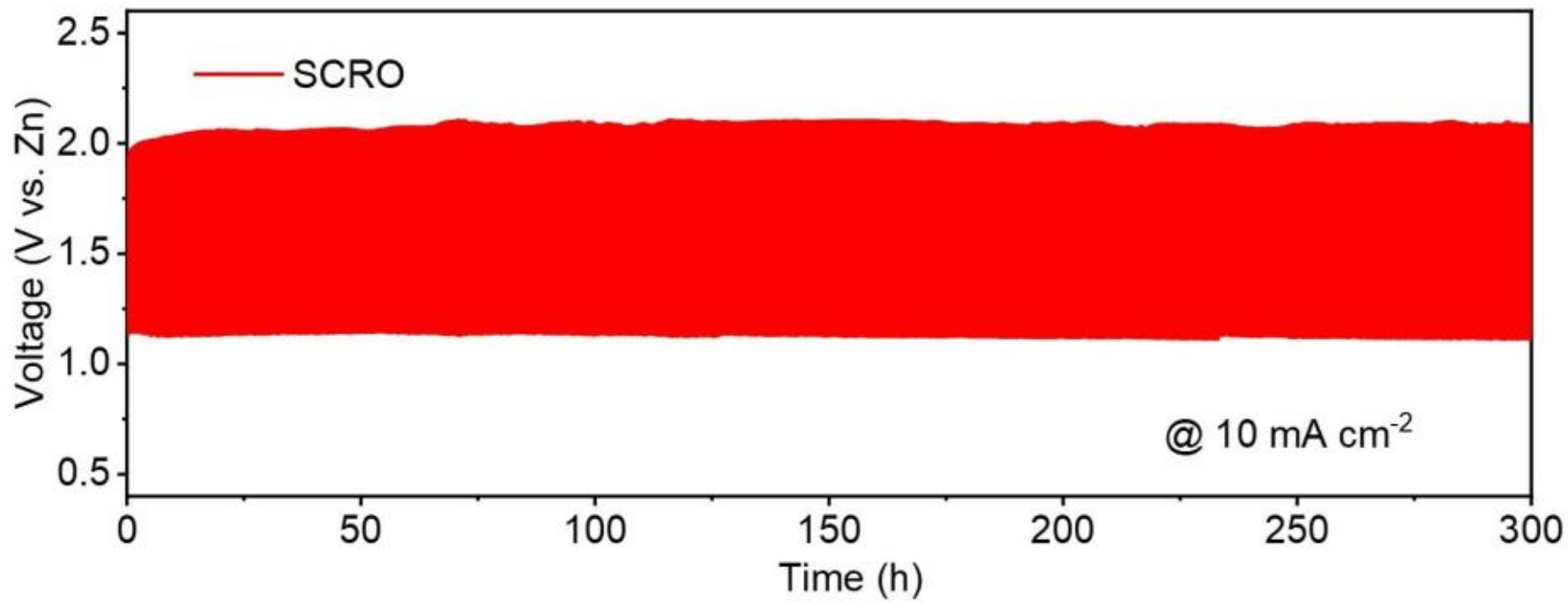

Supplementary Figure 34. Charge-discharge cycling curves at 10 mA cm^{-2} of SCRO-based liquid ZAB.

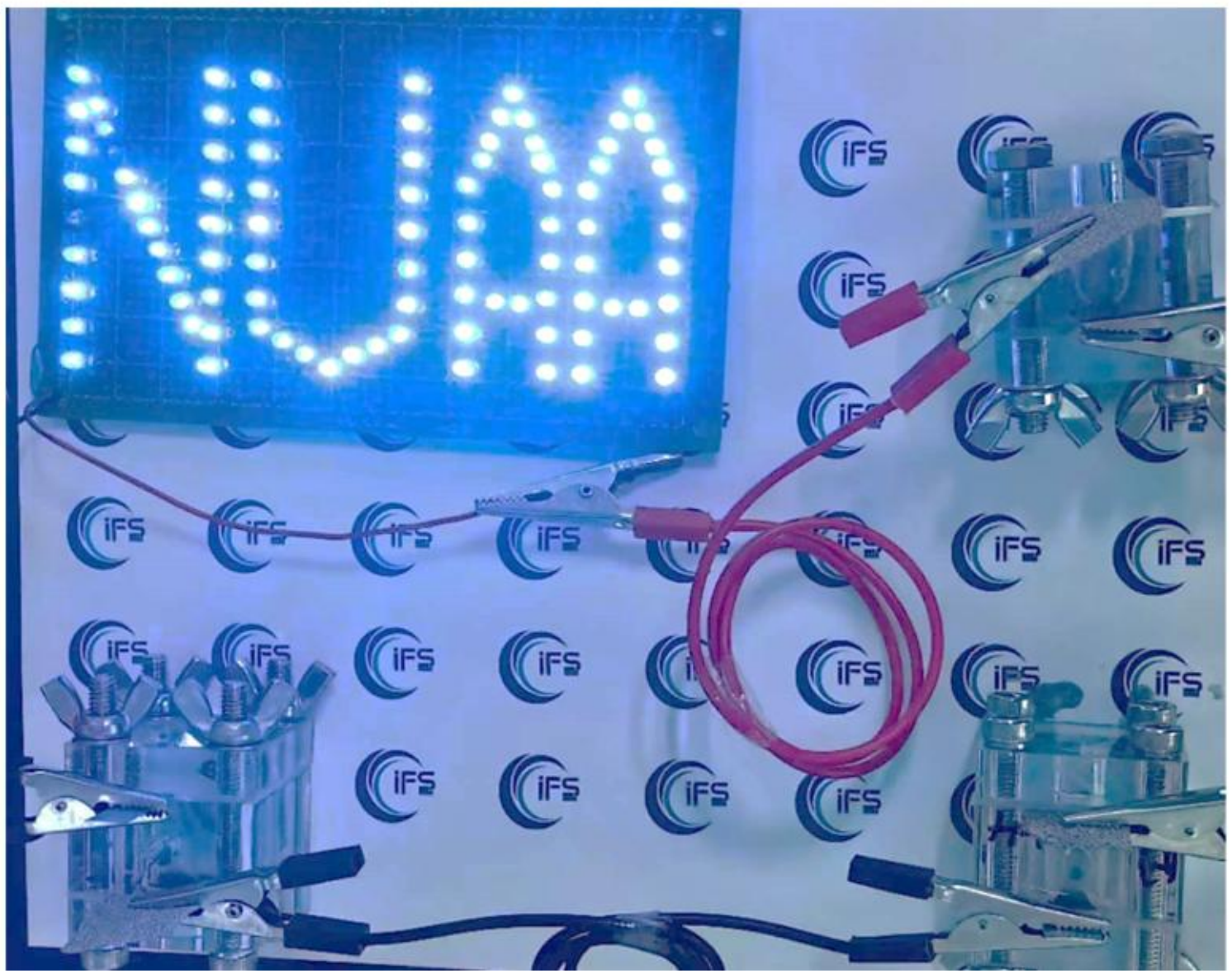

Supplementary Figure 35. Photograph of an indicator light LED screen showing the "NUAA" powered by three liquid Zn-air batteries with SCRO air-cathode connected in series.

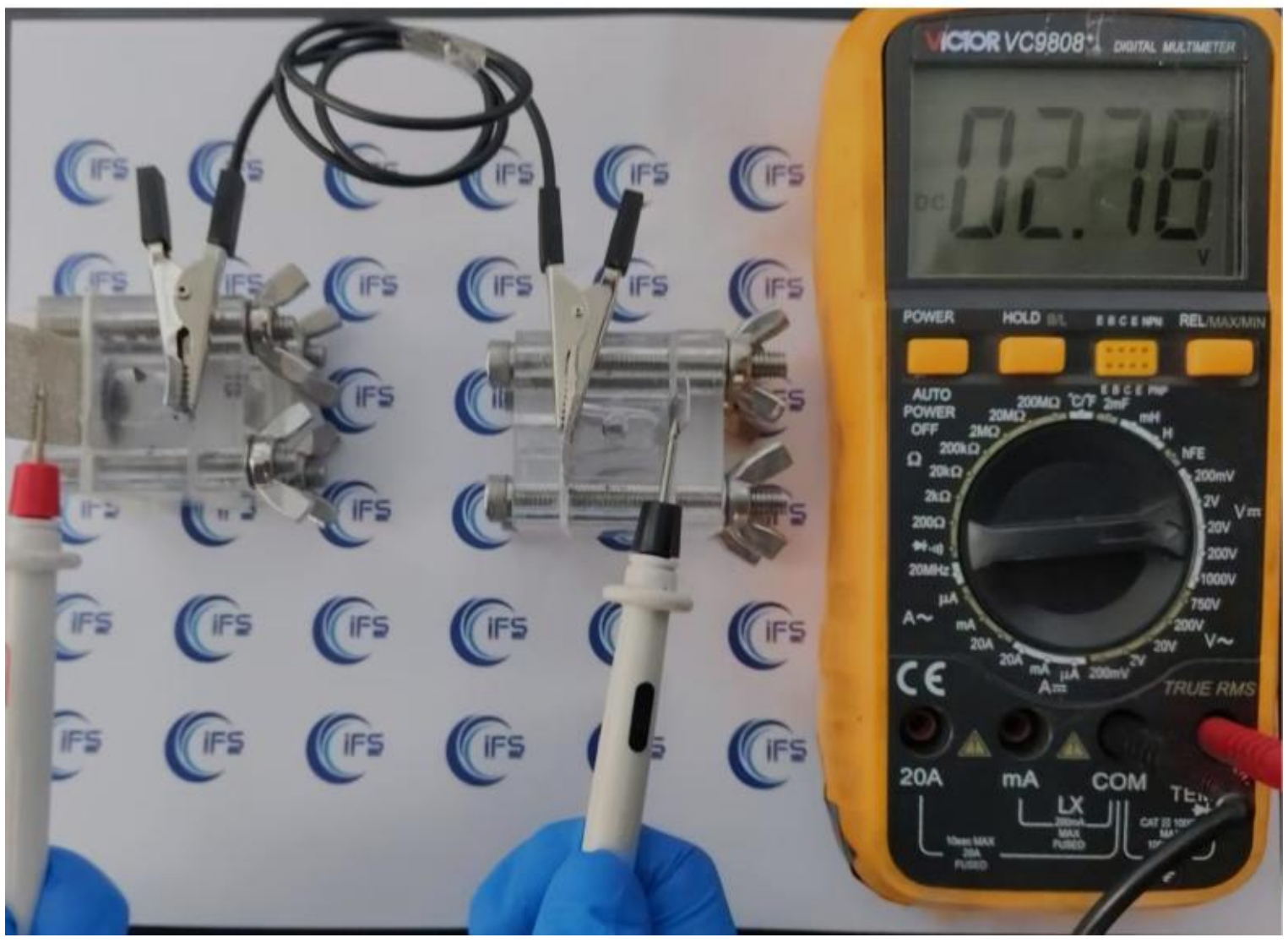

Supplementary Figure 36. Digital image of two ZABs connected in series showing working voltage of 2.78 V.

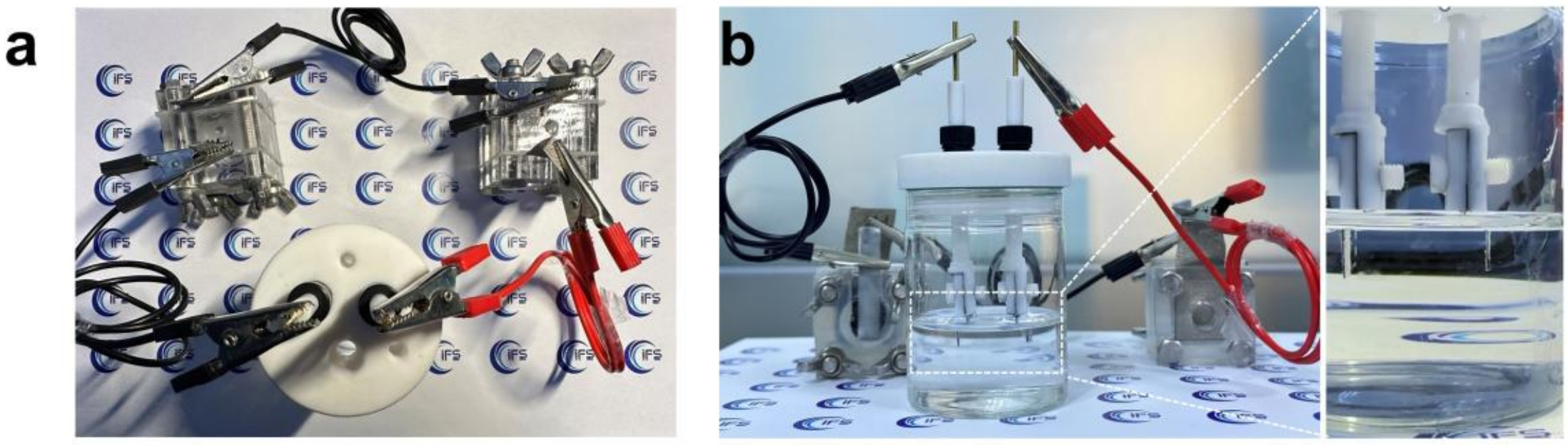

Supplementary Figure 37. (a) A photograph showing two-electrode water splitting device powered by two ZABs. (b) The electrocatalytic evolution of hydrogen (left) and oxygen (right) bubbles.

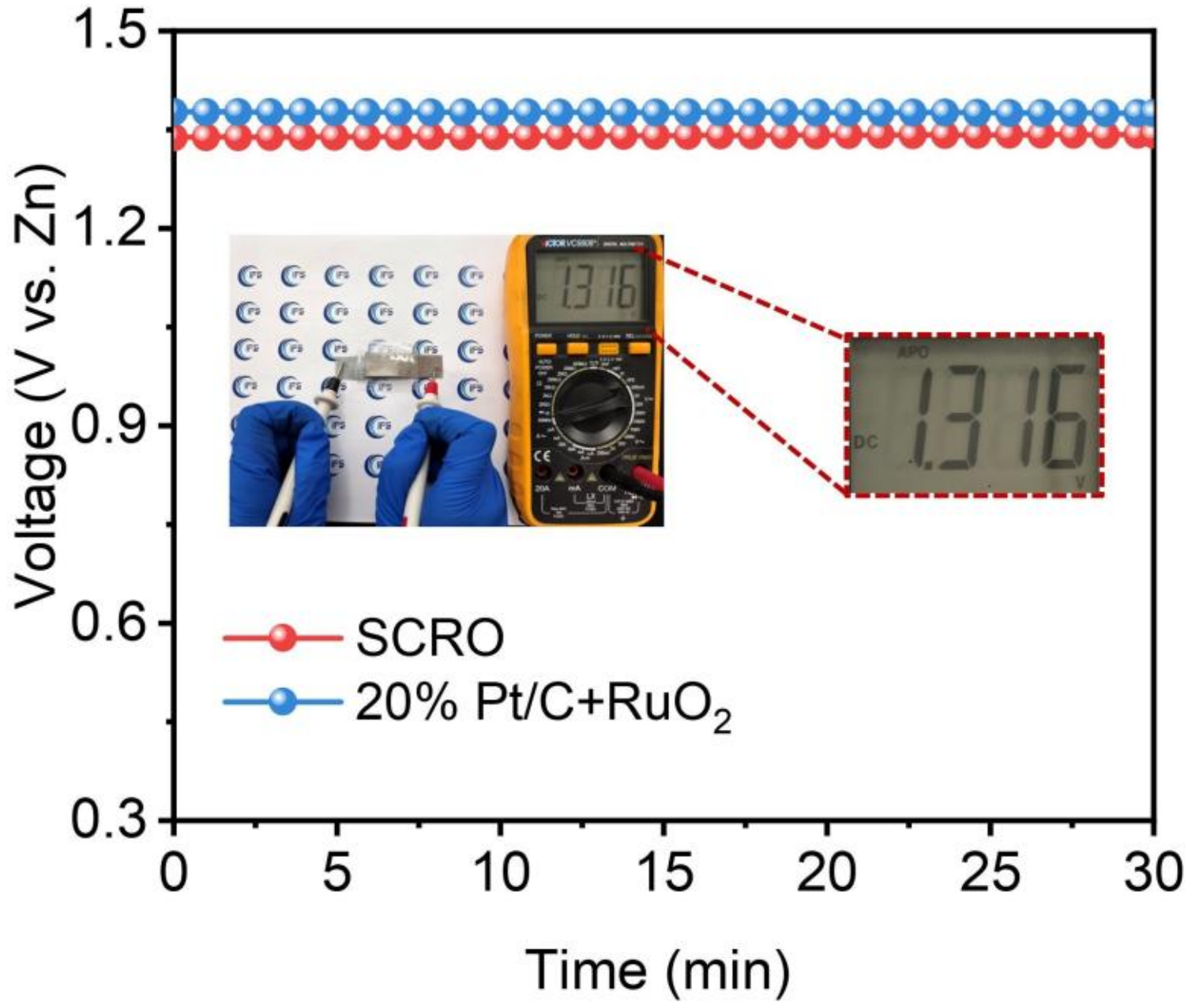

Supplementary Figure 38. Open-circuit voltage plots of flexible ZABs based on the SCRO and benchmark 20% Pt/C+RuO_2.

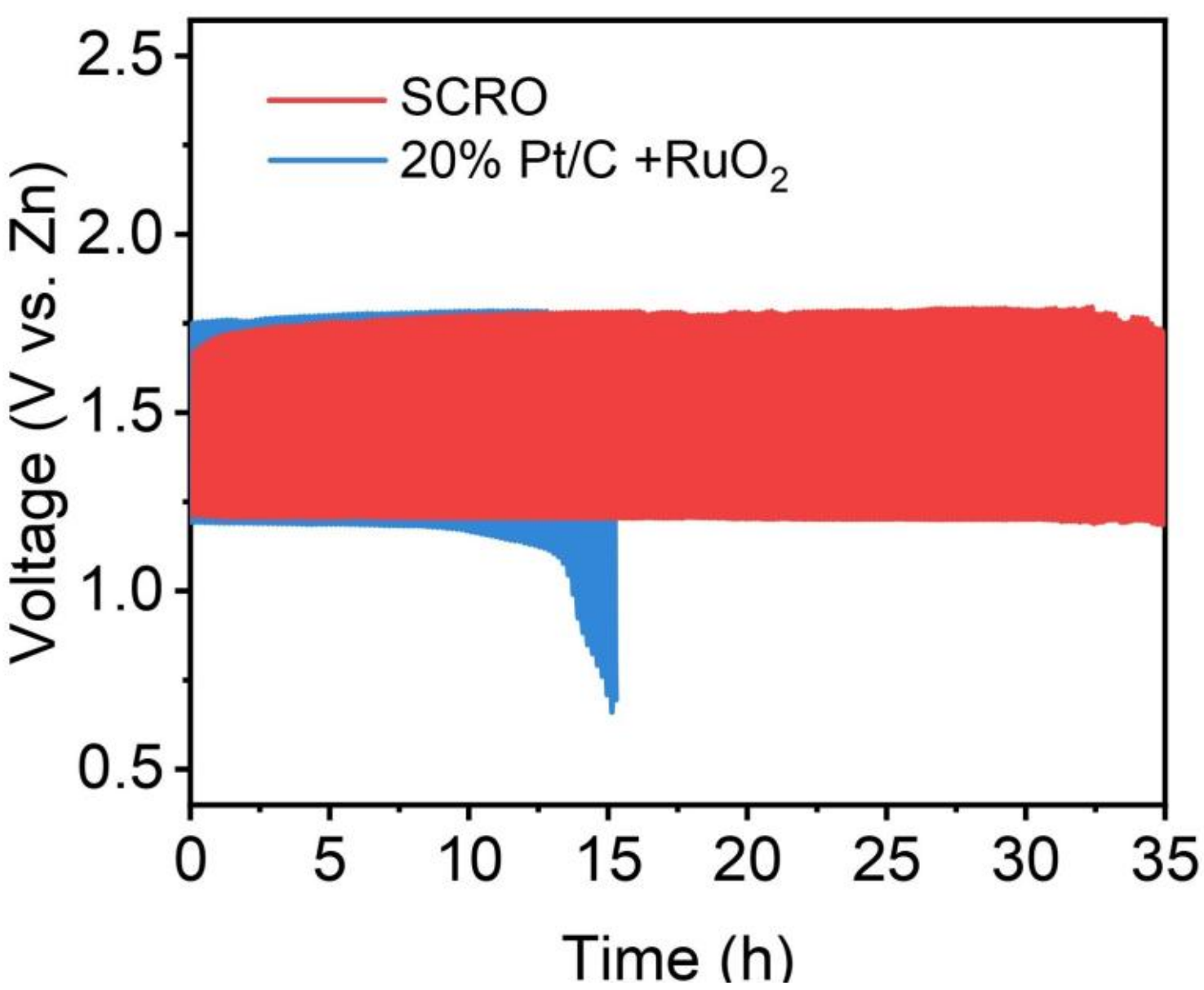

Supplementary Figure 39. Charge-discharge cycling curves at the current density of 1 mA cm^{-2} (10 min each cycle) of flexible ZABs based on SCRO and 20% Pt/C+RuO_2.

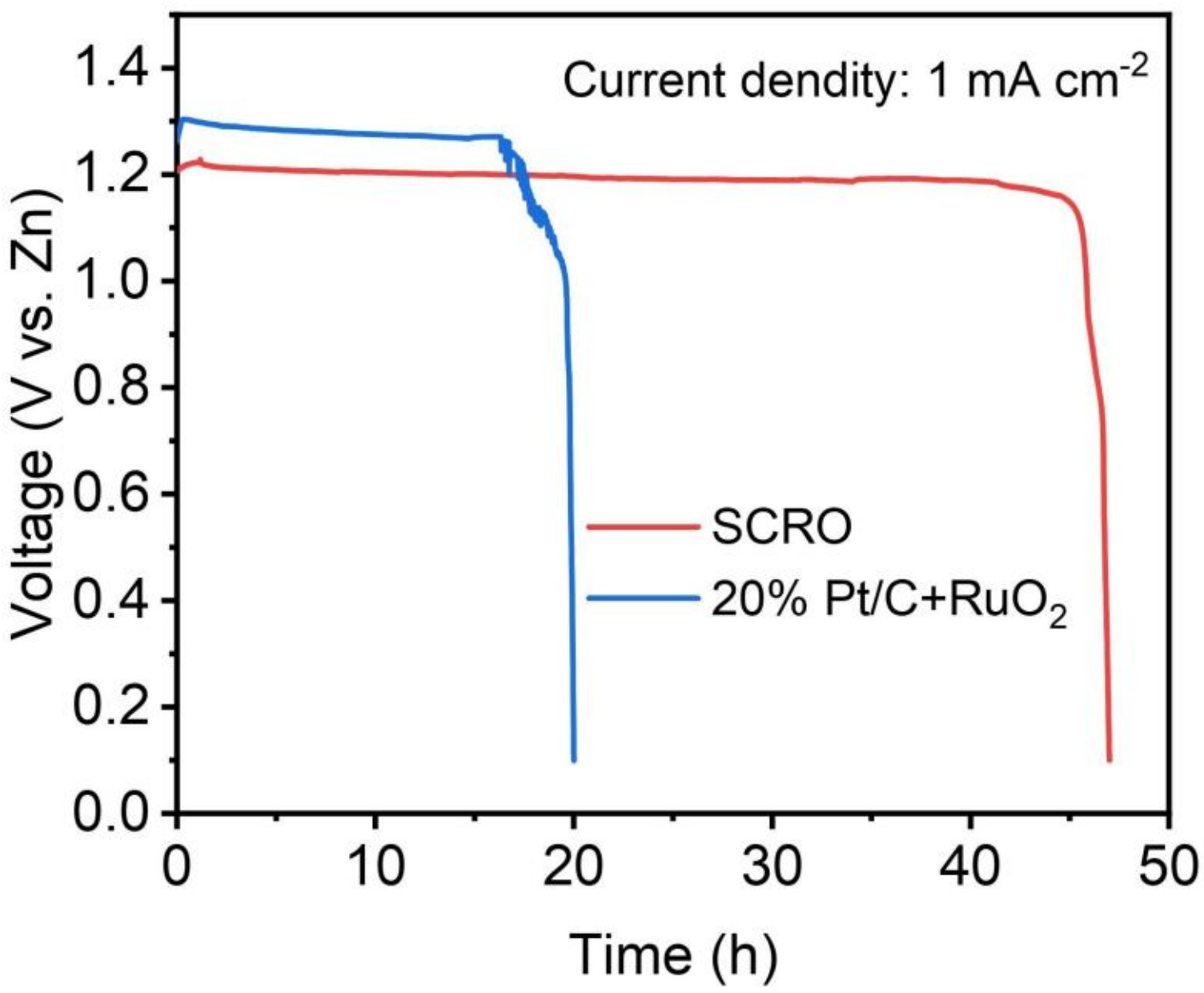

Supplementary Figure 40. Long-time galvanostatic discharging curves at 1 mA cm^{-2} of flexible ZABs based on SCRO and 20% Pt/C+RuO_2.

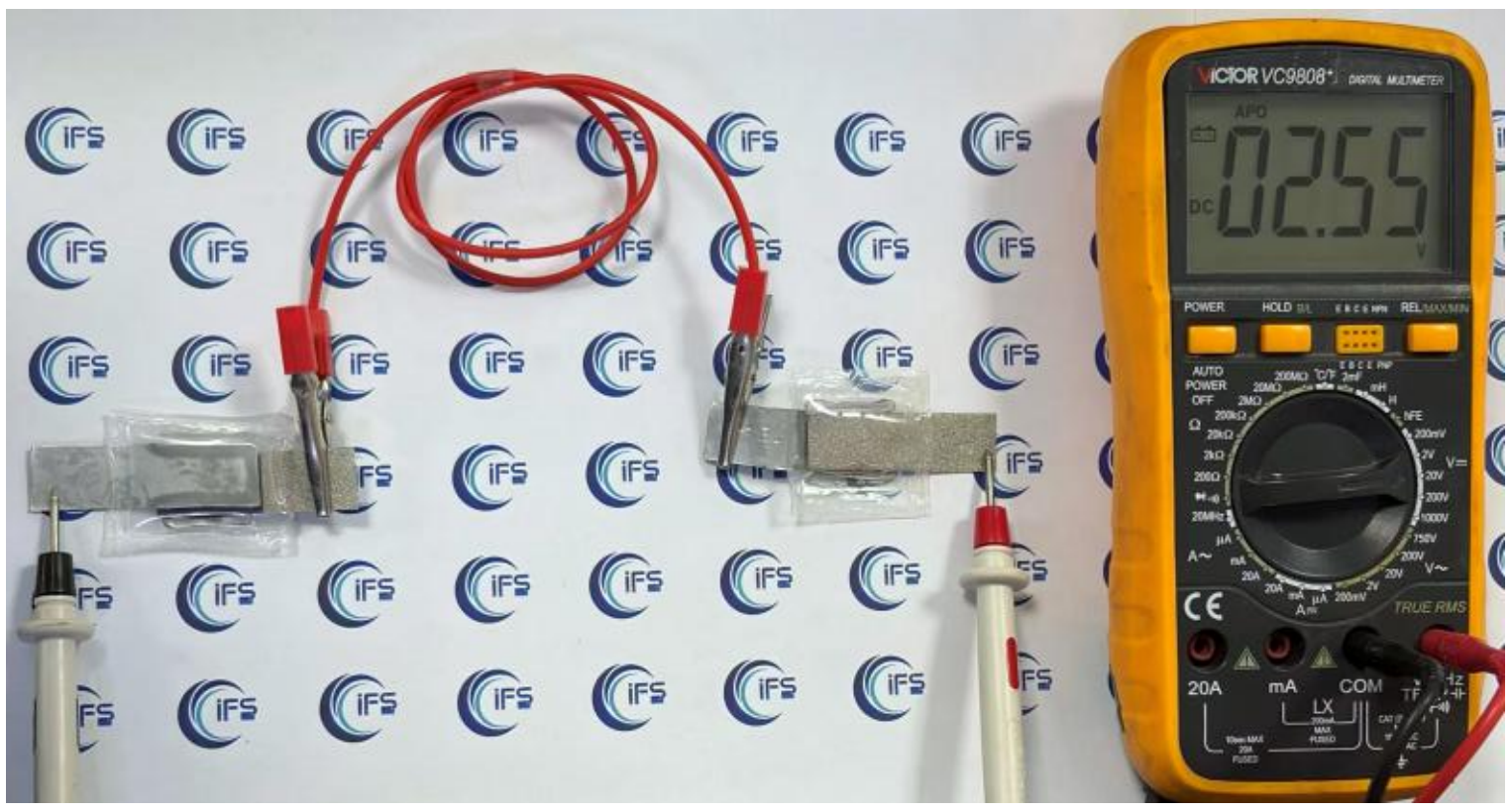

Supplementary Figure 41. Digital image of solid-state flexible ZAB connected in series showing working voltage of 2.55 V.

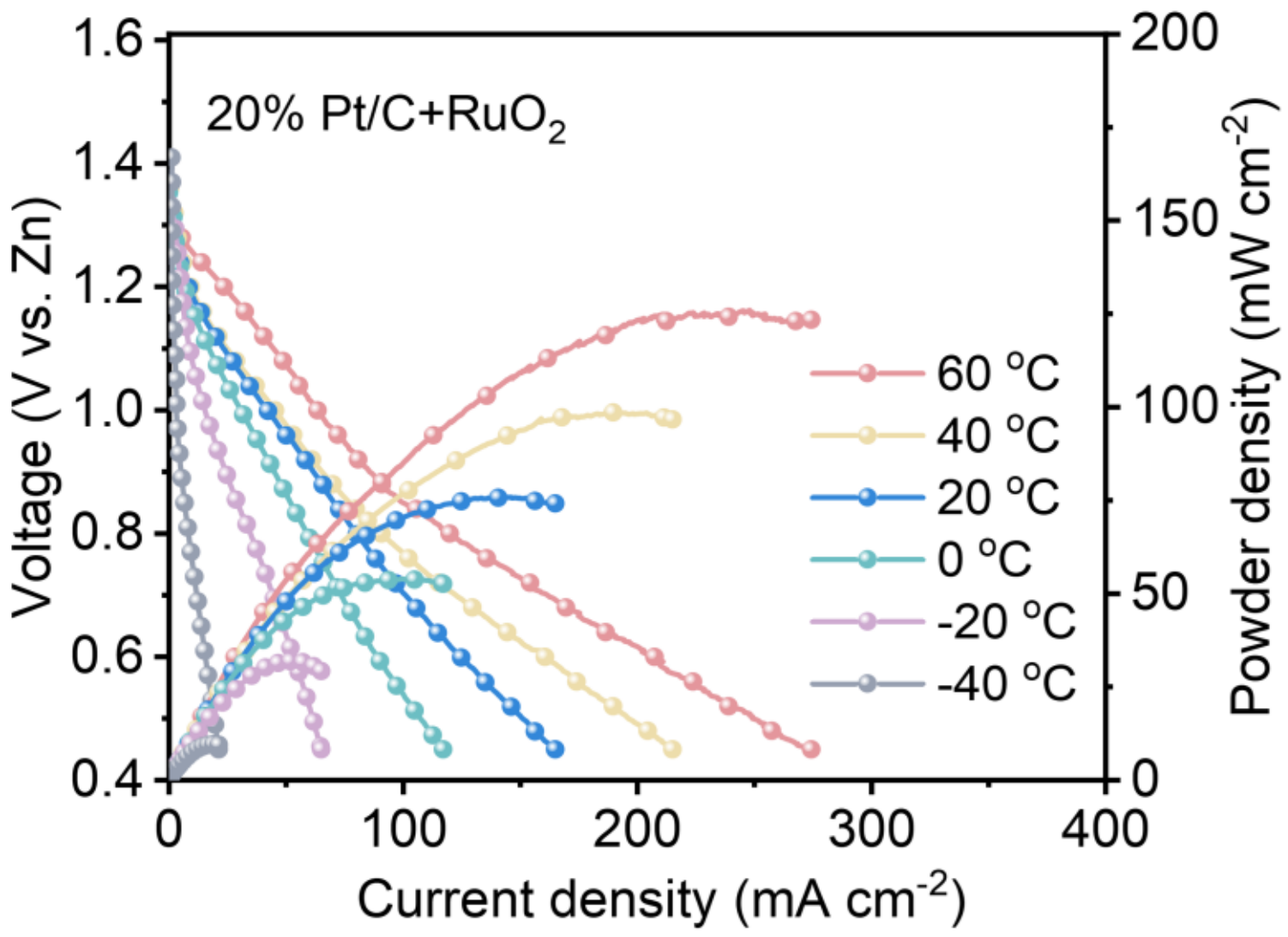

Supplementary Figure 42. Polarization curve and power density curves of 20% Pt/C+RuO_2-based ZABs from 60 to -40 ºC.

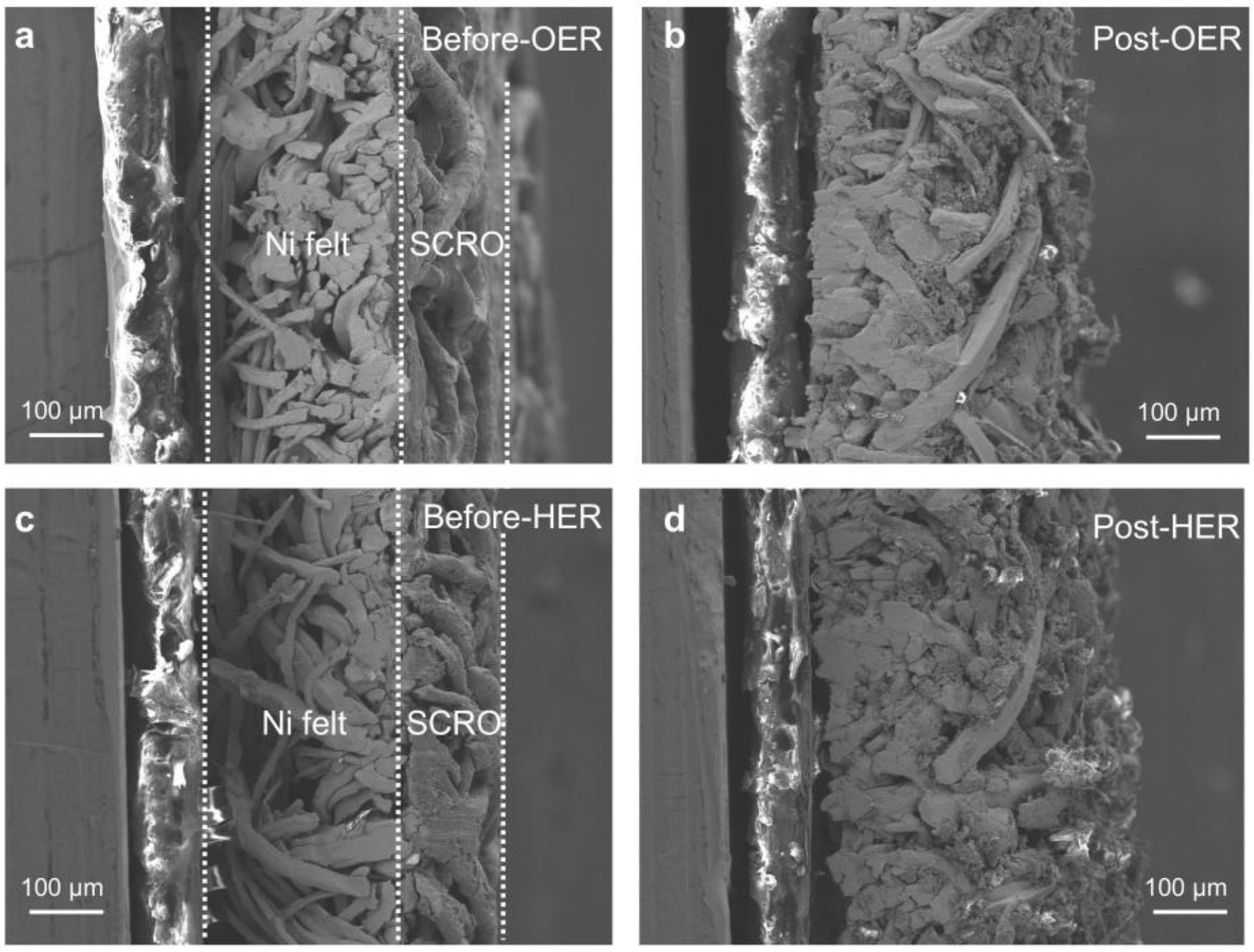

Supplementary Figure 43. Cross-sectional SEM images of the SCRO-coated Ni felt electrode before and after the AEMWE durability test: (a) pristine electrode before OER, (b) post-OER electrode, (c) pristine electrode before HER, and (d) post-HER electrode.

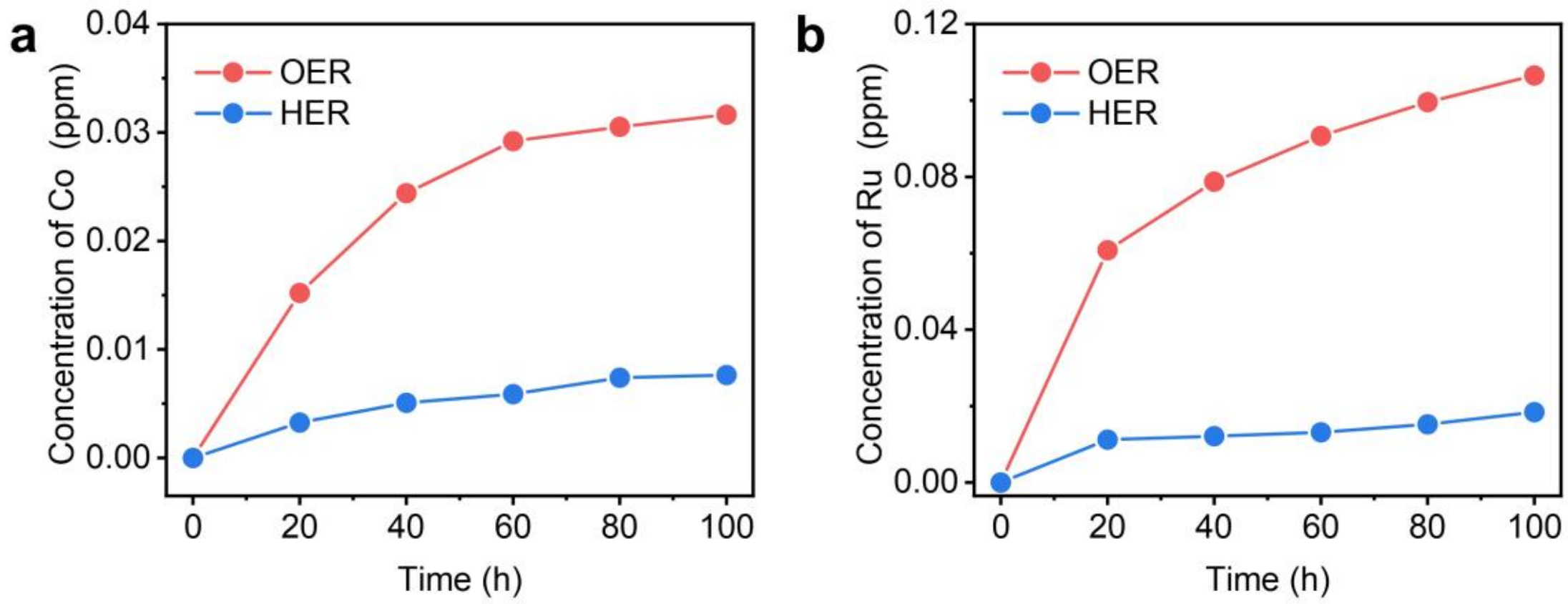

Supplementary Figure 44. Dissolution concentrations of (a) Co and (b) Ru ions in the electrolyte during OER and HER durability tests of the AEMWE at different time intervals of 20 h, 40 h, 60 h, 80 h and 100 h.

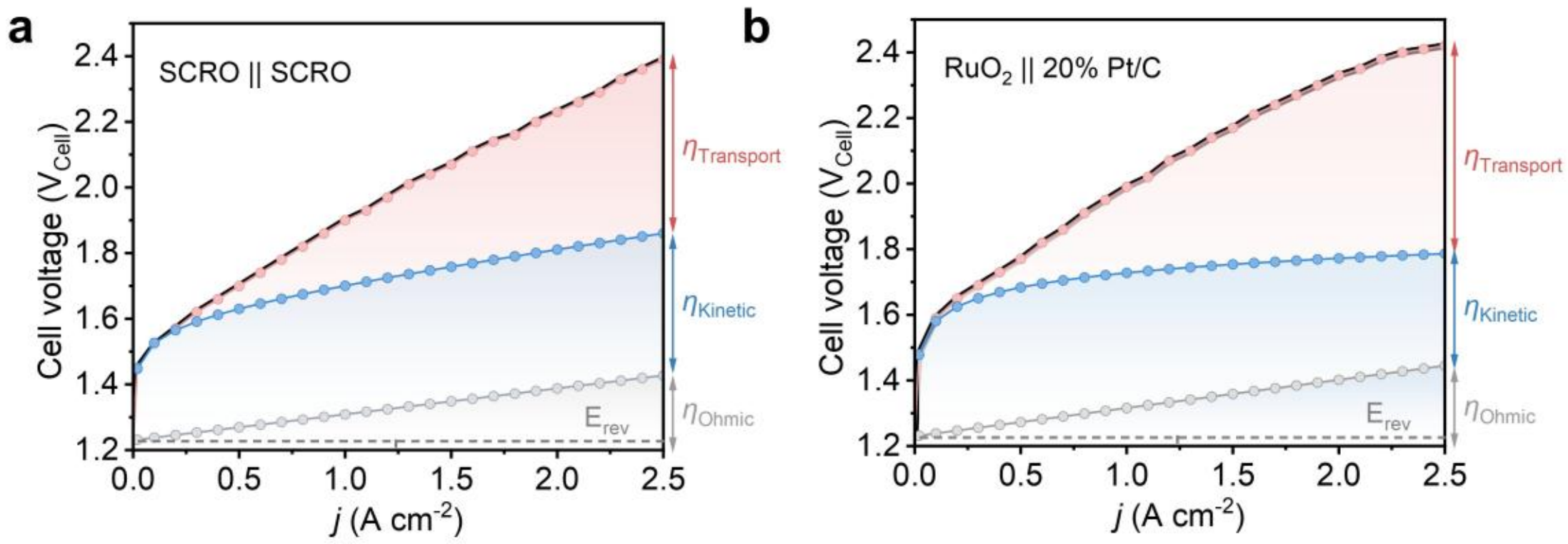

Supplementary Figure 45. The contributions of ohmic overvoltage (η_{Ohmic}), kinetic overvoltage (η_{Kinetic}) and transport overvoltage ($\eta_{\mathrm{Transport}}$) to the AEMWE with (a) SCRO || SCRO and (b) RuO_2 || 20% Pt/C.

II. Supplementary Tables

Supplementary Table 1. Refined lattice parameters and reliability factors of SCRO,

Samples	Space group	Lattice parameters	Lattice angles	Reliability factors
SCRO	*I2/c*	a = 5.53 Å<br>b = 5.53 Å<br>c = 7.84 Å	α = 90º<br>β = 90.2º<br>γ = 90º	R_p = 5.6%<br>R_{wp} = 7.17%<br>R_{exp} = 6.68%<br>Chi^2 = 1.15
SCO	*Pnma*	a = 5.47 Å<br>b = 15.73 Å<br>c = 5.57 Å	α = 90º<br>β = 90º<br>γ = 90º	R_p = 1.97%<br>R_{wp} = 2.71%<br>R_{exp} = 1.58%<br>Chi^2 = 1.92
SRO	*Pnma*	a = 5.57 Å<br>b = 7.84 Å<br>c = 5.54 Å	α = 90º<br>β = 90º<br>γ = 90º	R_p = 6.4%<br>R_{wp} = 9.1%<br>R_{exp} = 5.27 %<br>Chi^2 = 1.73

SCO and SCRO.

Supplementary Table 2. Atomic site parameters of SCRO obtained from Rietveld refinement of the XRD data.

Atom	x	y	z	Occupancy
Sr1	0.00000	0.48978	0.25000	1.0000
Co1	0.00000	0.00000	0.00000	0.4965
Ru1	0.00000	0.00000	0.00000	0.5035
O1	0.00000	0.04012	0.25000	0.9852
O2	0.22807	0.76177	0.03923	0.9838

Supplementary Table 3. EXAFS fitting parameters of Co *K*-edge and Ru *K*-edge in pristine SCRO.

Scattering path	N	R (Å)	$\sigma^2(10^{-3}Å^2)$	R factor
Co-O	5.94±0.70	2.09	9.6	0.015
Ru-O	5.79±1.01	1.97	5.1	0.013

Supplementary Table 4. Results of Bader charge analysis.

Samples	Atoms	Charges (|e|)
SCO	Co	+1.53
SRO	Ru	+1.67
SCRO	Co	+1.48
	Ru	+1.96

Supplementary Table 5. Performance comparison of SCRO with representative state-of-the-art air electrodes for ZABs as reported to date.

Device	Liquid ZABs		Flexible ZABs
Catalyst	Peak power density ($mW\ cm^{-2}$)	Durability	Peak power density ($mW\ cm^{-2}$)
This work	216	1000 h @ 5 mA cm^{-2}	128
Single phase perovskite oxides			
$La_{0.85}Ce_{0.15}CoO_3$[1]	60	160 h @ 2 mA cm^{-2}	31
$Sr_{0.95}Nb_{0.1}Co_{0.7}Fe_{0.2}O_{3-\delta}$[2]	96	500 h @ 2 mA cm^{-2}	/
$LaMnO_{3+\delta}$[3]	198.6	/	/
S-doped $CaMnO_3$[4]	128	120 h @ 5 mA cm^{-2}	/
$PrBa_{0.5}Sr_{0.5}Co_{2-x}Fe_xO_{5+\delta}$[5]	127	150 h @ 10 mA cm^{-2}	/
$PrBa_{0.5}Sr_{0.5}Co_{1.8}Ru_{0.2}O_{5+\delta}$[6]	242.7	200 h @ 10 mA cm^{-2}	122.4
$CaMnO_3$[7]	/	/	149
$PrBa_{0.5}Ca_{0.5}Co_2O_{5+\delta}$[8]	/	150 h @ 5 mA cm^{-2}	/
Composites with perovskite oxides			
ZIF-67 MOF/BSCF[9]	/	300 h @ 5 mA cm^{-2}	/
$Ce_{0.9}Gd_{0.1}O_{2-\delta}/Pr_{0.5}Ba_{0.5}O_{3-\delta}$[10]	207	200 h @ 10 mA cm^{-2}	/
$Pt\text{-}Sr(Co_{0.8}Fe_{0.2})_{0.95}P_{0.05}O_{3-\delta}/C_{12}$[11]	122	80 h @ 5 mA cm^{-2}	/
$Co_3O_4@LaCoO_3$[12]	140	185 h @ 2 mA cm^{-2}	/
Other state-of-the-art air electrodes			
IL@HCN[13]	193	500 h @ 5 mA cm^{-2}	109
FeCoCDs/CSs[14]	200.39	700 h @ 20 mA cm^{-2}	89.68
$Fe_3C\text{-}Mo_2C/NC$[15]	171	375 h @ 10 mA cm^{-2}	/
$Cu_{2-x}Se@3D\text{-}NCNs$[16]	103.8	750 h @ 10 mA cm^{-2}	146.1
Co@MCN1_800[17]	188	120 h @ 5 mA cm^{-2}	127
Co@NCNR[18]	76.76	/	15.88

Mo-Co/N-C[19]	133	1538 h @ 5 mA cm^{-2}	/
Fe/Fe_3C@N-doped C[20]	160.5	140 h @ 10 mA cm^{-2}	61.4
FeCoNiMoW HEA[21]	116.9	660 h @ 10 mA cm^{-2}	/
Fe/SNCFs-NH_3[22]	255.84	1000 h @ 1 mA cm^{-2}	/
FePc‖CNTs‖NiCo/CP[23]	219.5	700 h @ 10 mA cm^{-2}	/
CoFeNiMnCuZnO_x[24]	102.4	930 h @ 10 mA cm^{-2}	/
Pd/FeCo[25]	117	200 h @ 10 mA cm^{-2}	/
N_3-Fe-N-Co[26]	218	680 h @ 5 mA cm^{-2}	/
CoH SA/NC[27]	191.7	/	/
Fe_1-N-C[28]	205	400@ 2 mA cm^{-2}	/
(Zn)Co-NCF[29]	160	275@ 2 mA cm^{-2}	25
Co/NHCB[30]	162.2	250 h @ 5 mA cm^{-2}	48.5
FeCo-DACs[31]	157.8	500 h @ 10 mA cm^{-2}	/
CoCuFeAgRu HEA[32]	136.53	70 h @ 10 mA cm^{-2}	148.83
$Ni(S_{0.51}Se_{0.49})_2$@NC[33]	145.5	1000 h @ 10 mA cm^{-2}	/
Co/N@CNTs@CNMF-800[34]	133	190 h @ 10 mA cm^{-2}	26.5
Fe_{Mc}-$MoAl_{1-x}B$[35]	168.2	800 h @ 10 mA cm^{-2}	/
Co-CoN_4@NCNs[36]	131.6	250 h @ 10 mA cm^{-2}	/
NPS-HPCNFs[37]	210	1000 h @ 20 mA cm^{-2}	50.6
CoFe/Fe_3C-T_2[38]	249	126 h @ 5 mA cm^{-2}	66
Co_2-N_6[39]	130.52	200 h @ 5 mA cm^{-2}	69.8
FeP/Fe_2O_3@NPCA[40]	130	160 h @ 5 mA cm^{-2}	40.8
CoFe-NiFe/NC[41]	155	400 h @ 10 mA cm^{-2}	124.5

III. Supplementary Notes

Supplementary Note 1. Co and Ru valence-state calculations from XANES

All XAS data were analyzed using the standard program Demeter. To quantify the accurate valence states of Co and Ru, standard reference compounds with well-defined oxidation states were provided to establish calibration curves between the absorption-edge energy and the oxidation state. The edge energies were extracted at fixed normalized intensities of 0.8 for Co and 0.6 for Ru, respectively. Then, the valence states of Co and Ru in SCRO under operando reaction conditions were determined by comparing the edge energies of the in-situ XANES spectra with the corresponding calibration curves.

Supplementary Note 2. Computational methods

All the density functional theory (DFT) calculations were performed in the Vienna ab initio simulation package (VASP version 6.4.3)[42,43]. The projector augmented-wave (PAW) method was employed to accurately describe the interactions between valence electrons and ionic cores[44,45]. The electronic exchange-correlation effects were evaluated through the Perdew-Burke-Ernzerhof (PBE) functional based on the generalized gradient approximation (GGA), referred to as PBE-GGA[46]. The DFT-D3 correction method was employed to account for the effect of van der Waals interaction. For all structural relaxation conditions, the convergence criteria are as follows: the convergence accuracy of the electronic structure self-consistent cycle is set to 1×10^{-5} eV, the energy cutoff for plane waves is set at 450 eV, and the convergence precision for Hellmann-Feynman forces is specified as 0.02 eV/Å[47]. The atomic coordinates of the optimized SCRO, SCO and SRO computational models used in the DFT calculations are provided in Supplementary Data 1.

Supplementary Note 3. Overvoltage contribution calculations for AEMWE

The overvoltage was divided into three resistances: ohmic overvoltage (η_{Ohmic}), kinetic overvoltage ($\eta_{Kinetic}$) and transport overvoltage ($\eta_{Transport}$). The value of η_{Ohmic} was derived from electrochemical impedance spectroscopy results, as described by the Equation (3):

$$\eta_{Ohmic} = j \times HFR \text{ (V)} \quad (3)$$

where j is current density (A cm^{-2}), and HFR is the high frequency resistance (Ω cm^{-2}) measured at the current density of 0.5 A cm^{-2}.

The overvoltage contributions were further analyzed by correcting E_{cell} from polarization curves for ohmic resistance, yielding $E_{ohmic\text{-}corrected}$. From the corrected polarization curve, the Tafel slope in the low current density region was determined. Extrapolation of the fitted Tafel line to the maximum current density allowed for the determination of $\eta_{kinetic}$ using Equation (4):

$$\eta_{Kinetic} = a + b \times \log j \text{ (V)} \quad (4)$$

where j is the applied current density.

The value of $\eta_{Transport}$ was obtained by Equation (5):

$$\eta_{Transport} = E_{cell} - \eta_{Ohmic} - \eta_{Kinetic} - E_{eq} \text{ (V)} \quad (5)$$

where E_{eq} is the thermodynamic equilibrium potential of 1.23 V.

IV. Supplementary References

1. Qian, J. *et al.* Engineered spin state in Ce doped $LaCoO_3$ with enhanced electrocatalytic activity for rechargeable Zn-air batteries. *Nano Energy* **74**, 104948 (2020).
2. Kim, S. *et al.* Exceptionally durable CoFe-exsolved $Sr_{0.95}Nb_{0.1}Co_{0.7}Fe_{0.2}O_{3-\delta}$ catalyst for rechargeable Zn-air batteries. *Appl. Catal., B* **315**, 121553 (2022).
3. Kuai, L. *et al.* Mesoporous $LaMnO_{3+\delta}$ perovskite from spray-pyrolysis with superior performance for oxygen reduction reaction and Zn-air battery. *Nano Energy* **43**, 81-90 (2018).
4. Peng, S. *et al.* Electronic and defective engineering of electrospun $CaMnO_3$ nanotubes for enhanced oxygen electrocatalysis in rechargeable zinc–air batteries. *Adv. Energy Mater.* **8**, 1800612 (2018).
5. Bu, Y. *et al.* A highly efficient and robust cation ordered perovskite oxide as a bifunctional catalyst for rechargeable zinc-air batteries. *ACS Nano* **11**, 11594-11601 (2017).

6. Du, J. *et al.* Synergistic vertical graphene-exsolved perovskite to boost reaction kinetics for flexible zinc-air batteries. *Adv. Funct. Mater.* **35**, 2415351 (2024).

7. Yang, F. *et al.* Octahedral distortion enhances exceptional oxygen catalytic activity of calcium manganite for advanced Zn-air batteries. *Nano Energy* **85**, 106020 (2021).

8. Wang, Y. *et al.* Accelerated deprotonation with a hydroxy-silicon alkali solid for rechargeable zinc-air batteries. *Nat. Commun.* **14**, 6968 (2023).

9. Arafat, Y. *et al.* A porous nano-micro-composite as a high-performance Bi-functional air electrode with remarkable stability for rechargeable zinc-air batteries. *Nano Micro Lett.* **12**, 130 (2020).

10. Gui, L. *et al.* Oxygen vacancies-rich $Ce_{0.9}Gd_{0.1}O_{2-\delta}$ decorated $Pr_{0.5}Ba_{0.5}CoO_{3-\delta}$ bifunctional catalyst for efficient and long-lasting rechargeable Zn-air batteries. *Appl. Catal., B* **266**, 118656 (2020).

11. Wang, X. *et al.* High-performance platinum-perovskite composite bifunctional oxygen electrocatalyst for rechargeable Zn-air battery. *Adv. Energy Mater.* **10**, 1903271 (2019).

12. Ran, J. *et al.* Atomic-level coupled spinel@perovskite dual-phase oxides toward enhanced performance in Zn-air batteries. *J. Mater. Chem. A* **10**, 1506-1513 (2022).

13. Tu, W. *et al.* Designing hydrophobic micro-three-phase reaction interfaces to enhance the ORR kinetics toward zinc-air battery. *Adv. Energy Mater.* **15**, 2404946 (2025).

14. Li, T. *et al.* Enhanced bifunctional oxygen electrocatalysis by synergistic active heterostructure design. *Adv. Energy Mater.* **15**, 2502493 (2025).

15. Jiang, J. *et al.* Utilizing molybdenum to tailor the electronic structure of iron through electron complementary effect for promoting oxygen reduction activity. *Adv. Funct. Mater.* **35**, 2500065 (2025).

16. Chen, W. *et al.* Pyridinic-N-Cu-Se interfacial synergy enables stable bifunctional oxygen electrocatalysis. *Adv. Funct. Mater.* **36**, e17329 (2025).

17. Banerjee, M. *et al.* Powering the future: a cobalt-based catalyst for longer-lasting

zinc–air batteries. *Adv. Funct. Mater.* e19329 (2025).

28. Yu, Q. *et al.* ZIF -mediated anchoring of Co species on N-doped carbon nanorods as an efficient cathode catalyst for Zn-air batteries. *Energy Environ. Mater.* **6**, e12389 (2022).

19. Zhang, Y. *et al.* Fast-charging zinc-air batteries with stable bifunctional oxygen electrocatalysis of Mo-induced nanodefective Mo-Co/N-C catalyst. *Adv. Funct. Mater.* **34**, 2314622 (2024).

20. Wang, Z. *et al.* Prussian blue-derived atomic Fe/Fe_3 C@N-doped C catalysts supported by carbon cloth as integrated air cathode for flexible Zn-air batteries. *Adv. Sci.* **12**, 2407631 (2024).

21. He, R. *et al.* A 3d-4d-5d high entropy alloy as a bifunctional oxygen catalyst for robust aqueous zinc-air batteries. *Adv. Mater.* **35**, 2303719 (2023).

22. Yang, L. *et al.* Atomic Fe-N_4 /C in flexible carbon fiber membrane as binder-free air cathode for Zn-air batteries with stable cycling over 1000 h. *Adv. Mater.* **34**, 2105410 (2021).

23. Ding, S. *et al.* Carbon-nanotube-bridging strategy for integrating single Fe atoms and NiCo nanoparticles in a bifunctional oxygen electrocatalyst toward high-efficiency and long-life rechargeable zinc-air batteries. *Adv. Energy Mater.* **12**, 2202984 (2022).

24. Ge, H. *et al.* Polyoxometallate cluster induced high-entropy oxide sub-1 nm nanosheets as photoelectrocatalysts for Zn-air batteries. *J. Am. Chem. Soc.* **146**, 10735-10744 (2024).

25. Pan, F. *et al.* Porous FeCo glassy alloy as bifunctional support for high-performance Zn-air battery. *Adv. Energy Mater.* **11**, 2002204 (2020).

26. Yi, X. *et al.* Local single Co sites at the second shell of Fe-N_4 active sites to boost oxygen reduction reaction. *Adv. Funct. Mater.* **34**, 2309728 (2023).

27. Xie, X. *et al.* A simultaneous modulation strategy to construct high dense and accessible Co-N_4 sites for promoting oxygen reduction reaction in Zn-air battery. *Adv. Funct. Mater.* **34**, 2316037 (2024).

28. Liu, Z. *et al.* Decoration of NiFe-LDH nanodots endows lower Fe-*d* band center

of Fe_1 -N-C hollow nanorods as bifunctional oxygen electrocatalysts with small overpotential gap. *Adv. Energy Mater.* **13**, 2203609 (2023).

29. Ma, Y. *et al.* Highly dispersive Co@N-C catalyst as freestanding bifunctional cathode for flexible and rechargeable zinc-air batteries. *Energy Environ. Mater.* **5**, 543-554 (2022).
30. Wang, F. *et al.* Cognate cobalt core-shell structure decorated nitrogen-doped hollow carbon bowls triggering advanced zinc-air battery. *Adv. Funct. Mater.* **35**, 2415326 (2024).
31. Qiao, J. *et al.* Precisely constructing orbital-coupled Fe-Co dual-atom sites for high-energy-efficiency Zn-air/iodide hybrid batteries. *Adv. Mater.* **36**, 2405533 (2024).
32. Qiu, Z. *et al.* High-entropy Ag-Ru-based electrocatalysts with dual-active-center for highly stable ultra-low-temperature zinc-air batteries. *Angew. Chem., Int. Ed.* **64**, e202415216 (2024).
33. Ding, K. *et al.* Dianion induced electron delocalization of trifunctional electrocatalysts for rechargeable Zn-air batteries and self-powered water splitting. *Adv. Funct. Mater.* **32**, 2201944 (2022).
34. Liu, T. *et al.* A facile and scalable strategy for fabrication of superior bifunctional freestanding air electrodes for flexible zinc-air batteries. *Adv. Funct. Mater.* **30**, 2003407 (2020).
35. Nam, S. W. *et al.* Molecularly engineered potential of d-orbital modulated iron-bridged delaminated MBene for rechargeable Zn-air batteries. *Energy Environ. Sci.* **17**, 6559-6570 (2024).
36. Ding, K. *et al.* Robust electronic correlation of Co-CoN_4 hybrid active sites for durable rechargeable Zn-air batteries. *Adv. Funct. Mater.* **32**, 2207331 (2022).
37. Xu, X. *et al.* Electronic structure modulation in N, P, S tri-doped nanofibers with interpenetrated pores for enhanced oxygen reduction reaction. *Adv. Energy Mater.* **15**, 2405236 (2025).
38. Das, S. *et al.* Harnessing the trade-off between CoFe/Fe_3 C interfacial junction with unparalleled potential gap of 0.58 V for reversible oxygen electrocatalysis:

application toward liquid and solid-state Zn-air batteries. *Adv. Funct. Mater.* **34**, 2407078 (2024).

39. Lu, T. *et al.* Self-adjustment of intrinsic reaction intermediate on atomically dispersed Co_2-N_6 binuclear sites achieving boosted electrocatalytic oxygen reduction performance. *Adv. Funct. Mater.* **34**, 2405564 (2024).
40. Wu, K. *et al.* An iron-decorated carbon aerogel for rechargeable flow and flexible Zn-air batteries. *Adv. Mater.* **32**, 2002292 (2020).
41. Yang, X. *et al.* Biphasic nanoalloys-based trifunctional monolith for high-performance flexible Zn-air batteries and self-driven water splitting. *Adv. Funct. Mater.* **34**, 2402933 (2024).
42. Kresse, G. & Furthmüller, J. Efficiency of ab-initio total energy calculations for metals and semiconductors using a plane-wave basis set. *Comput. Mater. Sci.* **6**, 15-50 (1996).
43. Kresse, G. & Hafner, J. *Ab initio* molecular-dynamics simulation of the liquid-metal-amorphous-semiconductor transition in germanium. *Phys. Rev. B* **49**, 14251-14269 (1994).
44. Blöchl, P. E. Projector augmented-wave method. *Phys. Rev. B* **50**, 17953-17979 (1994).
45. Perdew, J. P., Burke, K. & Ernzerhof, M. Generalized gradient approximation made simple. *Phys. Rev. Lett.* **77**, 3865-3868 (1996).
46. Hammer, B., Hansen, L. B. & Nørskov, J. K. Improved adsorption energetics within density-functional theory using revised perdew-burke-ernzerhof functionals. *Phys. Rev. B* **59**, 7413-7421 (1999).
47. Barth, U. V. & Hedin, L. A local exchange-correlation potential for the spin polarized case. *J. Phys. C: Solid State Phys.* **5**, 1629-1642 (1972).
