# Supplementary material for: Trifunctional electrocatalyst with accurate surface reconstruction for zinc-air batteries and water electrolyzers
Source: Nat Commun. 2026 Jun 23;17:7890. doi: 10.1038/s41467-026-74714-5 (PMC13444124; doi:10.1038/s41467-026-74714-5)
Supplement: Supplementary file 2 [file 41467_2026_74714_MOESM2_ESM.pdf]

Description of Additional Supplementary Files

File Name: Supplementary Data 1

Description: Initial and optimized computational models of SCRO, SCO and SRO used in the DFT calculations.

This file contains the atomic coordinates of the initial and optimized computational models of SCRO, SCO and SRO used in the electronic structure calculations.

File Name: Supplementary Movie 1

Description: Self-powered water splitting driven by two SCRO-based ZABs.

The movie shows gas bubble evolution during operation of a self-powered water-splitting system composed of two series-connected SCRO-based rechargeable ZABs and a two-electrode water-splitting device.

File Name: Supplementary Movie 2

Description: Solar cell-AEMWE system operation under fluctuating sunlight.

The movie shows the operation of an SCRO-based AEMWE coupled with a commercial solar cell under outdoor conditions, with gas evolution observed under fluctuating solar power input.
