# Supplementary material for: Trifunctional electrocatalyst with accurate surface reconstruction for zinc-air batteries and water electrolyzers
Source: Nat Commun. 2026 Jun 23;17:7890. doi: 10.1038/s41467-026-74714-5 (PMC13444124; doi:10.1038/s41467-026-74714-5)
Supplement: Supplementary file 3 [file 41467_2026_74714_MOESM6_ESM.pdf]

nature portfolio

Peer Review File

Trifunctional Electrocatalyst with Accurate Surface Reconst ruction for Zinc Air Batteries and Water Electrolyzers

Corresponding Author: Professor Yinlong Zhu

Parts of this Peer Review File have been redacted as indicated to remove third-party material.

This file contains all reviewer reports in order by version, followed by all author rebuttals in order by version.

Version 0:

Reviewer comments:

Reviewer #1

(Remarks to the Author)
In this manuscript the authors report the synthesis of $Sr_2CoRuO_{6-\delta}$ (SCRO) catalysts with double perovskite structure. These catalysts show high activity for alkaline OER, HER and ORR. The electrochemical performance was evaluated using both 3-electrode cells and full-cell devices, including evaluating the ORR performance using a rechargeable Zn-air battery and the HER and OER performance using an anion exchange membrane water electrolyzer. Furthermore, the authors provided extensive in-situ X-ray absorption spectroscopy (XAS) and X-ray diffraction (XRD) to identify the oxidation states and structural properties of the SCRO catalysts under OER, HER and ORR conditions. This is a very comprehensive study. The authors should address the following comments before the manuscript can be accepted.
Comments:
1. In the XANES results in Figure if, the oxidation state of Co in SCRO was attributed to Co^{3+} due to the similar energy position of Co-K XANES spectra between SCRO and $LaCoO_3$. Although this conclusion is reasonable, it is complicated by the observation that the Co K-edge peak height of $LaCoO_3$ is much higher than that of SCRO, suggesting that the oxidation state is less than CO^{3+} in SCRO. The authors need to clearly explain why the energy position and peak height lead to different conclusions regarding the Co oxidation state.
2. The calculated Bader charge of Co and Ru are +1.48 |e| and +1.96 |e|, respective in SCRO, compared to +1.53 |e| for Co in $SrCoO_3$ and +1.67 |e| for Ru in $SrRuO_3$. The authors correctly conclude that these changes imply the electron donation from Ru to Co ions in SCRO. However, the relatively magnitudes of changes in the Bader charge are somewhat puzzling, i.e., a decrease in Co (0.05 e) is much less than the increase in Ru (0.29 e). What is the reason for this? Does this mean that Ru also donates charge to other elements?
3. The authors compared the HER and OER performance of the SCRO electrocatalysts in an anion exchange membrane water electrolyzer (AEMWE). The results show a better performance than the benchmark RuO_2 || 20%Pt/C catalysts, with the SCRO HER/OER catalysts reaching the industrial current of 1000 mA cm^{-2} at a low voltage of 1.90 V. In order to further understand the origin of the improved performance, it is important for the authors to compare the 3-electrode performance of SCRO with benchmark of RuO_2 for OER and 20%Pt/C for HER. These results should be included for comparison in the 3-electrode results in Figure 2.
4. In Figure 4, under HER, the average oxidation state of Co decreases from 2.99+ to 2.56+ while Ru decreases from 4.83+ to 3.38+. A schematic is shown to illustrate the exsolution to make monometallic Ru and bimetallic CoRu nanoparticles. The oxidations states of Ru and Co should be zero in these metallic particles, while Co and Ru in the perovskite structure should remain oxidized. Can the change in average oxidation state be used to estimate the percentage of Ru and CoRu exsolution?
5. There are well-established descriptors for OER, HER and ORR based on the binding energies of key intermediates. The authors already performed DFT calculations of the Bader charge. If the authors can perform relatively simple calculations of binding energies of key intermediates over the proposed SCRO structures under conditions, it would significantly strengthen the conclusions that SCRO shows enhanced alkaline OER, HER and ORR activities over the benchmark RuO_2 and Pt catalysts.

Reviewer #2

(Remarks to the Author)
Review Comments
This manuscript presents a comprehensive and impressive study on the $Sr_2CoRuO_{6-δ}$ (SCRO) double perovskite as a trifunctional electrocatalyst. The combination of advanced in-situ characterization with practical device demonstration is a significant strength. However, to meet the high standards of this journal, the following major concerns must be addressed.

Comment 1:
The authors employed BET specific surface area to calculate “specific activity” and “mass activity” (Figures 2b, 2e). For bulk micrometer-sized oxide particles with low porosity (as indicated by scanning electron microscopy), BET specific surface area may not accurately reflect the electrochemically accessible surface area. The authors should: cross-validate the three catalysts by measuring electrochemical double-layer capacitance (Cdl) to estimate electrochemical active surface area (ECSA).

Comment 2:
The authors attribute the enhanced performance to "super-exchange interactions" between Co^{3+} and Ru^{5+}. While the XAS and Bader charge analysis support charge redistribution, the direct link between super-exchange and catalytic activity is not fully established. The manuscript would benefit from a more explicit discussion of how super-exchange (e.g., ferromagnetic coupling) facilitates electron transfer or optimizes intermediate adsorption. DFT calculations comparing the electronic structure of SCRO with SCO and SRO, specifically in terms of d-band center or oxygen p-band center, would provide more concrete evidence.

Comment 3:
The in-situ XAS experiments are a major strength of this work. However, the authors should discuss potential artifacts, such as beam-induced damage or changes in the electrolyte layer thickness during measurements. Additionally, the time resolution of the measurements (e.g., how long each spectrum took to acquire) should be reported, as this affects the interpretation of dynamic structural changes.

Comment 4:
The authors normalize OER and HER activity to the "real oxide surface area" (ROSA) determined by BET. For bulk oxide particles (micrometer-sized as shown in SEM), BET surface area may not accurately reflect the electrochemically accessible surface area, especially if the particles are non-porous. The authors should consider using electrochemical surface area (ECSA) methods (e.g., double-layer capacitance) to cross-validate the ROSA values. Alternatively, they should discuss the limitations of using BET for bulk materials.

Comment 5:
The SCRO-based liquid ZAB exhibits exceptional stability for 1000 h at 5 mA cm^{-2}. While impressive, this current density is relatively low for practical applications. The authors should provide cycling data at higher current densities (e.g., 10 or 20 mA cm^{-2}) to demonstrate the catalyst's durability under more demanding conditions. This would also allow for a fairer comparison with many literature reports that test at 10 mA cm^{-2}.

Comment 6:
The AEMWE achieves 1 A cm^{-2} at 1.90 V, which is commendable. The breakdown of overvoltage contributions ($η_{Ohmic}$, $η_{Kinetic}$, $η_{Transport}$) is insightful. However, the method for calculating $η_{Kinetic}$ (using a "linear approximation in the Tafel region") is not clearly described. The authors should specify the potential range used for the Tafel fit and provide the Tafel plots for the full cell. Additionally, the stability test at 0.5 A cm^{-2} for 100 h shows a decay rate of 0.8 mV h^{-1}. While low, the authors should discuss whether this decay is due to catalyst degradation, membrane degradation, or other factors, and provide post-mortem characterization of the electrodes.

Reviewer #3

(Remarks to the Author)
This manuscript reports a single-phase $Sr2CoRuO6-δ$ double perovskite with super-exchange double perovskite structure as highly efficient trifunctional electrocatalyst for OER, HER and ORR simultaneously. Combining comprehensive in-situ techniques, surface reconstruction behaviors of $Sr2CoRuO6-δ$ under varied reactions were systematically investigated and true active components were accurately identified. When used in practical applications, $Sr2CoRuO6-δ$ shows multi-condition adaptability for Zn-air battery and water electrolysis with attractive performance parameters. Overall, the results are impressive where experiments were conducted with rigor, and the manuscript was well organized. I feel that this is an interesting and timely paper in the field of developing multifunctional electrocatalysts for emerging energy conversion and storage technologies, and I recommend for publications after minor revisions.
1.TEM evidence for surface reconstruction behavior remains somewhat limited, as only a single image was presented for each reconstructed state. The authors should provide clear overview images of the reconstructed particles, together with additional TEM images acquired from multiple representative regions.
2.The rate performance data of the SCRO-based ZAB device indicate good reversibility, as the discharge voltage recovers

to the initial level after the current density is increased to 20 mA cm-2 and subsequently switched back to 1 mA cm-2. Nevertheless, a maximum current density of 20 mA cm-2 may not be sufficiently demanding to fully support the claimed reversibility. The authors are encouraged to extend the rate test to higher current densities, which would allow a more rigorous assessment of device performance under harsher operating conditions.
3.In Figure 6i, the demonstration of using flexible Zn-air battery to charge a mobile phone is interesting and effectively highlights the practical potential of the device. To further strengthen this result, the authors should provide additional experimental details, such as the working voltage of two flexible Zn-air batteries connected in series.
4.For AEMWE tests, catalyst powders typically face challenges such as peeling or detachment from the electrode surface during vigorous gas evolution. The authors are encouraged to provide more experimental details to clarify how such issues were mitigated or avoided in the present device configuration.
5.A labeling inconsistency appears to be present in Figure 6. The figure caption uses “20% Pt/ +RuO2”, while the main text refers to the benchmark catalyst as “20% Pt/C+RuO2”. The authors should check and standardize this notation throughout the manuscript for clarity.
6.Atomic site occupancy parameters for SCRO obtained from XRD structure refinement is suggested to be disclosed. Additionally, a comprehensive description of the data analysis protocol for quantifying Co oxidation state evolution via in-situ XANES spectroscopy under operando reaction conditions is required.
7.In Figure 2d&f, 10 mA cm-2 should be -10 mA cm-2 and please modify. The figures in the manuscript are of low resolution. Please provide clearer ones.

Version 1:

Reviewer comments:

Reviewer #1

(Remarks to the Author)
The revisions solved my comments.

Reviewer #2

(Remarks to the Author)
The author has addressed and revised each of my comments, and I agree and appreciate the effort. However, there is still a minor issue: in Supporting Information figure 14d, the legend for the curve does not match the results in Supporting Information figure14(a–c). Please make the necessary corrections.Once the changes have been made, we recommend accepting them.

Reviewer #3

(Remarks to the Author)
The authors have well addressed my concerns, now the revised version of this manuscript could be accepted from my side.

Open Access This Peer Review File is licensed under a Creative Commons Attribution 4.0 International License, which permits use, sharing, adaptation, distribution and reproduction in any medium or format, as long as you give appropriate credit to the original author(s) and the source, provide a link to the Creative Commons license, and indicate if changes were made.
In cases where reviewers are anonymous, credit should be given to 'Anonymous Referee' and the source.
The images or other third party material in this Peer Review File are included in the article's Creative Commons license, unless indicated otherwise in a credit line to the material. If material is not included in the article's Creative Commons license and your intended use is not permitted by statutory regulation or exceeds the permitted use, you will need to obtain permission directly from the copyright holder.
To view a copy of this license, visit https://creativecommons.org/licenses/by/4.0/

Point-to-Point Responses to Reviewers' Comments and Suggestions

First of all, we thank the reviewers for their valuable comm ents and suggestions, which have resulted in modifications that have improved the quality and clarity of this paper. To address the specific concern/point clearly, we have separated the referees' comments into question areas and answered them in turn as follows.

Reviewer #1

<u>GENERAL COMMENTS:</u> In this manuscript the authors report the synthesis of $Sr_2CoRuO_{6-\delta}$ (SCRO) catalysts with double perovskite structure. These catalysts show high activity for alkaline OER, HER and ORR. The electrochemical performance was evaluated using both 3-electrode cells and full-cell devices, including evaluating the ORR performance using a rechargeable Zn-air battery and the HER and OER performance using an anion exchange membrane water electrolyzer. Furthermore, the authors provided extensive *in-situ* X-ray absorption spectroscopy (XAS) and X-ray diffraction (XRD) to identify the oxidation states and structural properties of the SCRO catalysts under OER, HER and ORR conditions. This is a very comprehensive study. The authors should address the following comments before the manuscript can be accepted.

<u>Response:</u>

We very much appreciate the Reviewer #1's **positive comments on our work** (*i.e.*, **This is a very comprehensive study**), and valuable recommendations for publication. According to the raised suggestions, our responses to his/her detailed comments per point are as follows.

Comment 1: In the XANES results in Figure 1f, the oxidation state of Co in SCRO was attributed to Co^{3+} due to the similar energy position of Co-K XANES spectra between SCRO and $LaCoO_3$. Although this conclusion is reasonable, it is complicated by the observation that the Co *K*-edge peak height of $LaCoO_3$ is much higher than that of SCRO, suggesting that the oxidation state is less than CO^{3+} in SCRO. The authors need to clearly explain why the energy position and peak height lead to different conclusions regarding the Co oxidation state.

<u>Response to C1:</u> We thank the reviewer for this insightful comment. <u>First</u>, we sincerely apologize for the mistake in Figure 1f preparation, in which the pristine spectrum collected in

the air was **wrongly used** with the OCP data in the *in-situ* measurements. In the revised manuscript, this error has been **corrected** with the correct Co-*K* spectrum collected in the air (**Figure R1**). Notably, the Co *K*-edge peak height of SCRO with Co^{3+} is still slightly lower than that of $LaCoO_3$. Second, we would like to clarify that for 3*d* transition-metal *K*-edge XANES spectra, the **absorption peak represents the main 4*p* states rather than 3*d* states**, and therefore the height of the main peak **does not accurately reflect the valence state of 3*d* element** (*Photosynth. Res.* 2009, 102, 241; *Phys. Rev. B* 1997, 55, 8726; *Phys. Rev. B* 1983, 27, 2111). Instead, **the absorption-edge position at a fixed normalized intensity is generally more reliable** for valence-state analysis in 3*d* transition-metal *K*-edge XANES (*Rev. Mineral. Geochem.* 2014, 78, 75; *Phys. Rev. B* 1997, 55, 8726; *Energy Environ. Sci.* 2025, 18, 7527; *J. Am. Chem. Soc.* 2025, 147, 26854; *Adv. Energy Mater.* 2024, 15, 2404599). For instance, when the absorption edge is defined at a fixed normalized intensity of ~0.8 for 3*d* transition-metal *K*-edge XANES analysis, compounds with the same valence state of 3*d* element generally exhibit comparable edge energies. This behavior has been demonstrated for Mn *K*-edge XANES (*Phys. Rev. B* 1997, 55, 8726) (**Figure R2**) and Co *K*-edge references of Co^{2+}, Co^{3+}, and Co^{4+} (**Figure R3**). Third, the surface electronic structure of Co in SCRO was further investigated by **soft XAS** at the Co-L_3 edge, whose spectral features and energy position are **sensitive to the valence states and spin states**. As shown in **Figure R4**, both energy position and spectral feature of Co-L_3 edge XAS in SCRO is **nearly identical to** that of standard Sr_2CoRuO_6 reference, also confirming the high-spin Co^{3+} state in SCRO. Overall, both Co-*K* XANES and Co-L_3 data demonstrate the Co^{3+} oxidation state in SCRO.

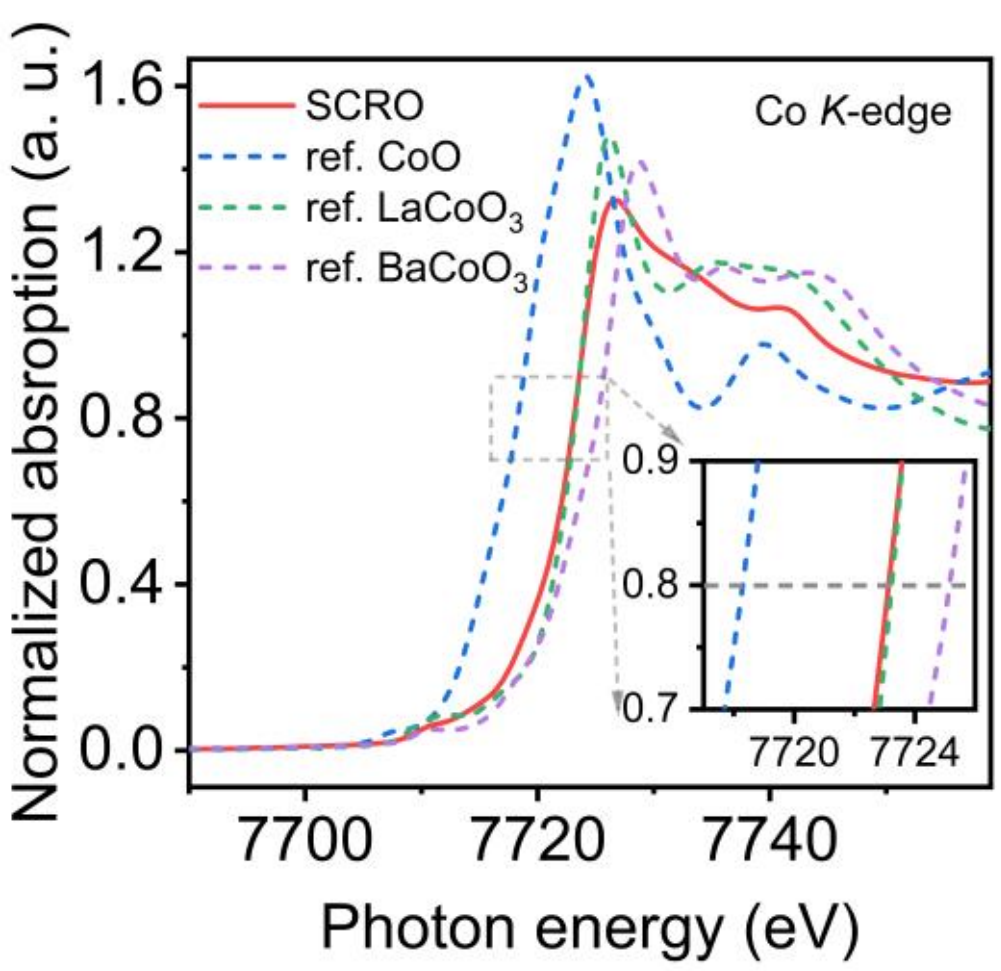

Figure R1 (also revised **Figure 1f**). Co *K*-edge XANES spectra of SCRO along with several

reference materials for comparison.

[FIGURE REDACTED]

Figure R2 (Data from *Phys. Rev. B* 1997, 55, 8726). The Mn *K*-edge spectra for $LaMnO_3$ and $CaMnO_3$, along with those of the Mn_2O_3 and MnO_2 standards.

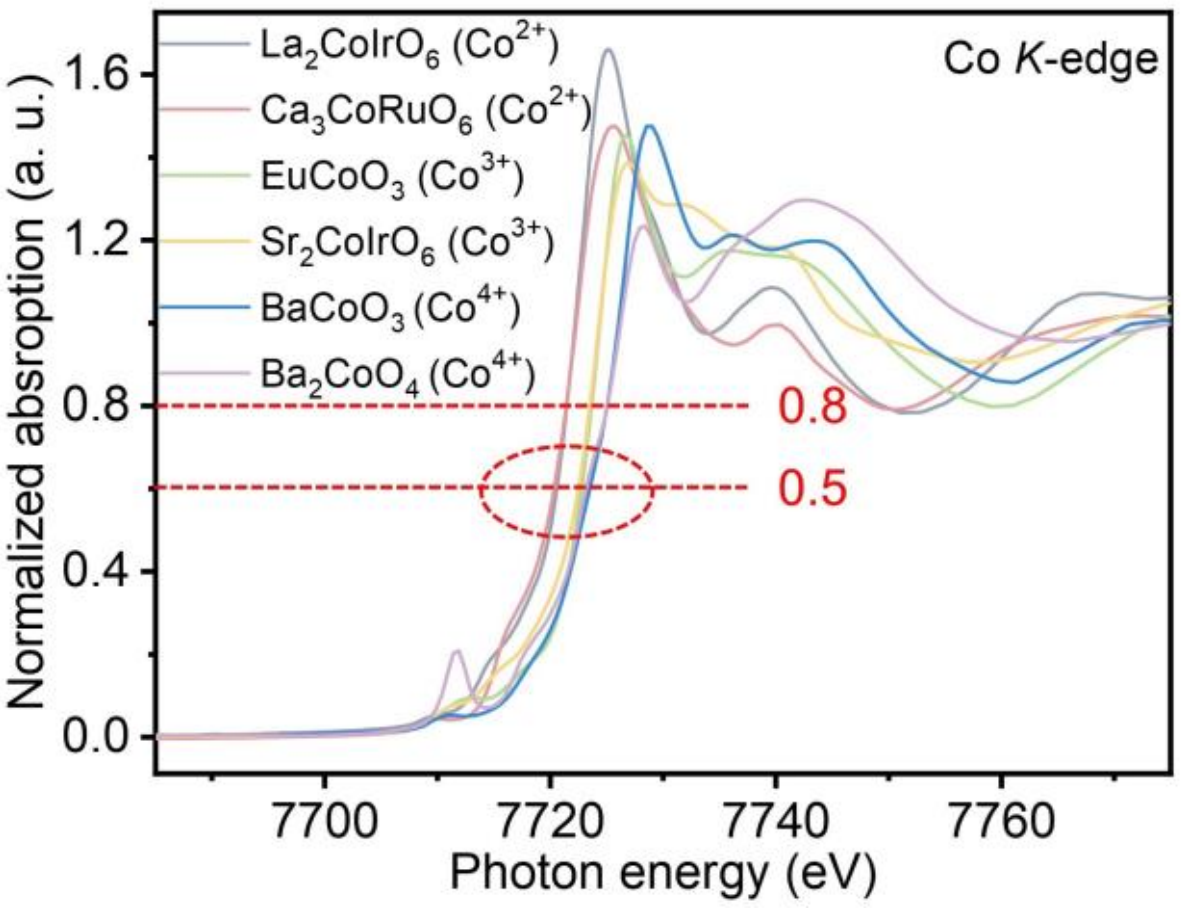

Figure R3. Co *K*-edge XANES spectra of Co^{2+}, Co^{3+}, and Co^{4+} reference compounds.

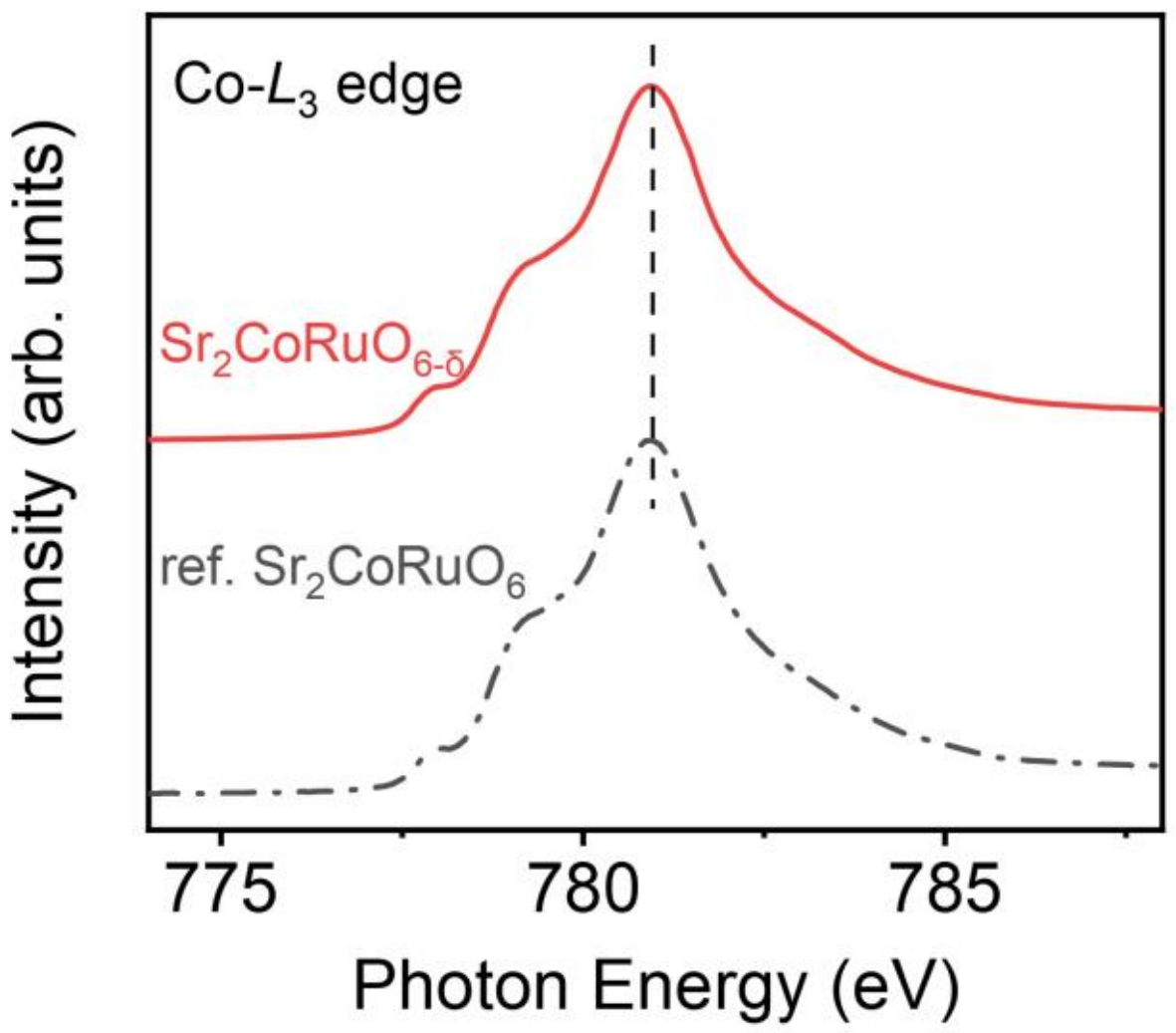

Figure R4 (also **Supplementary Figure 4a** in the original manuscript). Co L_3-edge XAS

spectra of SCRO together with that of standard Sr_2CoRuO_6 as references for high-spin Co^{3+}.

Comment 2: The calculated Bader charge of Co and Ru are +1.48 |e| and +1.96 |e|, respective in SCRO, compared to +1.53 |e| for Co in $SrCoO_3$ and +1.67 |e| for Ru in $SrRuO_3$. The authors correctly conclude that these changes imply the electron donation from Ru to Co ions in SCRO. However, the relatively magnitudes of changes in the Bader charge are somewhat puzzling, *i.e.*, a decrease in Co (0.05 e) is much less than the increase in Ru (0.29 e). What is the reason for this? Does this mean that Ru also donates charge to other elements?

Response to C2: We thank the reviewer for raising this important question. We would like to clarify that the Bader charge analysis for Ru and Co in this work is intended primarily to evaluate relative changes in electron density, **rather than** to provide a strictly quantitative measure electron transfer value. Because **Co and Ru are chemically distinct elements** with different electronic structures (*e.g.*, orbital characteristics, spin state and covalency), **the absolute values and magnitudes of their Bader charge variations are not expected to be directly comparable on the same scale**, in agreement with previous charge density studies (*e.g.*, *J. Phys. Chem. Lett.* 2017, 8, 2074; *J. Chem. Phys.* 1967, 46, 3341; *Acc. Chem. Res.* 1975, 8, 34; *J. Am. Chem. Soc.* 1971, 93, 3095, *etc*.). Despite the unequal magnitudes of changes, the Bader-charge results consistently show that Ru becomes relatively electron-deficient, whereas Co becomes relatively electron-enriched in SCRO, **supporting the super-exchange interactions between adjacent Ru and Co sites**. Such unequal Bader-charge variations associated with super-exchange interactions have been also commonly reported in previous studies (*e.g.*, *Nat. Commun.* 2020, 11, 5657; *Nat. Commun.* 2024, 15, 1565, *etc*.).

Comment 3: The authors compared the HER and OER performance of the SCRO electrocatalysts in an anion exchange membrane water electrolyzer (AEMWE). The results show a better performance than the benchmark RuO_2 || 20%Pt/C catalysts, with the SCRO HER/OER catalysts reaching the industrial current of 1000 mA cm^{-2} at a low voltage of 1.90 V. In order to further understand the origin of the improved performance, it is important for the authors to compare the 3-electrode performance of SCRO with benchmark of RuO_2 for OER and 20 % Pt/C for HER. These results should be included for comparison in the 3-electrode

results in Figure 2.

Response to C3: We thank the reviewer for this valuable suggestion. **As suggested by the reviewer**, we have added the OER polarization curve of commercial RuO_2 to **Figure 2a** and the HER polarization curve of commercial Pt/C to **Figure 2d** for direct comparison with SCRO in the revised manuscript. The polarization curves show that **SCRO requires a lower potential than commercial RuO_2** to reach 10 mA cm^{-2} for OER (**Figure R5**). For HER, **SCRO delivers a higher current density than benchmark Pt/C at potentials more negative than -0.17 V** (**Figure R6**). The comparison of 3-electrode performance with benchmark catalysts further supports the superior OER and HER electrocatalytic activity of SCRO, **consistent with its improved AEMWE performance**.

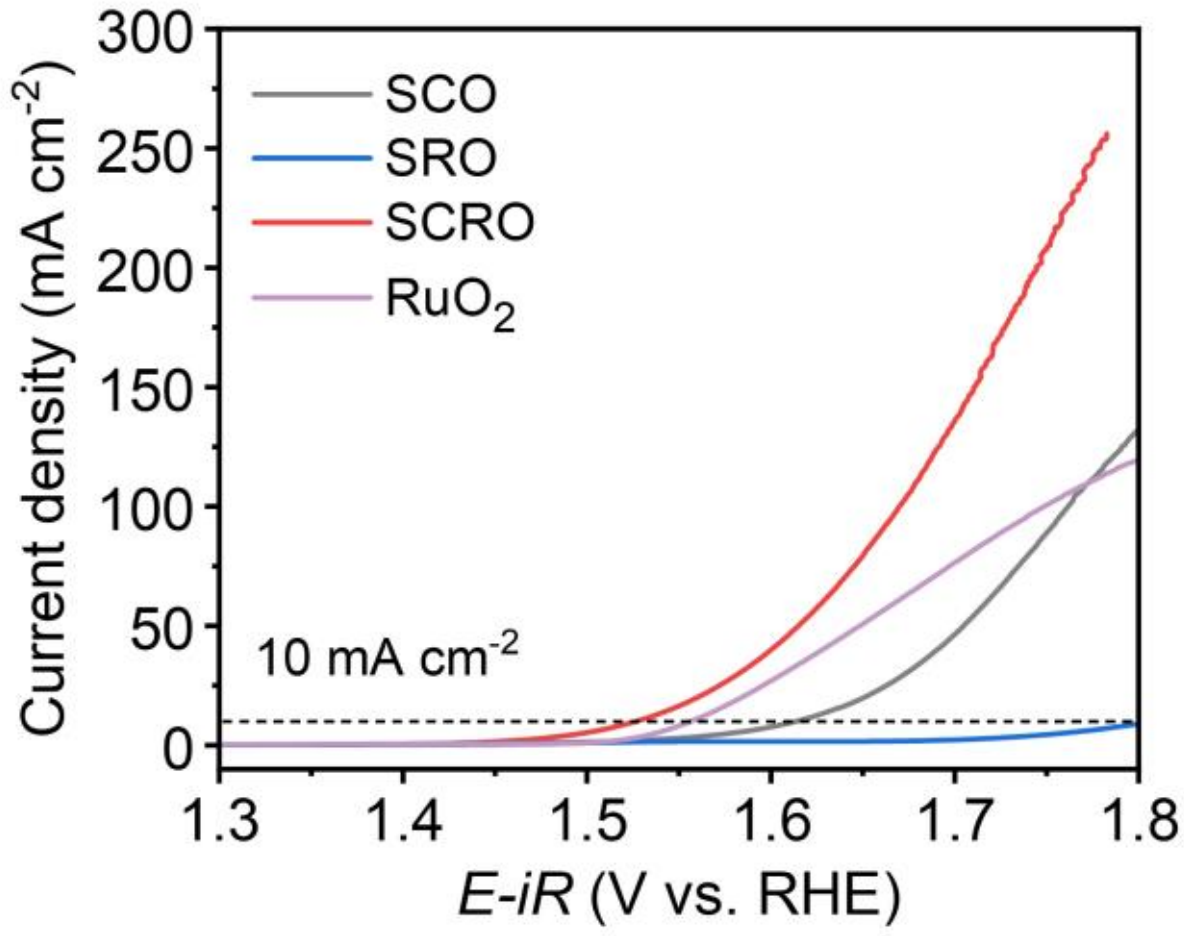

Figure R5 (also revised **Figure 2a**). OER polarization curves of SCRO, SCO, SRO and commercial RuO_2 catalysts in 1 M KOH.

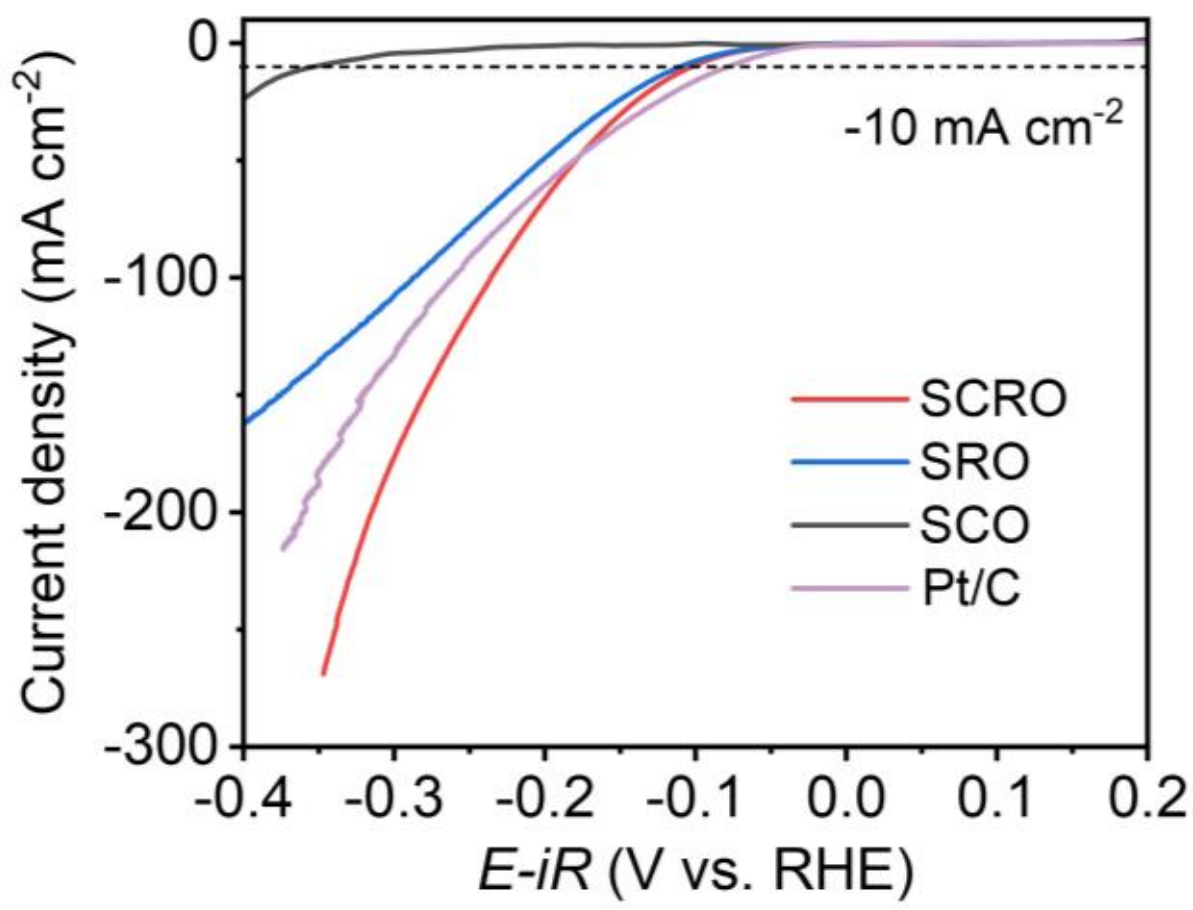

Figure R6 (also revised **Figure 2d**). HER polarization curves of SCRO, SCO, SRO and commercial 20% Pt/C catalysts in 1 M KOH.

Related data (**revised Figure 2a&d**) and discussions have been provided in the revised manuscript as follows:

*"As seen in the polarization curves obtained from linear sweep voltammetry (LSV) (**Fig. 2a**), SCRO necessitates a much lower OER overpotential (η_{10}) of 297 mV to deliver 10 mA cm^{-2} compared to SCO and SRO and commercial RuO_2, suggesting a significantly improved alkaline OER activity."* (yellow-highlighted in **Line 7-10**, **Page 8**)

"Noticeably, the HER current of SCRO can largely exceed that of the benchmark Pt/C catalyst beyond -0.17 V." (yellow-highlighted in **Line 33-34**, **Page 8**)

Comment 4: In Figure 4, under HER, the average oxidation state of Co decreases from 2.99+ to 2.56+ while Ru decreases from 4.83+ to 3.38+. A schematic is shown to illustrate the exsolution to make monometallic Ru and bimetallic CoRu nanoparticles. The oxidations states of Ru and Co should be zero in these metallic particles, while Co and Ru in the perovskite structure should remain oxidized. Can the change in average oxidation state be used to estimate the percentage of Ru and CoRu exsolution?

Response to C4: We thank the reviewer for raising this question. We agree the reviewer that **the change in average oxidation state of Co and Ru can be used to estimate the percentage of Ru and CoRu exsolution** under HER condition. **As suggested by the reviewer**, we estimate the percentage of Ru and CoRu exsolution based on the average valence changes of Co and Ru under HER conditions. Within a simplified two-state model, the apparent exsolved fractions of Co and Ru are estimated to be ~14.4% and ~30.0%, respectively. If the exsolved Co is assumed to be incorporated into CoRu alloy, **the apparent fractions of CoRu alloy and Ru metal** were estimated to be **~14.4%** and **~15.6%**, respectively.

Related discussions (yellow-highlighted in **Line 19-21, Page 12**) have been added in the revised manuscript as follows:

"Based on the changes in the average valence states relative to pristine SCRO, the apparent fractions of CoRu alloy and Ru metal were estimated to be ~14.4% and 15.6%, respectively."

Comment 5: There are well-established descriptors for OER, HER and ORR based on the binding energies of key intermediates. The authors already performed DFT calculations of the

Bader charge. If the authors can perform relatively simple calculations of binding energies of key intermediates over the proposed SCRO structures under conditions, it would significantly strengthen the conclusions that SCRO shows enhanced alkaline OER, HER and ORR activities over the benchmark RuO_2 and Pt catalysts.

Response to C5: We sincerely thank the reviewer for this valuable suggestion. First, we **fully agree that** the calculations of binding energies of key intermediates under OER/HER/ORR conditions would further support the enhanced electrocatalytic performance of SCRO. However, in our work, **such calculations are not straightforward and easy** because the real active components under electrocatalytic OER/HER/ORR reactions are completely distinct reconstructed states rather than the pristine SCRO structure. Specifically, the surface of SCRO can reconstruct into different active components under OER/HER/ORR reactions including amorphous $(Co,Ru)O_xH_y$ with high Co/Ru valence states, exsolved dual-phase CoRu alloy-Ru metal nanoparticles, and surface tensile-strained SCRO phase with reduced coordination numbers/valence states. Therefore, **a rigorous theoretical calculation would require the construction of multiple complicated reconstructed models** rather than relatively simple calculations based on the pristine SCRO structure. Particularly, such calculations **become extremely challenging under OER conditions**, where the surface reconstructs into an amorphous $(Co,Ru)O_xH_y$ phase. As reported in previous studies, rigorous identification of the true active sites on such an amorphous reconstructed surface **requires substantial computational effort**, making binding-energy calculations **very difficult** (*Nat. Commun.* 2025, 16, 8827; *Energy Environ. Sci.* 2025, 18, 1214). Besides, our work is **focused on** the demonstration of the single-phase super-exchange perovskite SCRO as a multifunctional electrocatalyst in practical devices with multi-condition adaptability, and meanwhile identifying accurate surface active components under different reaction conditions rather than detailed active sites in the active components.

Therefore, given **the great difficulty of calculations due to the complexity of the reconstructed active phases** under different reaction conditions and **the main focus in this work**, we prefer not to include such calculations in this work; further efforts could shed on this point in future theoretical investigations.

Reviewer #2

<u>GENERAL COMMENTS:</u> This manuscript presents a comprehensive and impressive study on the $Sr_2CoRuO_{6-\delta}$ (SCRO) double perovskite as a trifunctional electrocatalyst. The combination of advanced in-situ characterization with practical device demonstration is a significant strength. However, to meet the high standards of this journal, the following major concerns must be addressed.

Response: We very much appreciate the Reviewer #2's **positive comments on our work** (*e.g.,* **a comprehensive and impressive study** and **a significant strength**), and valuable recommendations to improve our manuscript. According to the raised suggestions, our responses to his/her detailed comments per point are as follows.

Comment 1: The authors employed BET specific surface area to calculate "specific activity" and "mass activity" (Figures 2b, 2e). For bulk micrometer-sized oxide particles with low porosity (as indicated by scanning electron microscopy), BET specific surface area may not accurately reflect the electrochemically accessible surface area. The authors should: cross-validate the three catalysts by measuring electrochemical double-layer capacitance (C_{dl}) to estimate electrochemical active surface area (ECSA).

<u>Response to C1:</u> We thank the reviewer for this valuable suggestion. **As suggested by the reviewer**, we have added electrochemical surface area (ECSA) analysis in the revised manuscript by measuring the electrochemical double-layer capacitance (C_{dl}) of SCRO, SRO, and SCO from CV scans recorded in the non-faradaic potential region for OER and HER, respectively (**Figure R7&R9**). As shown in **Figure R8&R10**, after normalization to the ECSA, **SCRO still exhibits much higher specific activities for both OER and HER than SCO and SRO**, consistent with the activity trend obtained by normalization to the BET specific surface area in the original manuscript.

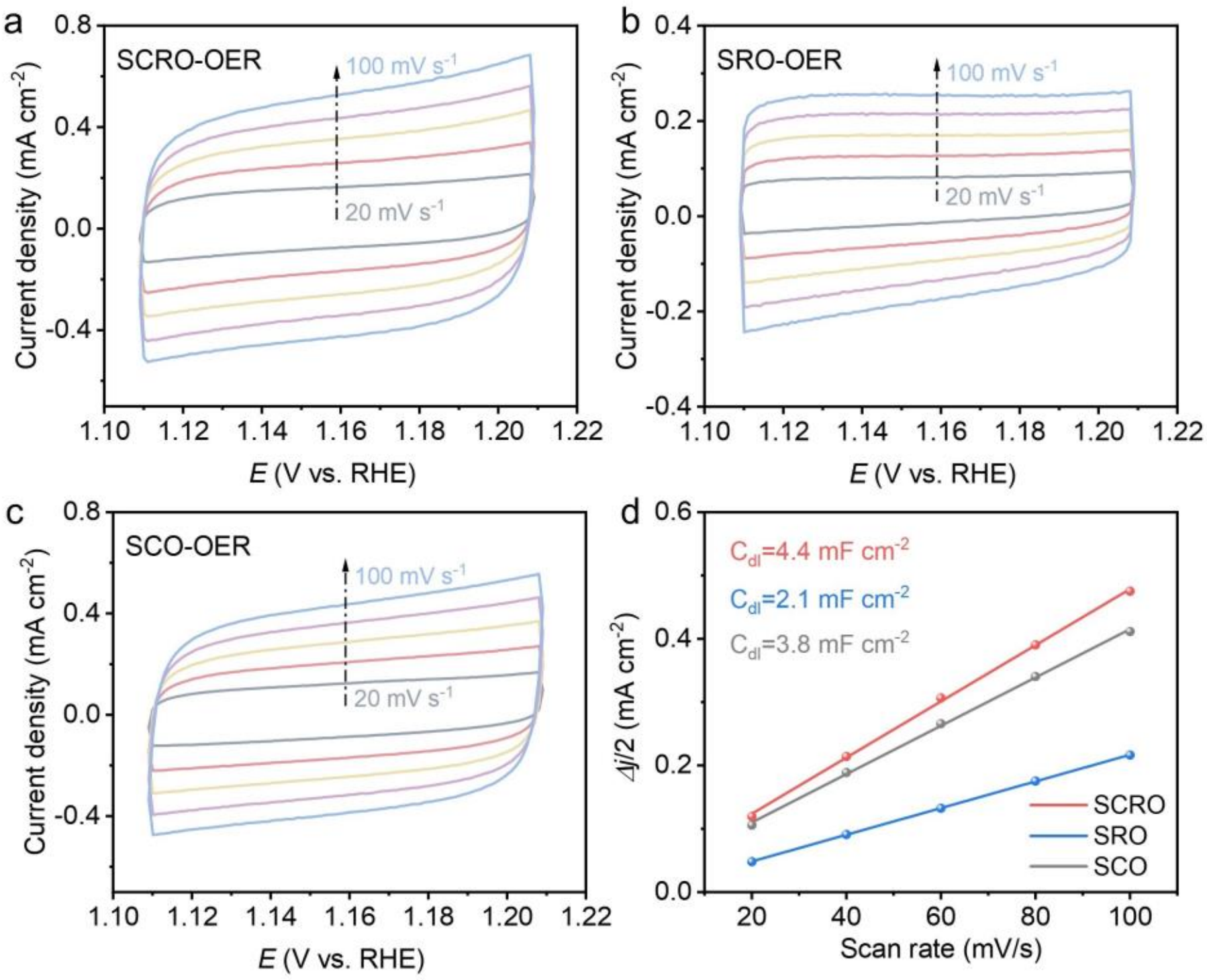

Figure R7 (also **Supplementary Figure 9**)**.** Electrochemical CV scans of (a) SCRO, (b) SRO and (c) SCO in the non-faradaic potential region for OER. (d) Calculated double layers capacitances extracted from the linear fitting of the capacitive currents versus CV scan rates for SCRO, SRO and SCO.

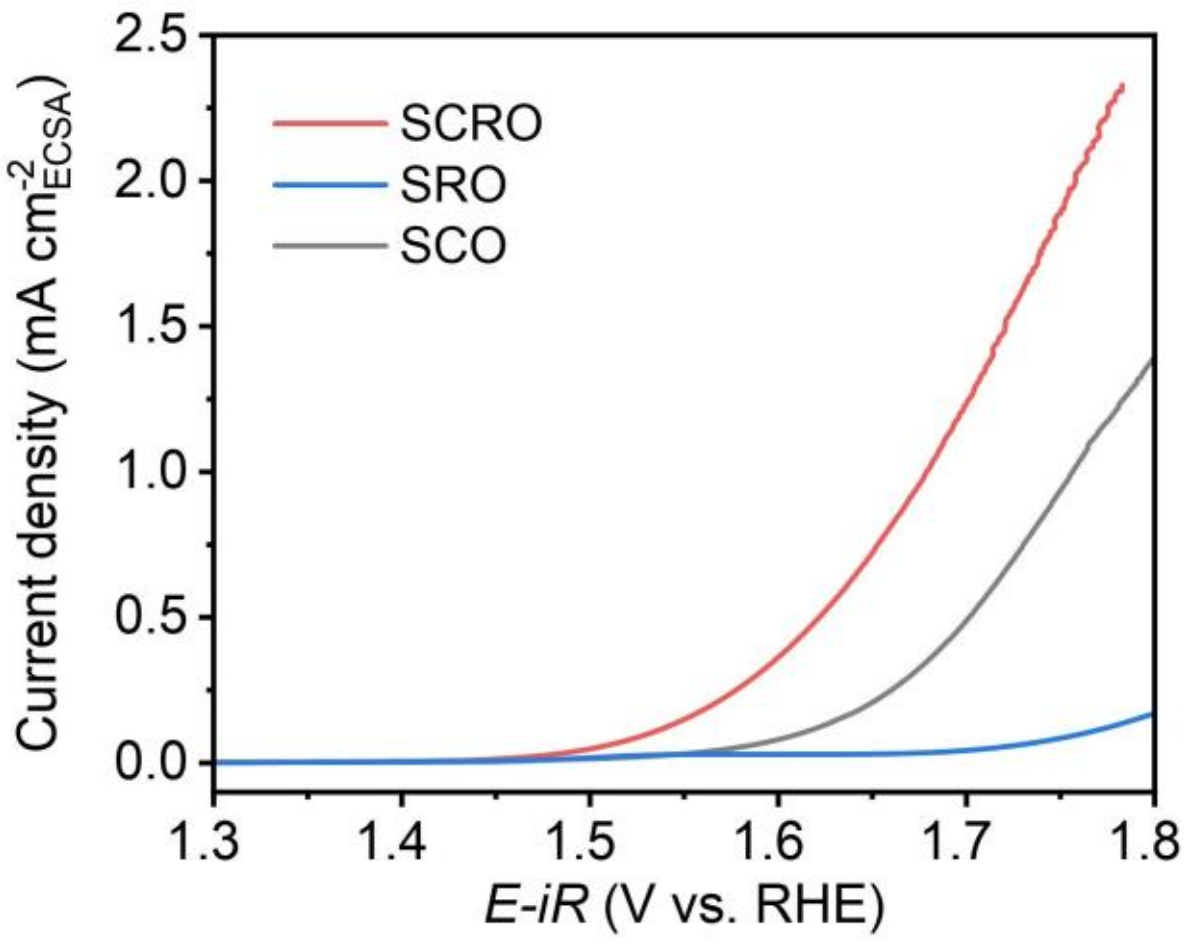

Figure R8 (also **Supplementary Figure 11**)**.** Specific activity for OER normalized to ECSA of SCRO, SCO and SRO as a function of applied potentials.

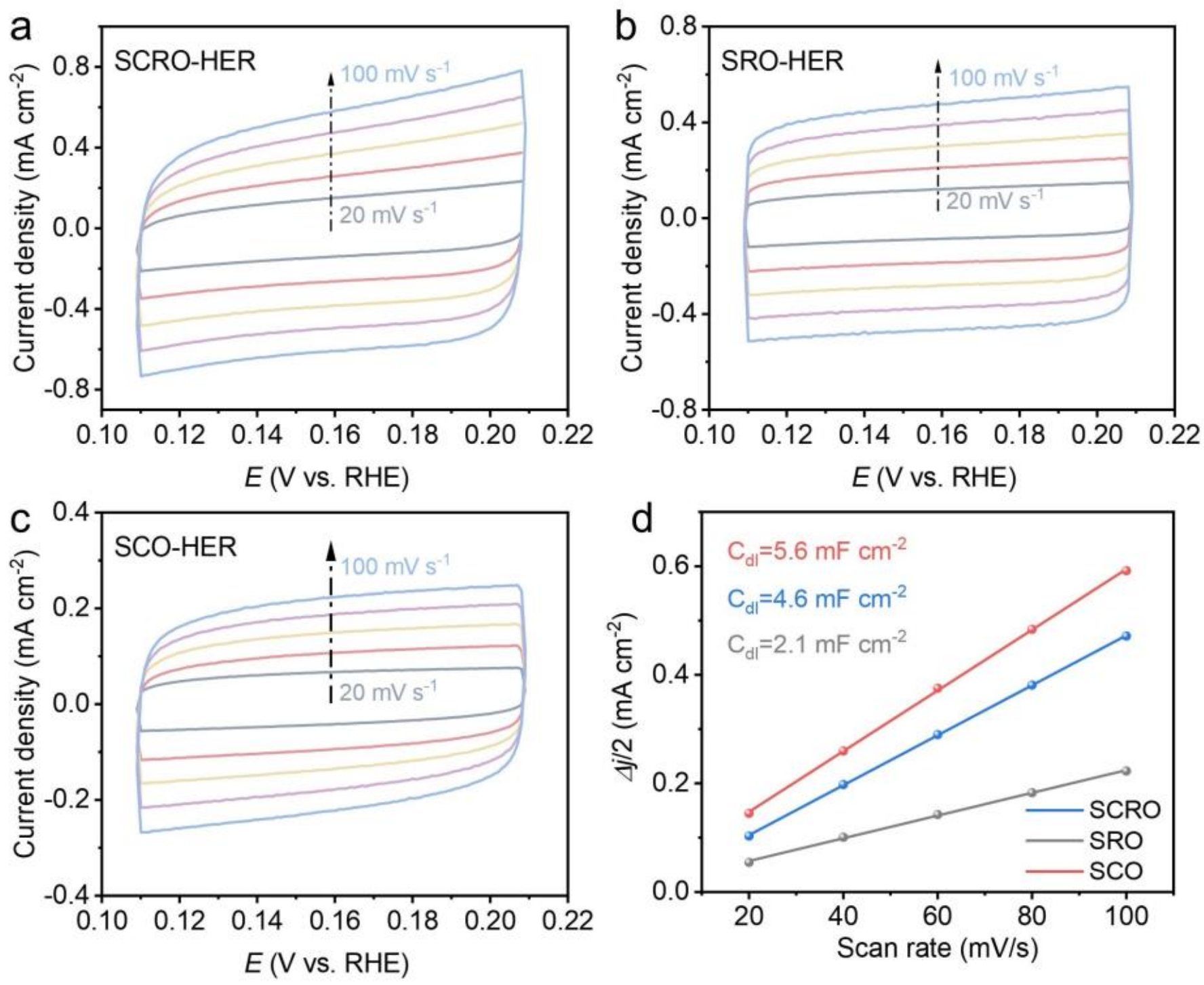

Figure R9 (also **Supplementary Figure 14**). Electrochemical CV scans of (a) SCRO, (b) SRO and (c) SCO in the non-faradaic potential region for HER. (d) Calculated double layers capacitances extracted from the linear fitting of the capacitive currents versus CV scan rates for SCRO, SRO and SCO.

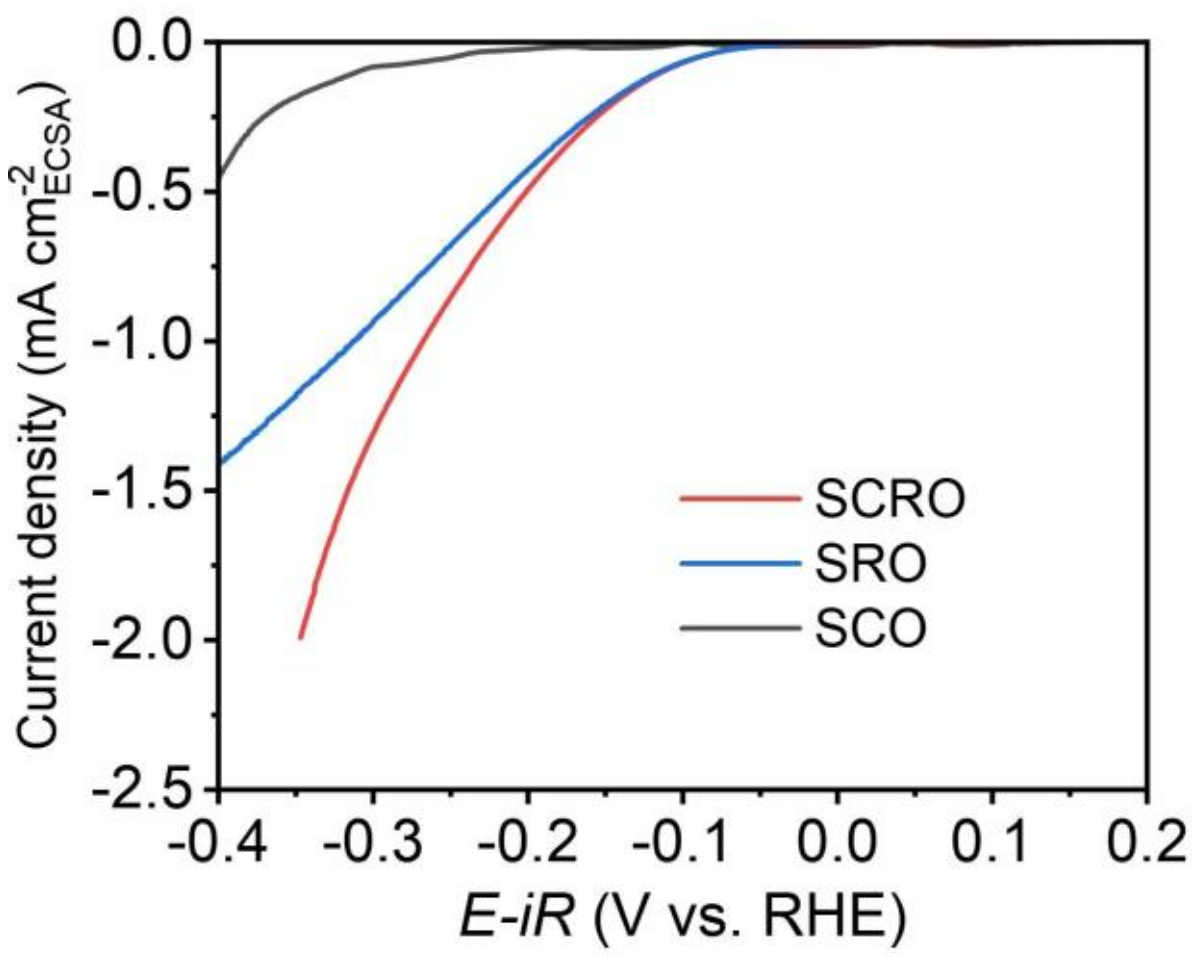

Figure R10 (also **Supplementary Figure 15**). Specific activity for HER normalized to ECSA of SCRO, SCO and SRO as a function of applied potentials.

Related data (**Supplementary Figs. 9&11&14&15**) and discussions have been provided in the revised manuscript as follows:

"To evaluate the intrinsic activity of SCRO, we calculated the specific activity by normalizing its apparent electrocatalytic activity to the real oxide surface area (ROSA) and the

*electrochemical surface area (ECSA). The ROSA and ECSA values of electrocatalysts were determined from double-layer Brunauer-Emmett-Teller (BET) method (**Supplementary Fig. 8)** and double-layer capacitance (C_{dl}) measurements (**Supplementary Fig. 9**), respectively. As shown in **Supplementary Figs. 10&11**, whether the apparent activity normalized to the ECSA or ROSA, the SCRO catalyst exhibits a much higher specific activity as compared with SRO and SCO."* (yellow-highlighted in **Line 15-22**, **Page 8**)

*"Besides, SCRO exhibits much higher specific activity and mass activity compared with SCO and SRO as well (**Fig. 2e and Supplementary Figs. 13-15**)."* (yellow-highlighted in **Line 34**, **Page 8, Line 1-2**, **Page 9**)

Comment 2: The authors attribute the enhanced performance to "super-exchange interactions" between Co^{3+} and Ru^{5+}. While the XAS and Bader charge analysis support charge redistribution, the direct link between super-exchange and catalytic activity is not fully established. The manuscript would benefit from a more explicit discussion of how super-exchange (e.g., ferromagnetic coupling) facilitates electron transfer or optimizes intermediate adsorption. DFT calculations comparing the electronic structure of SCRO with SCO and SRO, specifically in terms of d-band center or oxygen p-band center, would provide more concrete evidence.

Response to C2: We thank the reviewer for this valuable suggestion. For clarity, we separated this comment into two parts to answer:

1) As for the effect of super-exchange on electron transfer, we have given a more explicit discussion in the revised manuscript on the basis of prior analysis in the original manuscript. On the basis of previous reports, super-exchange effect within SCRO could give rise of **a short range Co^{3+}-O-Ru^{5+} ferromagnetic coupling according to G-K rule**, which is beneficial for better electron transport and conduction (*J. Phys. Condens. Matter* 2016, 28, 436005; *Small* 2019, 15, 1903120; *Nat. Commun.* 2020, 11, 5657). Especially for Co, HS Co^{3+} cations with a high spin state were endowed with **a narrower gap between e_g and t_{2g}** in Co 3*d* states, which could improve the electron conductivity (*Chem. Mater.* 2017, 29, 10534). Besides, from the perspective of crystal structure, the formation of monoclinic phase induced by super-exchange interactions in SCRO makes **the bond angle of Co-O-Ru closely approach to 180°**, which could minimize the local strain in SCRO and thus be

beneficial for better electron conduction (*Small* 2018, 61, 1803513; *ACS Appl. Energy Mater.* 2018, 1, 3342).

2) As for DFT calculations comparing the electronic structure, **as suggested by the reviewer**, **we have analyzed the oxygen *p*-band center** of SCRO, SCO, and SRO in the revised manuscript (**Figure R11**). Notably, the **O 2*p* band center of SCRO (-0.98 eV) is significantly closer to the Fermi level** than SRO (-2.1 eV) and SCO (-2.38 eV), suggesting its **significantly decreased oxide stability and improved electron transport capability**, which is **favorable for surface construction under electrocatalytic reaction conditions** (*Nat. Commun.* 2013, 4, 2439; *J. Am. Chem. Soc.* 2026,148,3128; *Angew. Chem. Int. Ed.* 2023, 62, e202215654; *Adv. Mater.* 2025, 37, e09664).

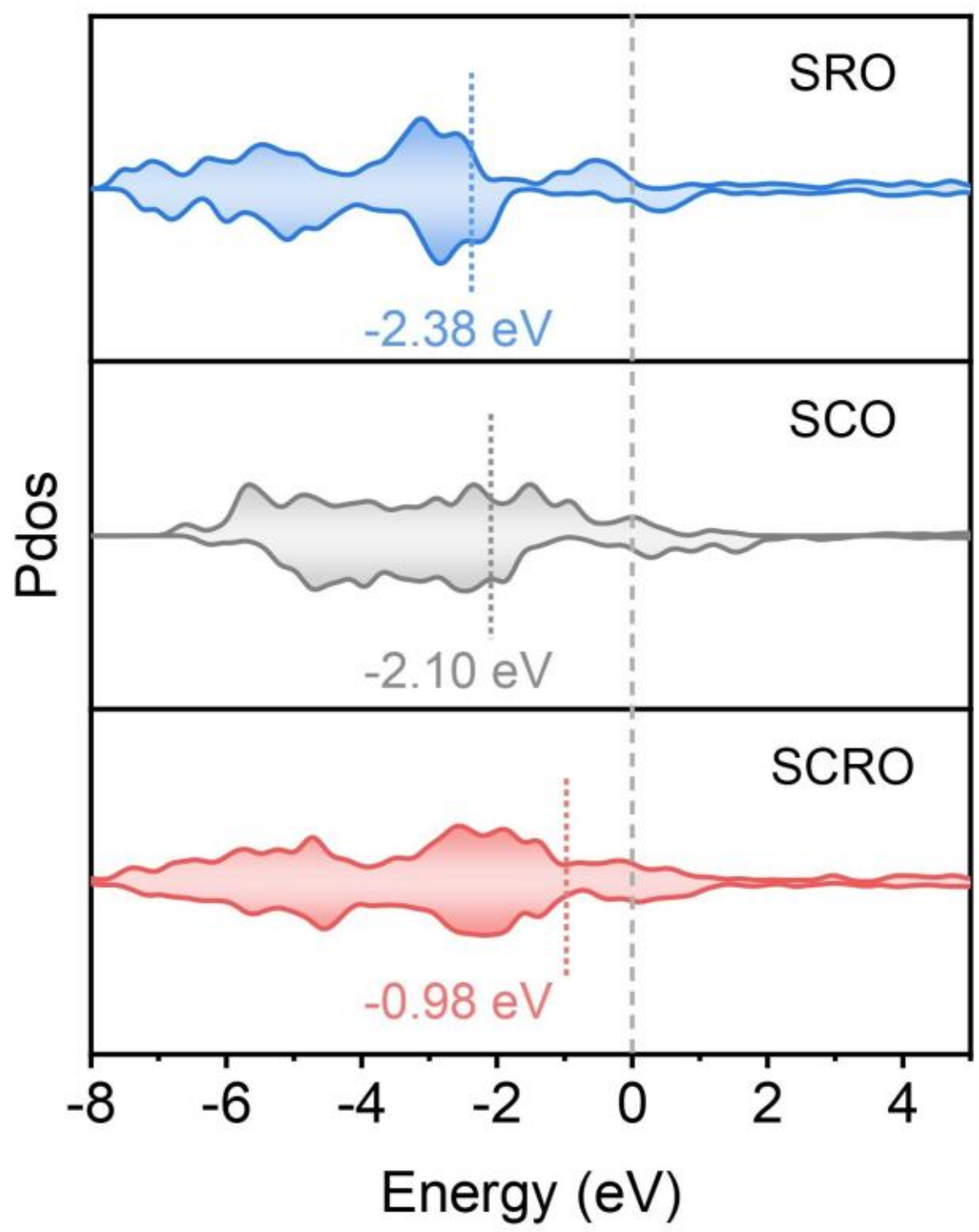

Figure R11 (also **Supplementary Figure 18**). PDOS of O 2*p* orbitals for SCRO, SRO, and SCO.

Related data (**Supplementary Fig. 18**) and discussions have been provided in the revised manuscript as follows:

"On the basis of previous reports, super-exchange effect within SCRO could give rise of a short range Co^{3+}-O-Ru^{5+} ferromagnetic coupling according to G-K rule, which is beneficial for better electron transport and conduction[31,36]*. Besides, the formation of monoclinic phase*

induced by super-exchange interactions in SCRO makes the bond angle of Co-O-Ru closely approach to 180°, which could minimize the local strain and thus be favorable for electron conduction as well[31]*."* (yellow-highlighted in **Line 34, Page 6, Line 1-5, Page 7**)

*"Besides, the O 2p band center of SCRO is much closer to the Fermi level than SRO and SCO (***Supplementary Fig. 18***), implying its significantly decreased oxide stability and improved electron transport capability, which is favorable for surface construction under electrocatalytic reaction conditions*[45, 46]*."* (yellow-highlighted in **Line 9-13, Page 10**)

Comment 3: The *in-situ* XAS experiments are a major strength of this work. However, the authors should discuss potential artifacts, such as beam-induced damage or changes in the electrolyte layer thickness during measurements. Additionally, the time resolution of the measurements (e.g., how long each spectrum took to acquire) should be reported, as this affects the interpretation of dynamic structural changes.

Response to C3: We thank the reviewer for raising this important point. For clarity, we separated this comment into three parts to answer:

(1) First, **we agree with the reviewer** that potential artifacts associated with *in-situ* XAS experiments, including beam-induced damage, variations in electrolyte thickness, and the time resolution of spectral acquisition, should be clarified to ensure a rigorous interpretation of dynamic structural evolution. With regard to possible beam-induced effects, the **in-air Co and Ru *K*-edge XANES spectra of SCRO** collected prior to the OER, HER and ORR measurements are **highly consistent** with each other **(Figure R12)**. No detectable spectral difference induced by X-ray beam was observed over three independent measurements, suggesting that **beam-induced reconstruction is negligible**. Therefore, the spectral evolution observed under reaction conditions is mainly attributed to the electrochemical process **rather than** the beam-induced damage.

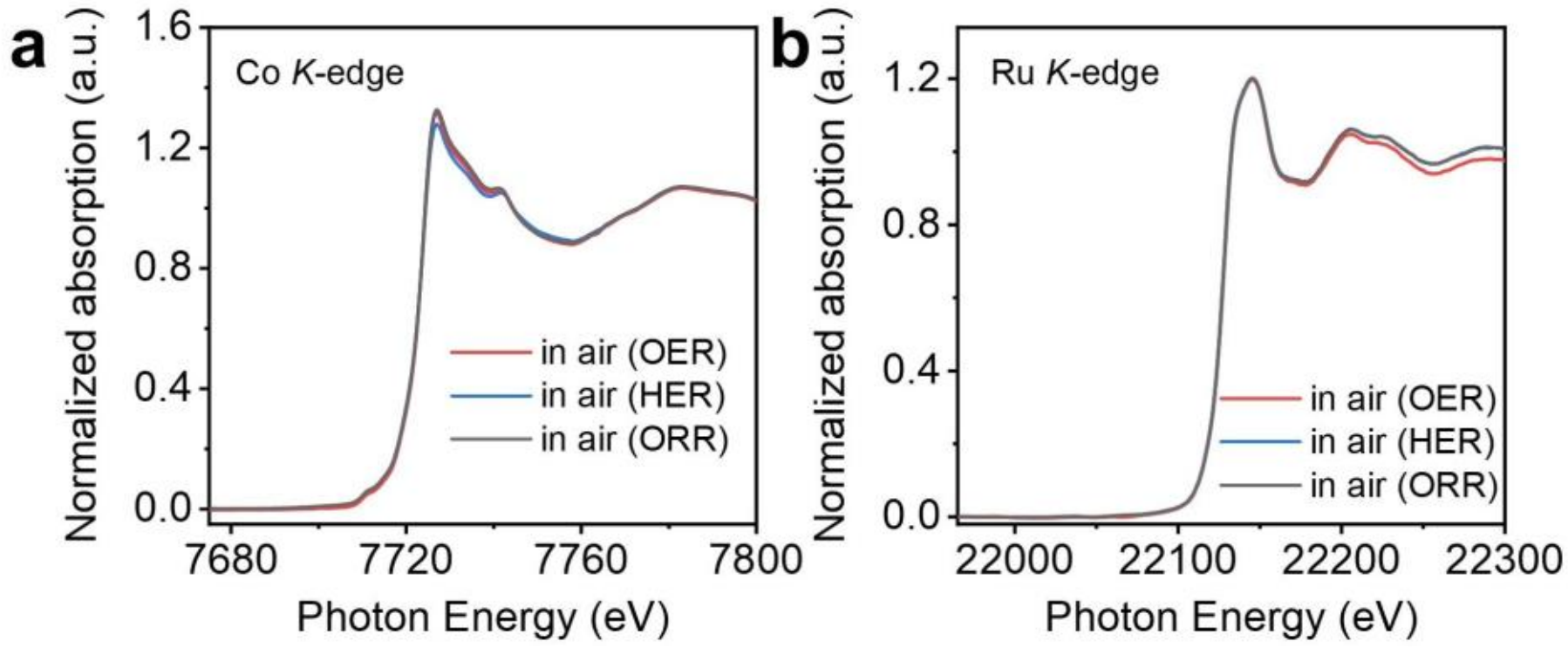

Figure R12. (a) In-air Co-*K* edge and (b) Ru-*K* edge XANES spectra of SCRO collected prior to the OER, HER and ORR *operando* electrocatalytic measurements.

(2) Second, the *in-situ* XAS measurements were performed at the Taiwan Photon Source (TPS) 44A beamline of the National Synchrotron Radiation Research Center (NSRRC) in Taiwan, **a quick-scanning XAS beamline** featuring high temporal resolution and sub-second acquisition capability, which is particularly suitable for **tracking fast redox processes and transient intermediates** during electrocatalysis. [*Synchrotron Radiat. News* 2026, 39, 4]. Such high temporal resolution and short acquisition times help **minimize potential artifacts arising from electrolyte-layer fluctuations during *operando* measurements.** Moreover, *in-situ* XAS has been widely used in recent studies to probe catalyst reconstruction in diverse electrocatalytic reactions (*e.g.*, OER, HER, ORR, CO_2RR), further **supporting the reliability of this technique for monitoring dynamic structural evolution in electrolyte-containing environments.** [*e.g.*, *Nat. Commun.* 2025, 16, 8827; *Nat. Commun.* 2024, 15, 3416; *Nat. Commun.* 2024, 15, 1675; *Nat. Commun.* 2024, 15, 55080; *Angew. Chem., Int. Ed.* 2025, 64, e202509063; *Adv. Energy Mater.* 2025, 15, 2404599; *Energy Environ. Sci.* 2025, 18, 7527; *Adv. Funct. Mater.* 2021, 31, 2104746, etc.]

(3) Third, regarding **time resolution of *in-situ* XAS experiments**, the TPS 44A beamline utilizes **a quick-scanning monochromator (Q-mono) system for real-time XAS data collection with one spectrum of 0.5 second**, enabling the monitoring of reactions with a time resolution down to the millisecond level. More details about *in-situ* XAS experiments have been added to the **Experimental Section** of the revised supporting information as follows:

"XAS data at the Co and Ru K-edges were collected at the Taiwan Photon Source (TPS)

44A beamline of the National Synchrotron Radiation Research Center (NSRRC) in Taiwan, which employs a quick-scanning monochromator (Q-mono) system for real-time XAS data collection with one spectrum of 0.5 second and provides high temporal resolution down to the millisecond level. In-situ XAS experiments were performed under the desired conditions with a custom-built operando XAS instrument."

Comment 4: The authors normalize OER and HER activity to the "real oxide surface area" (ROSA) determined by BET. For bulk oxide particles (micrometer-sized as shown in SEM), BET surface area may not accurately reflect the electrochemically accessible surface area, especially if the particles are non-porous. The authors should consider using electrochemical surface area (ECSA) methods (e.g., double-layer capacitance) to cross-validate the ROSA values. Alternatively, they should discuss the limitations of using BET for bulk materials.

Response to C4: We thank the reviewer for reiterating this important point. **As suggested by the reviewer**, we have added electrochemical surface area (ECSA) analysis in the revised manuscript by measuring the electrochemical double-layer capacitance (C_{dl}) of SCRO, SRO, and SCO from CV scans recorded in the non-faradaic potential region for OER and HER, respectively (**Figure R13&R15**). As shown in **Figure R14&R16**, after normalization to the ECSA, **SCRO still exhibits much higher specific activities for both OER and HER than SCO and SRO**, providing an electrochemical cross-validation of the BET-based normalization in the original manuscript.

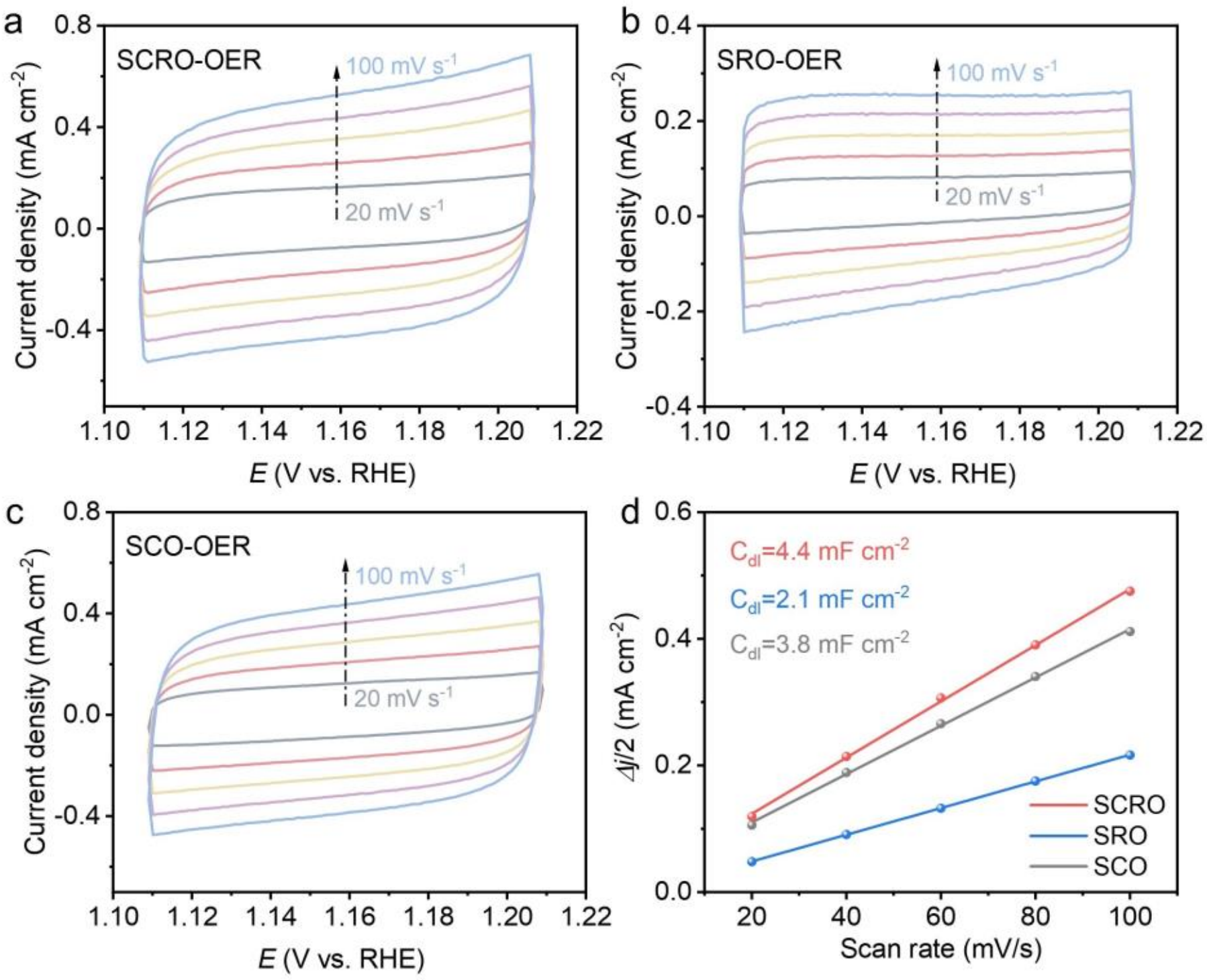

Figure R13 (also **Supplementary Figure 9**). Electrochemical CV scans of (a) SCRO, (b) SRO and (c) SCO in the non-faradaic potential region for OER. (d) Calculated double layers capacitances extracted from the linear fitting of the capacitive currents versus CV scan rates for SCRO, SRO and SCO.

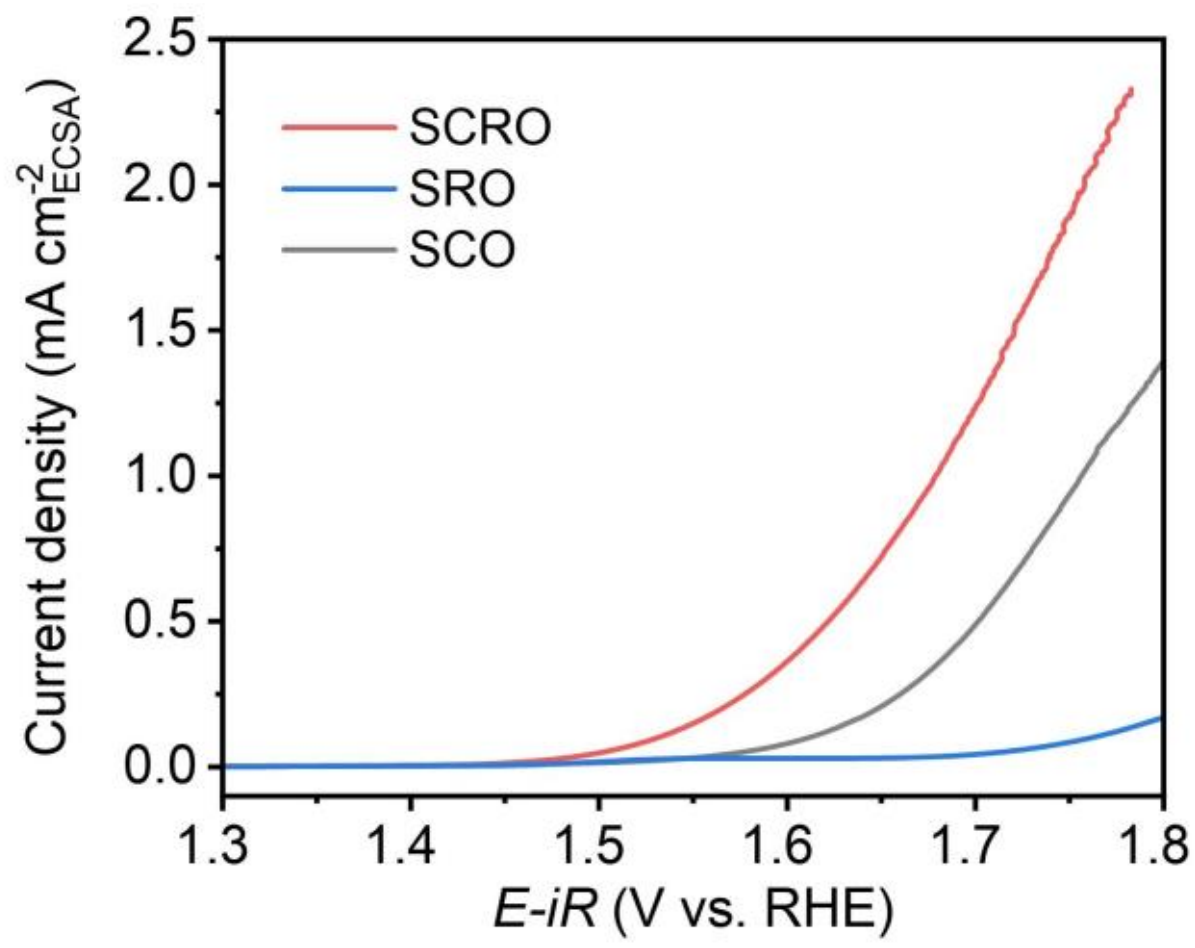

Figure R14 (also **Supplementary Figure 11**). Specific activity for OER normalized to ECSA of SCRO, SCO and SRO as a function of applied potentials.

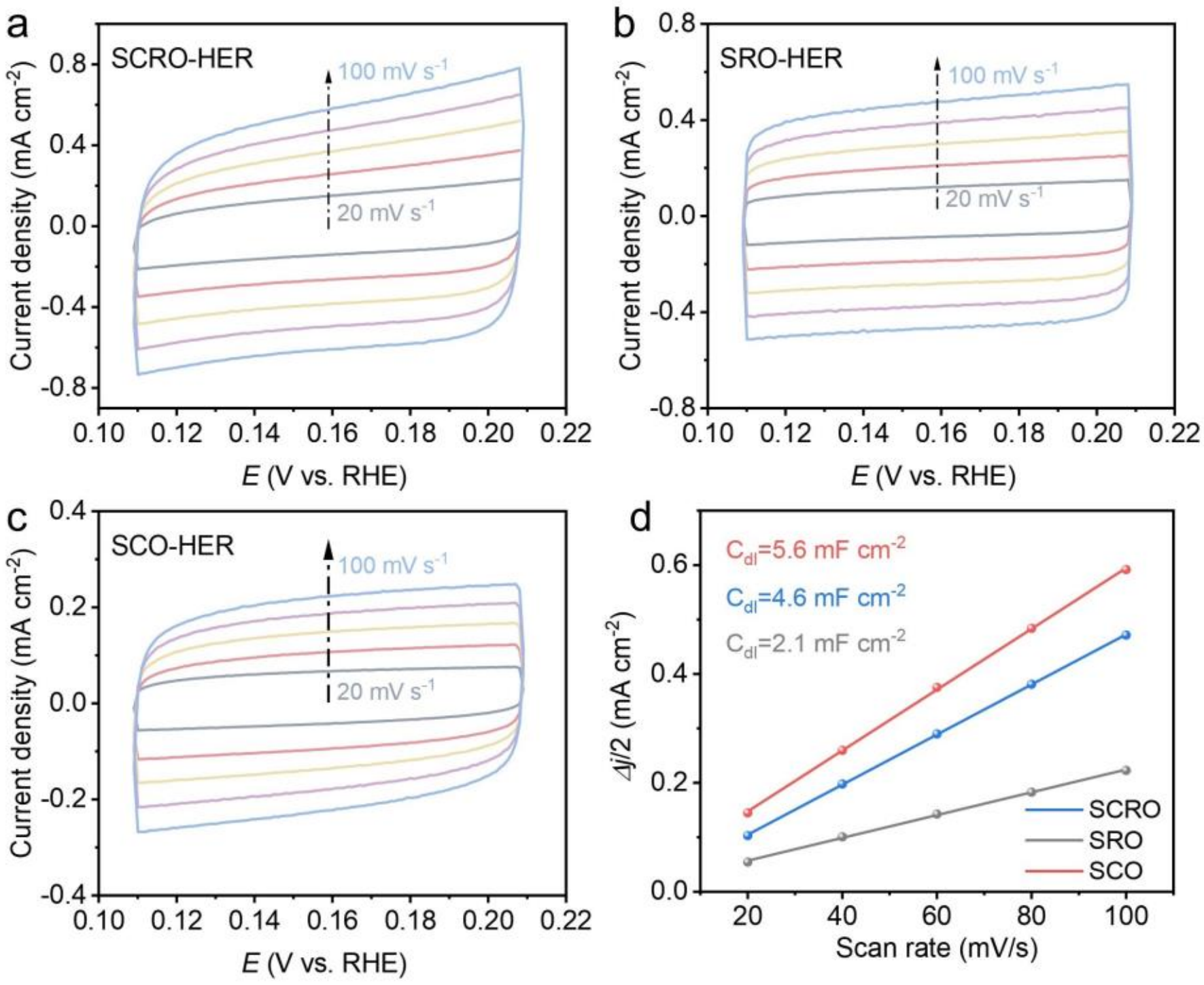

Figure R15 (also **Supplementary Figure 14).** Electrochemical CV scans of (a) SCRO, (b) SRO and (c) SCO in the non-faradaic potential region for HER. (d) Calculated double layers capacitances extracted from the linear fitting of the capacitive currents versus CV scan rates for SCRO, SRO and SCO.

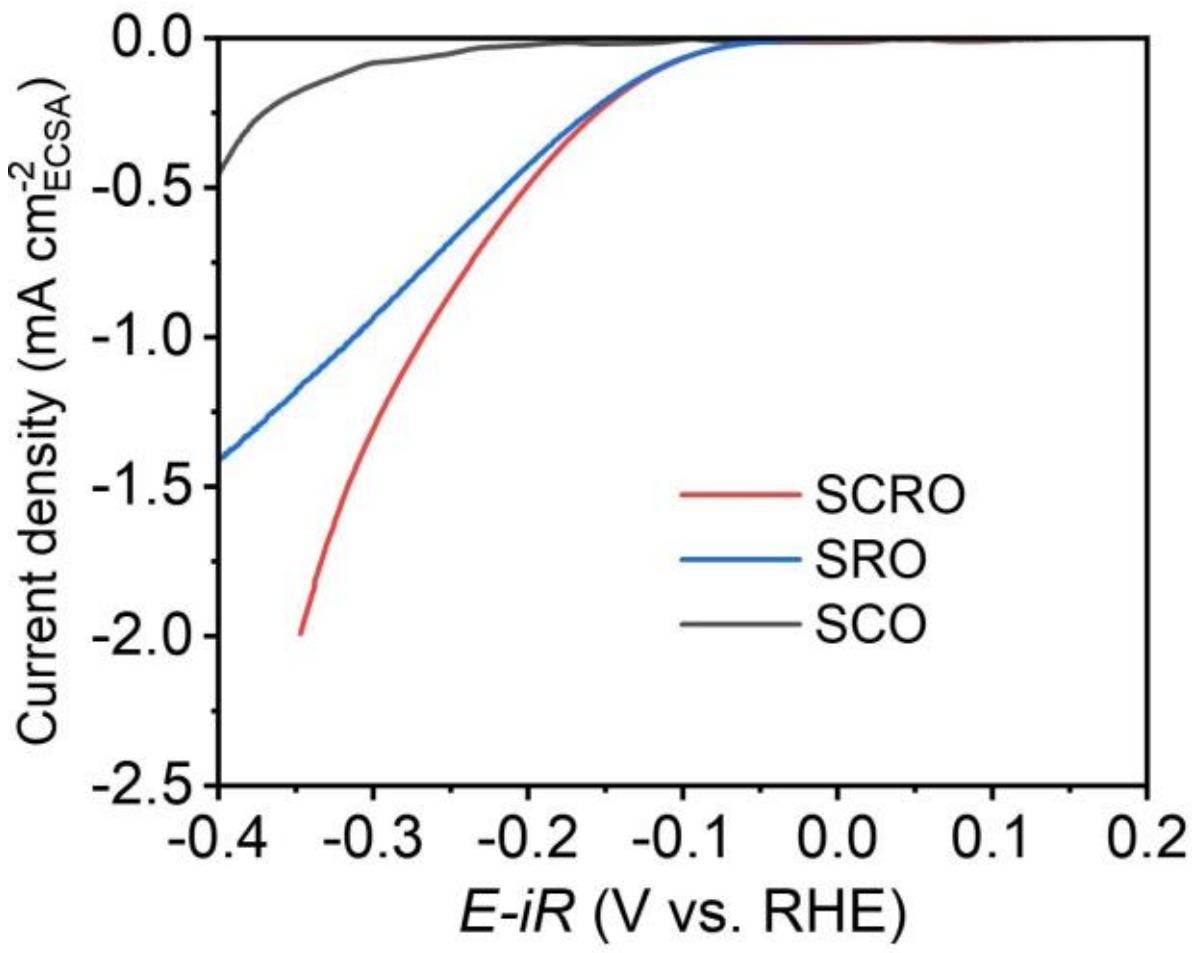

Figure R16 (also **Supplementary Figure 15).** Specific activity for HER normalized to ECSA of SCRO, SCO and SRO as a function of applied potentials.

Related data (**Supplementary Figs. 9&11&14&15**) and discussions have been provided in the revised manuscript as follows:

"To evaluate the intrinsic activity of SCRO, we calculated the specific activity by normalizing its apparent electrocatalytic activity to the real oxide surface area (ROSA) and the

*electrochemical surface area (ECSA). The ROSA and ECSA values of electrocatalysts were determined from double-layer Brunauer-Emmett-Teller (BET) method (**Supplementary Fig. 8)** and double-layer capacitance (C_{dl}) measurements (**Supplementary Fig. 9**), respectively. As shown in **Supplementary Figs. 10&11**, whether the apparent activity normalized to the ECSA or ROSA, the SCRO catalyst exhibits a much higher specific activity as compared with SRO and SCO."* (yellow-highlighted in **Line 15-22**, **Page 8**)

*"Besides, SCRO exhibits much higher specific activity and mass activity compared with SCO and SRO as well (**Fig. 2e and Supplementary Figs. 13-15**)."* (yellow-highlighted in **Line 34**, **Page 8, Line 1-2**, **Page 9**)

Comment 5: The SCRO-based liquid ZAB exhibits exceptional stability for 1000 h at 5 mA cm^{-2}. While impressive, this current density is relatively low for practical applications. The authors should provide cycling data at higher current densities (e.g., 10 or 20 mA cm^{-2}) to demonstrate the catalyst's durability under more demanding conditions. This would also allow for a fairer comparison with many literature reports that test at 10 mA cm^{-2}.

Response to C5: We thank the reviewer for this insightful suggestion. **As suggested by the reviewer**, the durability of liquid ZAB at **a higher current density of 10 mA cm^{-2}** has been further evaluated in the revised manuscript (**Figure R17**), and the device can still operate reliably for **300 h** under this more demanding condition, which is comparable to many literature reports, **demonstrating its potential in practical application.**

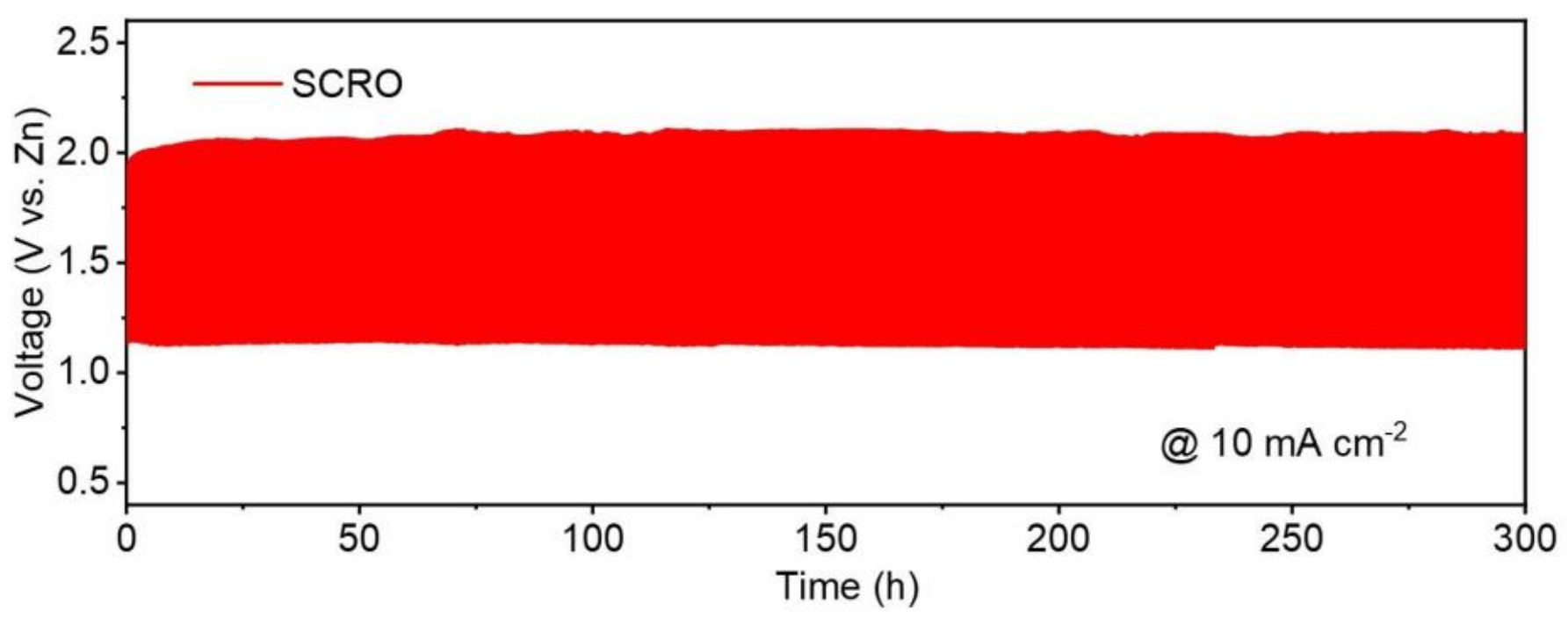

Figure R17 (also **Supplementary Figure 30).** Charge-discharge cycling curves at 10 mA cm^{-2} of SCRO-based liquid ZAB.

Related data (**Supplementary Fig. 30**) and discussion (yellow-highlighted in **Line 8-10**, **Page 15**) have been provided in the revised manuscript as follows:

*"Even under a higher current density of 10 mA cm^{-2}, SCRO-based liquid ZAB still maintains stable operation for 300 h (1800 cycles) without noticeable decay (**Supplementary Fig. 30**)."*

Comment 6: The AEMWE achieves 1 A cm^{-2} at 1.90 V, which is commendable. The breakdown of overvoltage contributions (η_{Ohmic}, $\eta_{Kinetic}$, $\eta_{Transport}$) is insightful. However, the method for calculating $\eta_{Kinetic}$ (using a "linear approximation in the Tafel region") is not clearly described. The authors should specify the potential range used for the Tafel fit and provide the Tafel plots for the full cell. Additionally, the stability test at 0.5 A cm^{-2} for 100 h shows a decay rate of 0.8 mV h^{-1}. While low, the authors should discuss whether this decay is due to catalyst degradation, membrane degradation, or other factors, and provide post-mortem characterization of the electrodes.

Response to C6: We thank the reviewer for this valuable comment. For clarity, we separated this comment into two parts to answer:

(1) In the revised manuscript, **we have clarified the method used to calculate the breakdown of overvoltage contributions** in the **Experimental Section** of the revised supporting information. Briefly, the kinetic overvoltage was analyzed by correcting E_{cell} from the polarization curves for ohmic resistance, yielding $E_{ohmic\text{-}corrected}$. From the corrected polarization curves, **the Tafel slope in the low current density region** was determined. Extrapolation of the fitted Tafel line to the maximum current density allowed for the determination of $\eta_{kinetic}$. Specifically, for SCRO || SCRO-based AEMWE, the Tafel fitting was performed **in the low current density region corresponding to an ohmic-corrected overpotential range of 0.22-0.32 V** with a fitted relation of η= 0.392 + 0.103log$j$, from which $\eta_{kinetic}$ was obtained over the full current-density range (**Figure R18**).

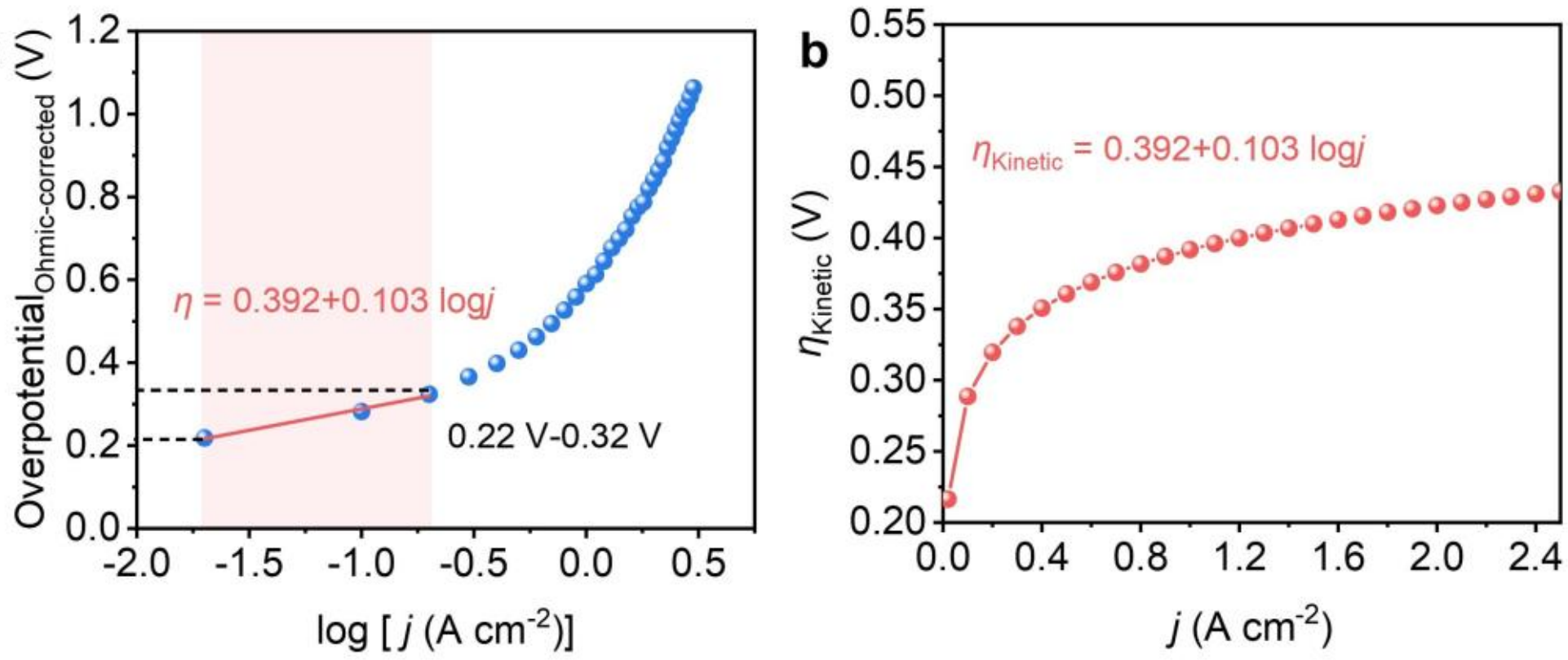

Figure R18 (a) Full-cell Tafel plot of the SCRO || SCRO-based AEMWE derived from the ohmic-corrected polarization curve, showing the fitting region (0.22-0.32 V) used for kinetic-overvoltage analysis. (b) Kinetic overvoltage of SCRO || SCRO-based AEMWE.

Related discussions have been provided in the **Supplementary Notes** of the revised Supporting Information, as follows:

"The overvoltage was divided into three resistances: ohmic overvoltage (η_{Ohmic}), kinetic overvoltage ($\eta_{Kinetic}$) and transport overvoltage ($\eta_{Transport}$). The value of η_{Ohmic} was derived from electrochemical impedance spectroscopy results, as described by the Equation (3):

$$\eta_{Ohmic} = j \times HFR\ (V) \tag{3}$$

where j is current density (A cm^{-2}), and HFR is the high frequency resistance (Ω cm^{-2}) measured at the current density of 0.5 A cm^{-2}.

The overvoltage contributions were further analyzed by correcting E_{cell} from polarization curves for ohmic resistance, yielding $E_{ohmic\text{-}corrected}$. From the corrected polarization curve, the Tafel slope in the low current density region was determined. Extrapolation of the fitted Tafel line to the maximum current density allowed for the determination of $\eta_{kinetic}$ using Equation (4):

$$\eta_{Kinetic} = a + b \times \log j\ (V) \tag{4}$$

where j is the applied current density.

The value of $\eta_{Transport}$ was obtained by Equation (5):

$$\eta_{Transport} = E_{cell} - \eta_{Ohmic} - \eta_{Kinetic} - E_{eq}\ (V) \tag{5}$$

where E_{eq} is the thermodynamic equilibrium potential of 1.23 V."

(2) **As suggested by the reviewer**, we have **added post-mortem characterization of the electrodes** after the stability test at 0.5 A cm^{-2} in the revised manuscript. As shown in **Figure**

R19, post-mortem SEM characterization of the electrode after durability testing **reveals partial delamination of the catalyst layer** after 100 h of operation. Besides, ICP analysis of the electrolyte shows **a progressive increase in the concentration of dissolved active metal species (Co and Ru)** with testing time (**Figure R20**), further indicating the catalyst's dissolution during electrolysis. Taken together, the observed voltage decay rate of 0.8 mV h^{-1} may **originate from gradual catalyst dissolution, accompanied by partial delamination of the catalyst layer.**

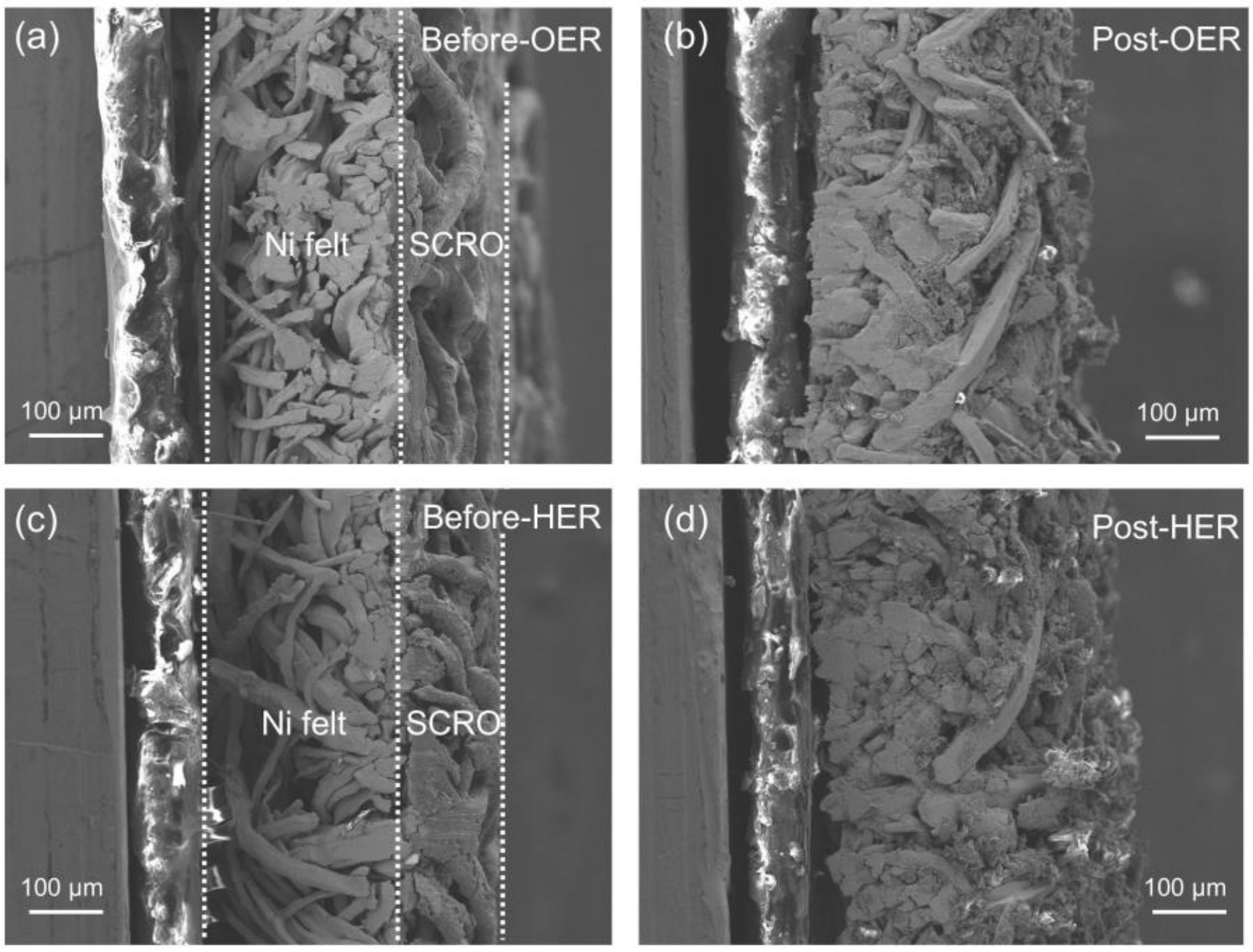

Figure R20 (also **Supplementary Figure 39).** Cross-sectional SEM images of the SCRO-coated Ni felt electrode before and after the AEMWE durability test: (a) pristine electrode before OER, (b) post-OER electrode, (c) pristine electrode before HER, and (d) post-HER electrode.

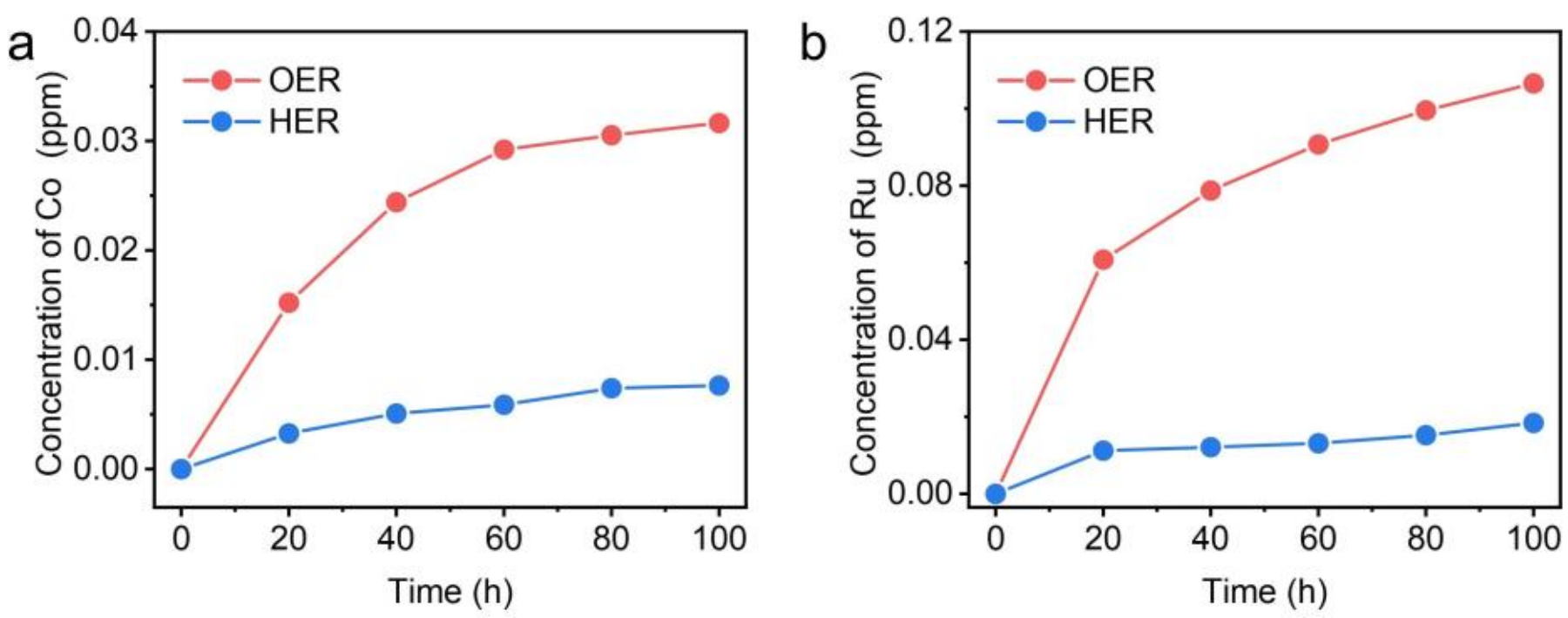

Figure R20 (also **Supplementary Figure 40).** Dissolution concentrations of (a) Co and (b) Ru ions in the electrolyte during OER and HER durability tests of the AEMWE at different time

intervals of 20 h, 40 h, 60 h, 80 h and 100 h.

Related data (**Supplementary Figs. 39&40**) and discussion (yellow-highlighted in **Line 12-15**, **Page 19**) have been provided in the revised manuscript as follows:

*"As evidenced by post-mortem SEM and ICP analyses (***Supplementary Figs. 39&40***), the voltage decay of SCRO-based AEMWE may be attributed to gradual catalyst dissolution accompanied by partial delamination of the catalyst layer during long-term operation."*

Reviewer #3

GENERAL COMMENTS: This manuscript reports a single-phase $Sr_2CoRuO_{6-\delta}$ double perovskite with super-exchange double perovskite structure as highly efficient trifunctional electrocatalyst for OER, HER and ORR simultaneously. Combining comprehensive in-situ techniques, surface reconstruction behaviors of $Sr_2CoRuO_{6-\delta}$ under varied reactions were systematically investigated and true active components were accurately identified. When used in practical applications, $Sr_2CoRuO_{6-\delta}$ shows multi-condition adaptability for Zn-air battery and water electrolysis with attractive performance parameters. Overall, the results are impressive where experiments were conducted with rigor, and the manuscript was well organized. I feel that this is an interesting and timely paper in the field of developing multifunctional electrocatalysts for emerging energy conversion and storage technologies, and I recommend for publications after minor revisions.

Response: We very much appreciate the **Reviewer #3's highly positive comments** (*i.e.*, **the results are impressive where experiments were conducted with rigor, and the manuscript was well organized**) and **recommendation for publication after minor revisions**. Our responses to his/her comments per point are as follows.

Comment 1: TEM evidence for surface reconstruction behavior remains somewhat limited, as only a single image was presented for each reconstructed state. The authors should provide clear overview images of the reconstructed particles, together with additional TEM images acquired from multiple representative regions.

Response to C1: We thank the reviewer for this valuable suggestion. As suggested by the reviewer, we have added **additional TEM images** after OER, HER and ORR at other zones in the revised manuscript (**Figure R21-23**), **further confirming the real active components of SCRO under different reaction conditions.**

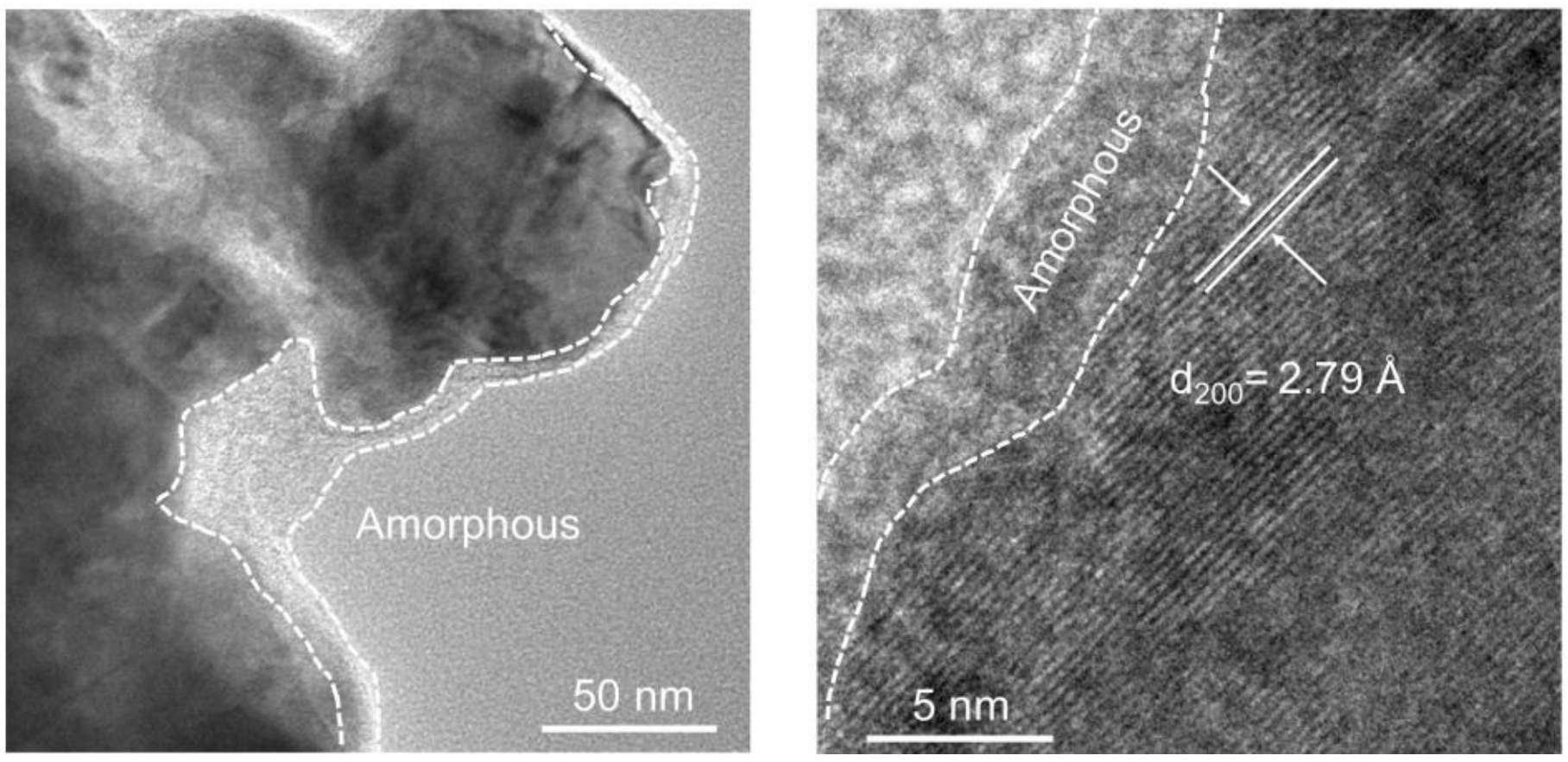

Figure R21 (also **Supplementary Figure 19**). HRTEM images of SCMO after OER at other zone.

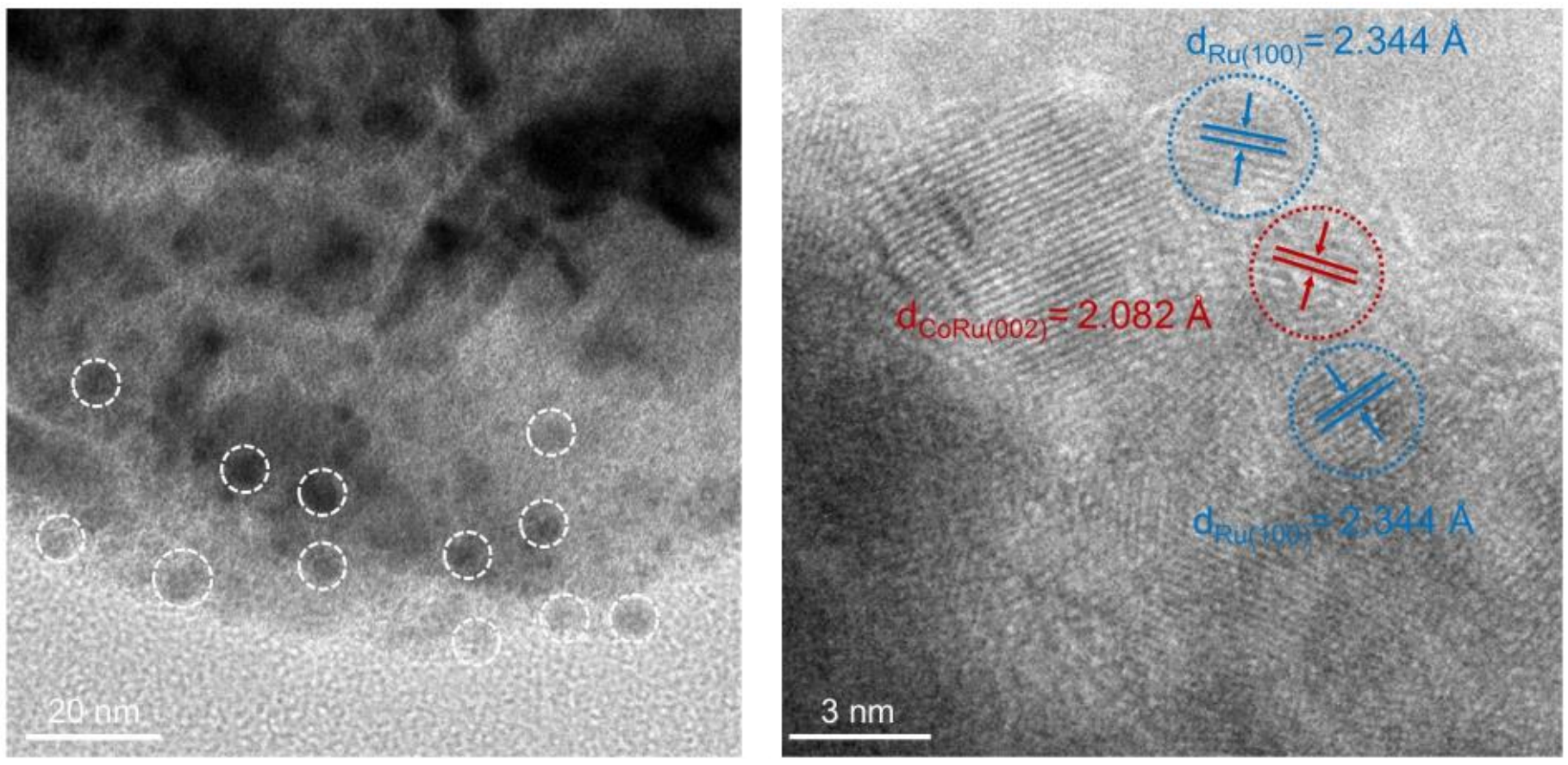

Figure R22 (also **Supplementary Figure 22**). HRTEM images of SCMO after HER at other zone.

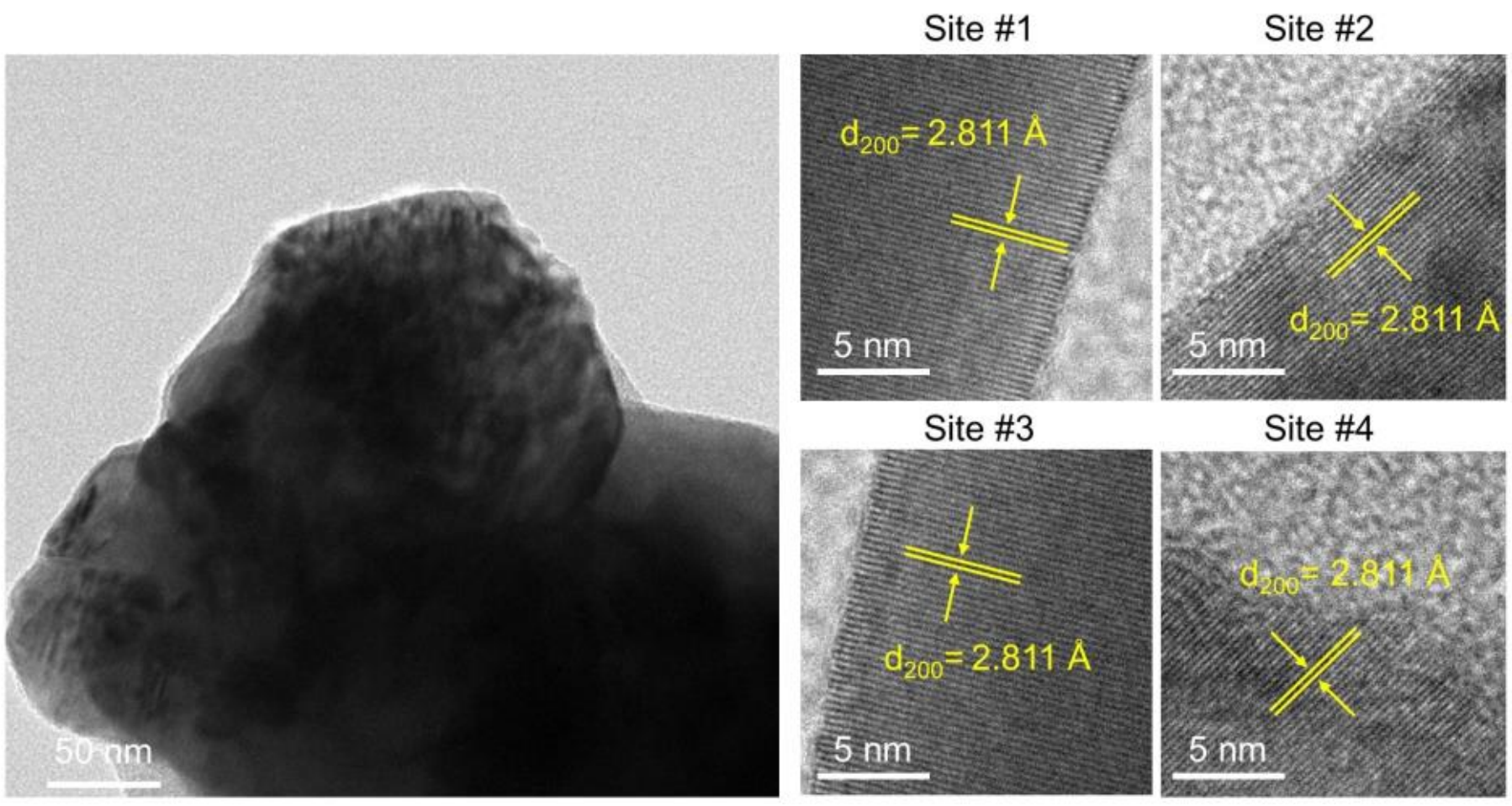

Figure R23 (also **Supplementary Figure 25**). HRTEM images of SCMO after ORR at other zone.

Related data (**Supplementary Figs. 19&22&25**) and discussions have been provided in the revised manuscript as follows:

*"Consistent with the results of in-situ XRD, HRTEM images obtained after the OER provide a direct reflection of reconstructed surface amorphous layer with approximately 5 nm thickness in SCRO (**Fig. 3b** and **Supplementary Fig. 19**)."* (yellow-highlighted in **Line 18-20**, **Page 10**)

*"Besides, on the basis of HRTEM examination, Ru metal and CoRu alloy NPs with particle size about 5 nm were directly seen on the surface of post-HER SCRO (**Fig. 4b** and **Supplementary Fig. 22**)."* (yellow-highlighted in **Line 11-13**, **Page 12**)

*"Also, HRTEM images obtained after ORR directly reflects the lattice expansion in post-ORR SCRO (**Fig. 5b** and **Supplementary Fig. 25**)."* (yellow-highlighted in **Line 13-14**, **Page 13**)

Comment 2: The rate performance data of the SCRO-based ZAB device indicate good reversibility, as the discharge voltage recovers to the initial level after the current density is increased to 20 mA cm^{-2} and subsequently switched back to 1 mA cm^{-2}. Nevertheless, a maximum current density of 20 mA cm^{-2} may not be sufficiently demanding to fully support the claimed reversibility. The authors are encouraged to extend the rate test to higher current densities, which would allow a more rigorous assessment of device performance under harsher operating conditions.

Response to C2: We thank the reviewer for this insightful suggestion. First, we **agree with** the reviewer that a maximum current density of 20 mA cm^{-2} is insufficient to support the claimed

reversibility. As suggested by the reviewer, we have extended the rate performance test of the SCRO-based ZAB device to a higher current density of **50 mA cm^{-2}** (**Figure R24)**, which **can still return to the initial level** when the current density gets back to 1 mA cm^{-2}, demonstrating its **distinguished reversibility**.

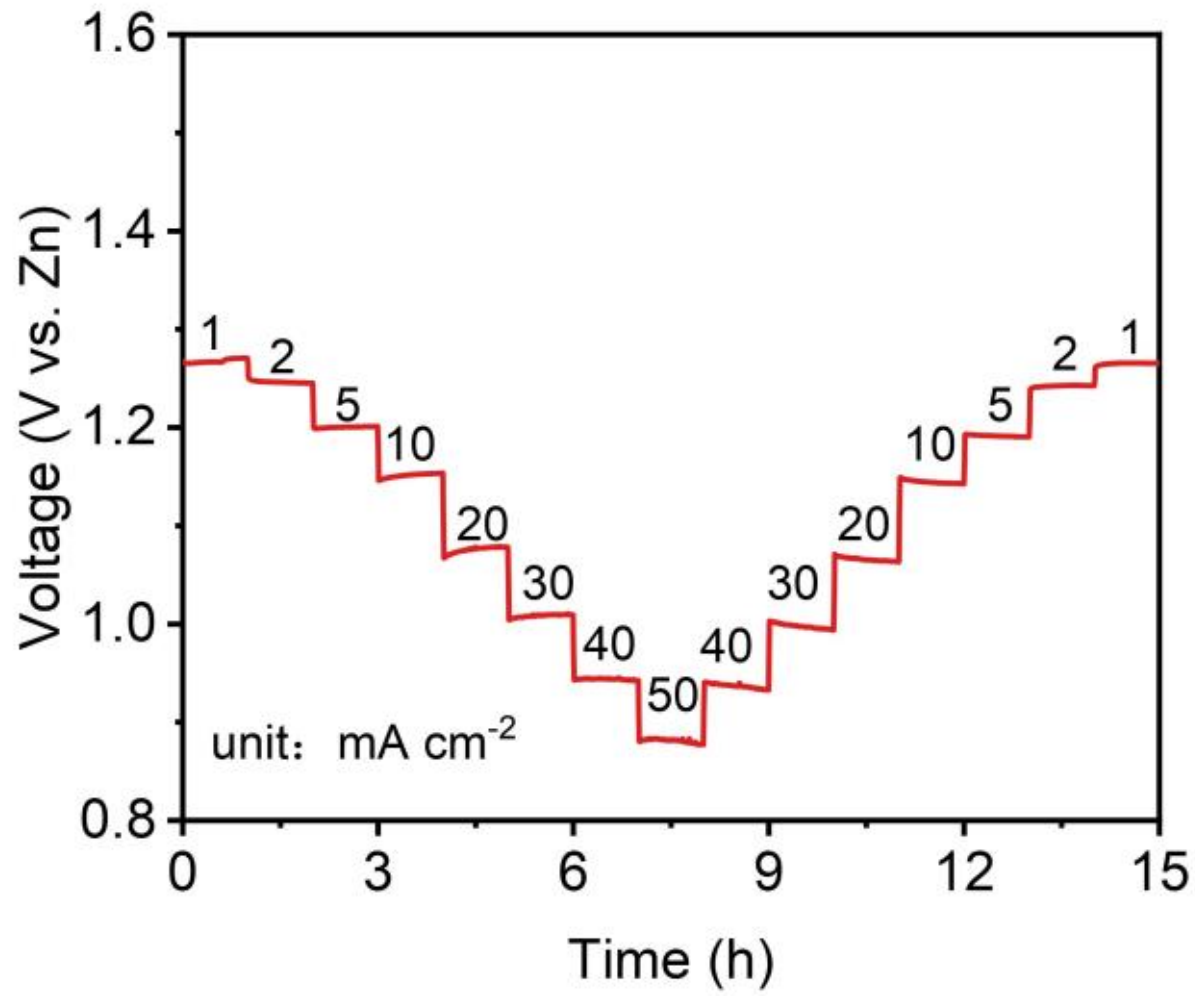

Figure R24 (also revised **Supplementary Figure 29**). Galvanostatic discharge curves at different current densities (1, 2, 5, 10, 20, 30, 40, 50 mA cm^{-2}).

Related data (**Supplementary Fig. 29**) and discussion (yellow-highlighted in **Lin 33-34, Page 14, Line 1-2, Page 15**) have been provided in the revised manuscript as follows:

*"According to the rate performance of SCRO-based ZAB device, the discharge voltage returns to the initial level when the current density gets back to 1 mA cm^{-2}, suggesting the excellent rate performance and distinguished reversibility of SCRO (**Supplementary Fig. 29**)."*

Comment 3: In Figure 6i, the demonstration of using flexible Zn-air battery to charge a mobile phone is interesting and effectively highlights the practical potential of the device. To further strengthen this result, the authors should provide additional experimental details, such as the working voltage of two flexible Zn-air batteries connected in series.

Response to C3: We thank the reviewer for this valuable suggestion. In the revised manuscript, **we have added additional experimental details** for the demonstration of charging a mobile phone using the flexible Zn-air battery. Specifically, two flexible Zn-air batteries connected in series delivered an open-circuit voltage of **2.55 V (Figure R25)**, and the series output was subsequently connected to a DC-DC boost converter to provide a regulated output for mobile-

phone charging.

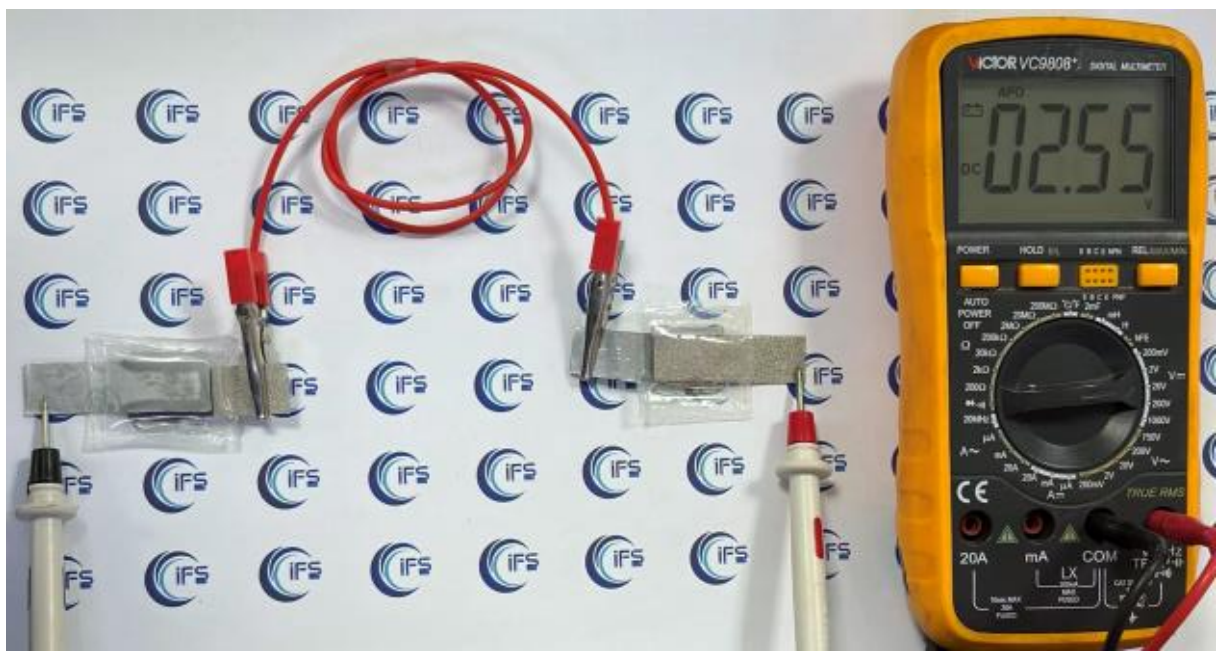

Figure R25 (also revised **Supplementary Figure 37**). Digital image of solid-state flexible ZAB connected in series showing working voltage of 2.55 V.

Related data (**Supplementary Fig. 37**) and discussion (yellow-highlighted in **Lin 27-30**, **Page 17**) have been provided in the revised manuscript.

"*With such outstanding performance, the solid-state flexible ZAB is able to power a mobile phone using two cells connected in series (**Fig. 6i**), which exhibit an open-circuit voltage of 2.55 V (**Supplementary Fig. 37**), demonstrating great potential of SCRO-based solid-state ZABs as a flexible energy supply.*"

Additional experimental details have also been included in the **Experimental Section** of the revised Supporting Information, as follows:

"*For the mobile-phone charging demonstration, two solid-state flexible Zn-air batteries were connected in series to deliver an open-circuit voltage of 2.55 V, and the output was further boosted to a regulated 5.0 V through a DC-DC converter for charging the mobile phone.*"

Comment 4: For AEMWE tests, catalyst powders typically face challenges such as peeling or detachment from the electrode surface during vigorous gas evolution. The authors are encouraged to provide more experimental details to clarify how such issues were mitigated or avoided in the present device configuration.

Response to C4: We thank the reviewer for raising this important question. We agree with the reviewer that powder catalysts peeling off from the electrode during gas evolution is one critical issue for the stability in AEMWE devices. To address or avoided this issue, we are devoted to perform mainly from the following three aspects: **1) Optimizing the catalyst ink recipe**. Catalyst ink recipe for the membrane electrode assembly (MEA) with **proper ratio** of DI water,

isopropyl alcohol and ionomer solution (5 wt%) was optimized, and especially **the proper amount of ionomer solution** is the key to reducing the catalyst peel off from the supporting substrate while keeping the optimized device performance. **2) Choosing the Ni felt as the substrate material for supporting the catalysts**. Some common substrate materials (*e.g.*, Ni felt, Ni foam and carbon paper and carbon cloth) were investigated in AEMWE devices, and it was found that the Ni felt-supported catalysts have the **best stability** possibly due to **enhanced strength and toughness** preventing the catalysts from breaking and falling off, as well as **proper porosity** benefiting for gas release. **3) Adopting the catalyst coated substrate (CCS) technology and optimizing the preparation method**. For the AEMEC device, the CCS construction is **beneficial for enhanced bubble release capability** as compared to the catalyst coated membrane (CCM) construction. Besides, catalysts were spray-deposited onto the substrates by **professional ultrasonic spraying instrument** instead of manual spray gun with proper loadings, guaranteeing the **uniform distribution** of catalysts and **enhancing the catalyst layer adhesion**. All these two conditions can improve the stability of AEMEC device with addressing the issue of catalyst peeling off.

Comment 5: A labeling inconsistency appears to be present in Figure 6. The figure caption uses "20% Pt/ +RuO_2", while the main text refers to the benchmark catalyst as "20% Pt/C+RuO_2". The authors should check and standardize this notation throughout the manuscript for clarity.

Response to C5: We thank the reviewer for pointing this out. We **apologize for our negligence in wrongly labeling** the benchmark catalyst 20% Pt/C +RuO_2 in Figure 6 caption. In the revised manuscript, the notation in the **Figure 6** caption **has been corrected**, and the benchmark catalyst has been consistently standardized as **20% Pt/C+RuO_2** throughout the manuscript for clarity.

Comment 6: Atomic site occupancy parameters for SCRO obtained from XRD structure refinement is suggested to be disclosed. Additionally, a comprehensive description of the data analysis protocol for quantifying Co oxidation state evolution via *in-situ* XANES spectroscopy under operando reaction conditions is required.

Response to C6: We thank the reviewer for these valuable suggestions. For clarity, we

separated this comment into two parts to answer:

(1) As suggested by the reviewer, the atomic site occupancy parameters of SCRO obtained from XRD structure refinement has been included in **Table R1**.

Table R1 (also **Supplementary Table 2**). Atomic site parameters of SCRO obtained from Rietveld refinement of the XRD data.

Atom	x	y	z	Occupancy
Sr1	0.00000	0.48978	0.25000	1.0000
Co1	0.00000	0.00000	0.00000	0.4965
Ru1	0.00000	0.00000	0.00000	0.5035
O1	0.00000	0.04012	0.25000	0.9852
O2	0.22807	0.76177	0.03923	0.9838

Related data (**Supplementary Table 2**) and discussions (highlighted in yellow in **Lin 1-4**, **Page 5**) have been provided in the revised manuscript:

*"X-ray diffraction (XRD) and corresponding Rietveld refinement confirm that SCRO was successfully prepared comprising a pure monoclinic structure in the space group I2/c without any impurity phases (**Fig. 1b and Supplementary Tables 1&2**)."*

(2) With regard to the **data analysis protocol** for operando *in-situ* XANES, we have added a more comprehensive description in the **Supplementary Notes** of the revised Supporting Information, as follows:

"All XAS data were analyzed using the standard program Demeter. To quantify the accurate valence states of Co and Ru, standard reference compounds with well-defined oxidation states were provided to establish calibration curves between the absorption-edge energy and the oxidation state. The edge energies were extracted at fixed normalized intensities of 0.8 for Co and 0.6 for Ru, respectively. Then, the valence states of Co and Ru in SCRO under operando reaction conditions were determined by comparing the edge energies of the in-situ XANES spectra with the corresponding calibration curves."

Comment 7: In Figure 2d&f, 10 mA cm^{-2} should be -10 mA cm^{-2} and please modify. The figures in the manuscript are of low resolution. Please provide clearer ones.

Response to C7: We thank the reviewer for pointing out these issues and sincerely apologize for our oversight. In the revised manuscript, the current density labels in **Figure 2d&f** have been **corrected from 10 mA cm^{-2} to -10 mA cm^{-2}** (**Figure R26**)**.** In addition, as suggested by the reviewer, the figures in the manuscript have been replaced with clearer, higher resolution versions.

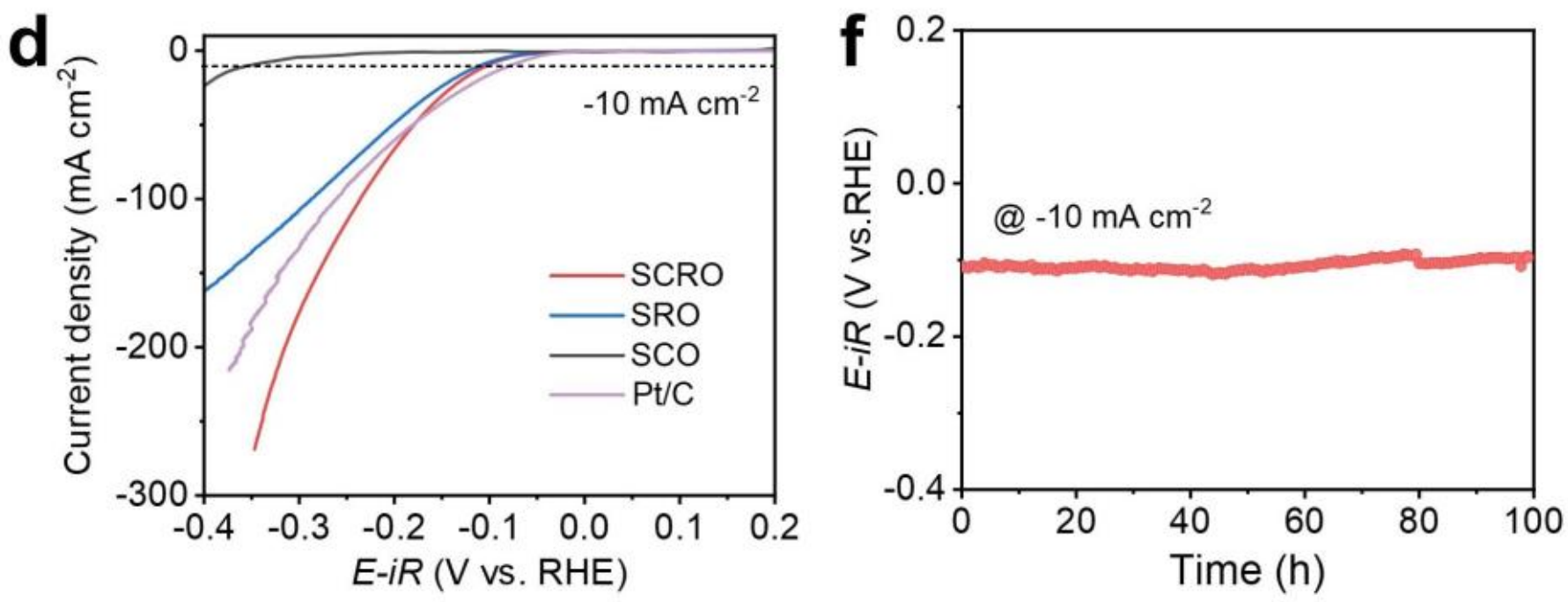

Figure R26 (also revised **Figure 2d&f**)**.** (d) HER polarization curves of SCRO, SCO, and SRO in 1 M KOH. (f) Chronopotentiometry response of SCRO at a constant HER current density of -10 mA cm^{-2}.

Point-to-Point Responses to Reviewers' Comments and Suggestions

To address the specific concern/point clearly, we have separated the referees' comments into question areas and answered them in turn as follows.

Reviewer #1

GENERAL COMMENTS: The revisions solved my comments.

Response: We sincerely thank Reviewer #1 for the positive assessment of our revised manuscript. We are pleased that our revisions have addressed the reviewer's concerns.

Reviewer #2

GENERAL COMMENTS: The author has addressed and revised each of my comments, and I agree and appreciate the effort. However, there is still a minor issue: in Supporting Information Figure 14d, the legend for the curve does not match the results in Supporting Information Figure14 (a-c). Please make the necessary corrections. Once the changes have been made, we recommend accepting them.

Response: We thank Reviewer #2 for the careful evaluation of our revised manuscript and for the positive comments. We also appreciate the reviewer for pointing out the remaining issue in Supporting Information Figure 14d. During the revision of the Supplementary Information, this figure was renumbered as Supplementary Fig. 17 in the final version. In response, we have corrected the legend in **Supplementary Fig. 17d**, which is consistent with the results shown in **Supporting Information Fig. 17a-c**. The revised figure has been updated in the Supporting Information.

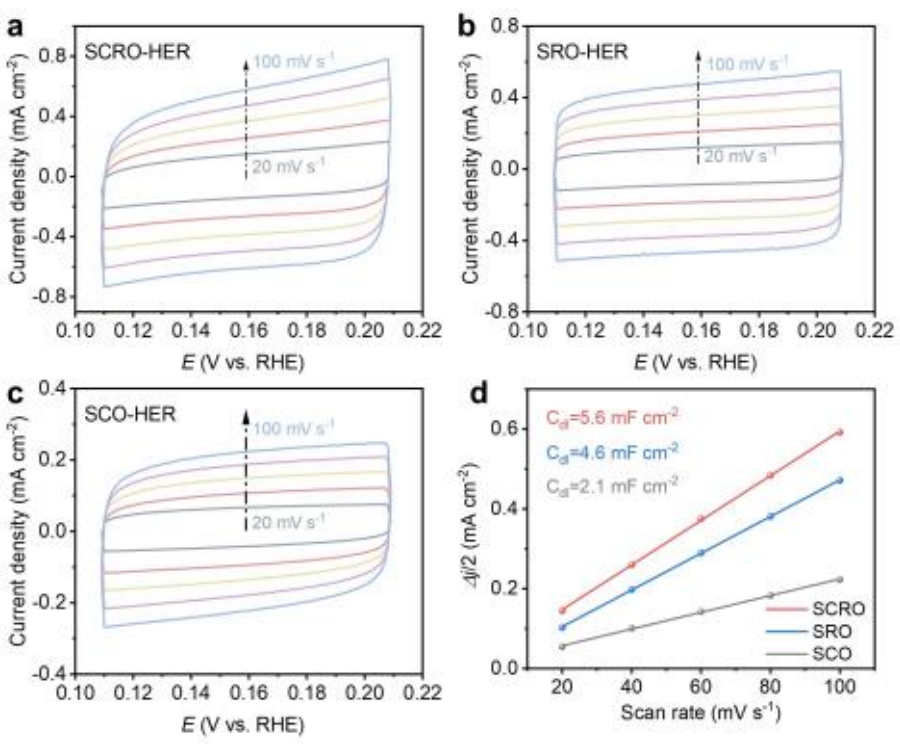

Figure R1 (also **Supplementary Figure 17).** Electrochemical CV scans of (a) SCRO, (b) SRO

and (c) SCO in the non-faradaic potential region for HER. (d) Calculated double layers capacitances extracted from the linear fitting of the capacitive currents versus CV scan rates for SCRO, SRO and SCO.

Reviewer #3

GENERAL COMMENTS: The authors have well addressed my concerns, now the revised version of this manuscript could be accepted from my side.

Response: We very much appreciate the Reviewer #3's recommendation for publication.
