# Supplementary material for: Trifunctional electrocatalyst with accurate surface reconstruction for zinc-air batteries and water electrolyzers
Source: Nat Commun. 2026 Jun 23;17:7890. doi: 10.1038/s41467-026-74714-5 (PMC13444124; doi:10.1038/s41467-026-74714-5)
Supplement: Supplementary file 4 [file 41467_2026_74714_MOESM3_ESM.docx]

**SCRO-initial structure**

CONTCAR\(6)

1.0

11.6552000046	0.0000000000	0.0000000000

0.0000000000	7.7701001167	0.0000000000

0.0000000000	0.0000000000	7.7701001167

Sr	Ru	O	Co

12	6	36	6

Direct

0.166669995	0.250000000	0.250000000

0.500000000	0.250000000	0.250000000

0.833329976	0.250000000	0.250000000

0.166669995	0.750000000	0.250000000

0.500000000	0.750000000	0.250000000

0.833329976	0.750000000	0.250000000

0.166669995	0.250000000	0.750000000

0.500000000	0.250000000	0.750000000

0.833329976	0.250000000	0.750000000

0.166669995	0.750000000	0.750000000

0.500000000	0.750000000	0.750000000

0.833329976	0.750000000	0.750000000

0.000000000	0.000000000	0.000000000

0.333330005	0.000000000	0.000000000

0.666670024	0.500000000	0.000000000

0.000000000	0.000000000	0.500000000

0.333330005	0.000000000	0.500000000

0.666670024	0.500000000	0.500000000

0.000000000	0.000000000	0.250000000

0.000000000	0.250000000	0.000000000

0.166669995	0.000000000	0.000000000

0.333330005	0.000000000	0.250000000

0.333330005	0.250000000	0.000000000

0.500000000	0.000000000	0.000000000

0.666670024	0.000000000	0.250000000

0.666670024	0.250000000	0.000000000

0.833329976	0.000000000	0.000000000

0.000000000	0.500000000	0.250000000

0.000000000	0.750000000	0.000000000

0.166669995	0.500000000	0.000000000

0.333330005	0.500000000	0.250000000

0.333330005	0.750000000	0.000000000

0.500000000	0.500000000	0.000000000

0.666670024	0.500000000	0.250000000

0.666670024	0.750000000	0.000000000

0.833329976	0.500000000	0.000000000

0.000000000	0.000000000	0.750000000

0.000000000	0.250000000	0.500000000

0.166669995	0.000000000	0.500000000

0.333330005	0.000000000	0.750000000

0.333330005	0.250000000	0.500000000

0.500000000	0.000000000	0.500000000

0.666670024	0.000000000	0.750000000

0.666670024	0.250000000	0.500000000

0.833329976	0.000000000	0.500000000

0.000000000	0.500000000	0.750000000

0.000000000	0.750000000	0.500000000

0.166669995	0.500000000	0.500000000

0.333330005	0.500000000	0.750000000

0.333330005	0.750000000	0.500000000

0.500000000	0.500000000	0.500000000

0.666670024	0.500000000	0.750000000

0.666670024	0.750000000	0.500000000

0.833329976	0.500000000	0.500000000

0.666670024	0.000000000	0.000000000

0.000000000	0.500000000	0.000000000

0.333330005	0.500000000	0.000000000

0.666670024	0.000000000	0.500000000

0.000000000	0.500000000	0.500000000

0.333330005	0.500000000	0.500000000

**SCRO-optimized structure**

CONTCAR\(6)

1.00000000000000

11.6552000045999993	0.0000000000000000	0.0000000000000000

0.0000000000000000	7.7701001167000001	0.0000000000000000

0.0000000000000000	0.0000000000000000	7.7701001167000001

Sr	Ru	O	Co

12	6	36	6

Direct

0.1666747979494758 0.2662970749647187 0.2500000000000000

0.4995425768530595 0.2522391978002432 0.2500000000000000

0.8337840384004735 0.2522362542909775 0.2500000000000000

0.1666747979494758 0.7337029250352813 0.2500000000000000

0.4995425768530595 0.7477608021997568 0.2500000000000000

0.8337840384004735 0.7477637457090225 0.2500000000000000

0.1666747979494758 0.2662970749647187 0.7500000000000000

0.4995425768530595 0.2522391978002432 0.7500000000000000

0.8337840384004735 0.2522362542909775 0.7500000000000000

0.1666747979494758 0.7337029250352813 0.7500000000000000

0.4995425768530595 0.7477608021997568 0.7500000000000000

0.8337840384004735 0.7477637457090225 0.7500000000000000

0.9991039160267263 0.0000000000000000 0.0000000000000000

0.3342262868145909 0.0000000000000000 0.0000000000000000

0.6666708650739750 0.5000000000000000 0.0000000000000000

0.9991039160267263 0.0000000000000000 0.5000000000000000

0.3342262868145909 0.0000000000000000 0.5000000000000000

0.6666708650739750 0.5000000000000000 0.5000000000000000

0.0001277332627083 0.0000000000000000 0.2500000000000000

0.0008451117461803 0.2500381682858759 0.0000000000000000

0.1666687386505004 0.0000000000000000 0.0000000000000000

0.3332038676212790 0.0000000000000000 0.2500000000000000

0.3324879241770731 0.2500385976210495 0.0000000000000000

0.4980339891275278 0.0000000000000000 0.0000000000000000

0.6666676415919213 0.0000000000000000 0.2500000000000000

0.6666647583471530 0.2518319139499994 0.0000000000000000

0.8352980660764473 0.0000000000000000 0.0000000000000000

0.0000028139625528 0.5000000000000000 0.2500000000000000

0.0008451117461803 0.7499618317141241 0.0000000000000000

0.1666679042202119 0.5000000000000000 0.0000000000000000

0.3333318803605678 0.5000000000000000 0.2500000000000000

0.3324879241770731 0.7499614023789505 0.0000000000000000

0.4996144576847072 0.5000000000000000 0.0000000000000000

0.6666637854043529 0.5000000000000000 0.2500000000000000

0.6666647583471530 0.7481680860500006 0.0000000000000000

0.8337156038120881 0.5000000000000000 0.0000000000000000

0.0001277332627083 0.0000000000000000 0.7500000000000000

0.0008451117461803 0.2500381682858759 0.5000000000000000

0.1666687386505004 0.0000000000000000 0.5000000000000000

0.3332038676212790 0.0000000000000000 0.7500000000000000

0.3324879241770731 0.2500385976210495 0.5000000000000000

0.4980339891275278 0.0000000000000000 0.5000000000000000

0.6666676415919213 0.0000000000000000 0.7500000000000000

0.6666647583471530 0.2518319139499994 0.5000000000000000

0.8352980660764473 0.0000000000000000 0.5000000000000000

0.0000028139625528 0.5000000000000000 0.7500000000000000

0.0008451117461803 0.7499618317141241 0.5000000000000000

0.1666679042202119 0.5000000000000000 0.5000000000000000

0.3333318803605678 0.5000000000000000 0.7500000000000000

0.3324879241770731 0.7499614023789505 0.5000000000000000

0.4996144576847072 0.5000000000000000 0.5000000000000000

0.6666637854043529 0.5000000000000000 0.7500000000000000

0.6666647583471530 0.7481680860500006 0.5000000000000000

0.8337156038120881 0.5000000000000000 0.5000000000000000

0.6666678966258814 0.0000000000000000 0.0000000000000000

0.0028321725356193 0.5000000000000000 0.0000000000000000

0.3305040242014954 0.5000000000000000 0.0000000000000000

0.6666678966258814 0.0000000000000000 0.5000000000000000

0.0028321725356193 0.5000000000000000 0.5000000000000000

0.3305040242014954 0.5000000000000000 0.5000000000000000

0.00000000E+00 0.00000000E+00 0.00000000E+00

0.00000000E+00 0.00000000E+00 0.00000000E+00

0.00000000E+00 0.00000000E+00 0.00000000E+00

0.00000000E+00 0.00000000E+00 0.00000000E+00

0.00000000E+00 0.00000000E+00 0.00000000E+00

0.00000000E+00 0.00000000E+00 0.00000000E+00

0.00000000E+00 0.00000000E+00 0.00000000E+00

0.00000000E+00 0.00000000E+00 0.00000000E+00

0.00000000E+00 0.00000000E+00 0.00000000E+00

0.00000000E+00 0.00000000E+00 0.00000000E+00

0.00000000E+00 0.00000000E+00 0.00000000E+00

0.00000000E+00 0.00000000E+00 0.00000000E+00

0.00000000E+00 0.00000000E+00 0.00000000E+00

0.00000000E+00 0.00000000E+00 0.00000000E+00

0.00000000E+00 0.00000000E+00 0.00000000E+00

0.00000000E+00 0.00000000E+00 0.00000000E+00

0.00000000E+00 0.00000000E+00 0.00000000E+00

0.00000000E+00 0.00000000E+00 0.00000000E+00

0.00000000E+00 0.00000000E+00 0.00000000E+00

0.00000000E+00 0.00000000E+00 0.00000000E+00

0.00000000E+00 0.00000000E+00 0.00000000E+00

0.00000000E+00 0.00000000E+00 0.00000000E+00

0.00000000E+00 0.00000000E+00 0.00000000E+00

0.00000000E+00 0.00000000E+00 0.00000000E+00

0.00000000E+00 0.00000000E+00 0.00000000E+00

0.00000000E+00 0.00000000E+00 0.00000000E+00

0.00000000E+00 0.00000000E+00 0.00000000E+00

0.00000000E+00 0.00000000E+00 0.00000000E+00

0.00000000E+00 0.00000000E+00 0.00000000E+00

0.00000000E+00 0.00000000E+00 0.00000000E+00

0.00000000E+00 0.00000000E+00 0.00000000E+00

0.00000000E+00 0.00000000E+00 0.00000000E+00

0.00000000E+00 0.00000000E+00 0.00000000E+00

0.00000000E+00 0.00000000E+00 0.00000000E+00

0.00000000E+00 0.00000000E+00 0.00000000E+00

0.00000000E+00 0.00000000E+00 0.00000000E+00

0.00000000E+00 0.00000000E+00 0.00000000E+00

0.00000000E+00 0.00000000E+00 0.00000000E+00

0.00000000E+00 0.00000000E+00 0.00000000E+00

0.00000000E+00 0.00000000E+00 0.00000000E+00

0.00000000E+00 0.00000000E+00 0.00000000E+00

0.00000000E+00 0.00000000E+00 0.00000000E+00

0.00000000E+00 0.00000000E+00 0.00000000E+00

0.00000000E+00 0.00000000E+00 0.00000000E+00

0.00000000E+00 0.00000000E+00 0.00000000E+00

0.00000000E+00 0.00000000E+00 0.00000000E+00

0.00000000E+00 0.00000000E+00 0.00000000E+00

0.00000000E+00 0.00000000E+00 0.00000000E+00

0.00000000E+00 0.00000000E+00 0.00000000E+00

0.00000000E+00 0.00000000E+00 0.00000000E+00

0.00000000E+00 0.00000000E+00 0.00000000E+00

0.00000000E+00 0.00000000E+00 0.00000000E+00

0.00000000E+00 0.00000000E+00 0.00000000E+00

0.00000000E+00 0.00000000E+00 0.00000000E+00

0.00000000E+00 0.00000000E+00 0.00000000E+00

0.00000000E+00 0.00000000E+00 0.00000000E+00

0.00000000E+00 0.00000000E+00 0.00000000E+00

0.00000000E+00 0.00000000E+00 0.00000000E+00

0.00000000E+00 0.00000000E+00 0.00000000E+00

0.00000000E+00 0.00000000E+00 0.00000000E+00

**SCO-initial structure**

Sr8 Co8 O20

1.0

5.4279079437	0.0000000000	0.0000000000

0.0000000000	5.5387530327	0.0000000000

0.0000000000	0.0000000000	16.2821502686

Sr	Co	O

8	8	20

Direct

0.001144000	0.508885026	0.110279001

0.501143992	0.991114974	0.389721006

0.498856008	0.008885000	0.889720976

0.001144000	0.508885026	0.389721006

0.498856008	0.008885000	0.610279024

0.501143992	0.991114974	0.110279001

0.998856008	0.491115004	0.610279024

0.998856008	0.491115004	0.889720976

0.028774001	0.939378977	0.750000000

0.471226007	0.439379007	0.250000000

0.971225977	0.060621001	0.250000000

0.528774023	0.560621023	0.750000000

0.500000000	0.500000000	0.500000000

0.000000000	0.000000000	0.000000000

0.500000000	0.500000000	0.000000000

0.000000000	0.000000000	0.500000000

0.620837986	0.124389000	0.250000000

0.748842001	0.751257002	0.504471004

0.983605981	0.037317999	0.644999981

0.879162014	0.624388993	0.750000000

0.016394001	0.962682009	0.354999989

0.379162014	0.875611007	0.750000000

0.483606011	0.462682009	0.855000019

0.016394001	0.962682009	0.144999996

0.120838001	0.375611007	0.250000000

0.516394019	0.537317991	0.144999996

0.483606011	0.462682009	0.644999981

0.516394019	0.537317991	0.354999989

0.751157999	0.251257002	0.004471000

0.248842001	0.748742998	0.995528996

0.251157999	0.248742998	0.004471000

0.983605981	0.037317999	0.855000019

0.251157999	0.248742998	0.495528996

0.748842001	0.751257002	0.995528996

0.751157999	0.251257002	0.495528996

0.248842001	0.748742998	0.504471004

**SCO-optimized structure**

Sr8 Co8 O20

1.00000000000000

5.4279079437000002	0.0000000000000000	0.0000000000000000

0.0000000000000000	5.5387530326999999	0.0000000000000000

0.0000000000000000	0.0000000000000000	16.2821502685999988

Sr	Co	O

8	8	20

Direct

0.0008124651905377 0.5090122388618560 0.1103390019445360

0.5008124571905441 0.9909877611381440 0.3896610050554682

0.4991875428094559 0.0090122128618537 0.8896609750554657

0.0008124651905377 0.5090122388618560 0.3896610050554682

0.4991875428094559 0.0090122128618537 0.6103390249445343

0.5008124571905441 0.9909877611381440 0.1103390019445360

0.9991875428094559 0.4909877911381465 0.6103390249445343

0.9991875428094559 0.4909877911381465 0.8896609750554657

0.0320451408083510 0.9352331757943677 0.7500000000000000

0.4679548671916496 0.4352332057943702 0.2500000000000000

0.9679548371916544 0.0647668022056305 0.2500000000000000

0.5320451628083456 0.5647668242056323 0.7500000000000000

0.5000000000000000 0.5000000000000000 0.5000000000000000

0.0000000000000000 0.0000000000000000 0.0000000000000000

0.5000000000000000 0.5000000000000000 0.0000000000000000

0.0000000000000000 0.0000000000000000 0.5000000000000000

0.6250712764327552 0.1297856591378094 0.2500000000000000

0.7487252421585953 0.7510190841060894 0.5037711862662955

0.9876003388127383 0.0303081479104906 0.6444983133778415

0.8749287235672448 0.6297856521378054 0.7500000000000000

0.0123996431872602 0.9696918600895099 0.3555016566221559

0.3749287235672448 0.8702143478621946 0.7500000000000000

0.4876003688127408 0.4696918600895101 0.8555016866221585

0.0123996431872602 0.9696918600895099 0.1444983283778464

0.1250712914327600 0.3702143478621947 0.2500000000000000

0.5123996611872617 0.5303081399104901 0.1444983283778464

0.4876003688127408 0.4696918600895101 0.6444983133778415

0.5123996611872617 0.5303081399104901 0.3555016566221559

0.7512747578414047 0.2510190841060893 0.0037711822662952

0.2487252421585953 0.7489809158939106 0.9962288137337045

0.2512747578414047 0.2489809158939106 0.0037711822662952

0.9876003388127383 0.0303081479104906 0.8555016866221585

0.2512747578414047 0.2489809158939106 0.4962288137337044

0.7487252421585953 0.7510190841060894 0.9962288137337045

0.7512747578414047 0.2510190841060893 0.4962288137337044

0.2487252421585953 0.7489809158939106 0.5037711862662955

0.00000000E+00 0.00000000E+00 0.00000000E+00

0.00000000E+00 0.00000000E+00 0.00000000E+00

0.00000000E+00 0.00000000E+00 0.00000000E+00

0.00000000E+00 0.00000000E+00 0.00000000E+00

0.00000000E+00 0.00000000E+00 0.00000000E+00

0.00000000E+00 0.00000000E+00 0.00000000E+00

0.00000000E+00 0.00000000E+00 0.00000000E+00

0.00000000E+00 0.00000000E+00 0.00000000E+00

0.00000000E+00 0.00000000E+00 0.00000000E+00

0.00000000E+00 0.00000000E+00 0.00000000E+00

0.00000000E+00 0.00000000E+00 0.00000000E+00

0.00000000E+00 0.00000000E+00 0.00000000E+00

0.00000000E+00 0.00000000E+00 0.00000000E+00

0.00000000E+00 0.00000000E+00 0.00000000E+00

0.00000000E+00 0.00000000E+00 0.00000000E+00

0.00000000E+00 0.00000000E+00 0.00000000E+00

0.00000000E+00 0.00000000E+00 0.00000000E+00

0.00000000E+00 0.00000000E+00 0.00000000E+00

0.00000000E+00 0.00000000E+00 0.00000000E+00

0.00000000E+00 0.00000000E+00 0.00000000E+00

0.00000000E+00 0.00000000E+00 0.00000000E+00

0.00000000E+00 0.00000000E+00 0.00000000E+00

0.00000000E+00 0.00000000E+00 0.00000000E+00

0.00000000E+00 0.00000000E+00 0.00000000E+00

0.00000000E+00 0.00000000E+00 0.00000000E+00

0.00000000E+00 0.00000000E+00 0.00000000E+00

0.00000000E+00 0.00000000E+00 0.00000000E+00

0.00000000E+00 0.00000000E+00 0.00000000E+00

0.00000000E+00 0.00000000E+00 0.00000000E+00

0.00000000E+00 0.00000000E+00 0.00000000E+00

0.00000000E+00 0.00000000E+00 0.00000000E+00

0.00000000E+00 0.00000000E+00 0.00000000E+00

0.00000000E+00 0.00000000E+00 0.00000000E+00

0.00000000E+00 0.00000000E+00 0.00000000E+00

0.00000000E+00 0.00000000E+00 0.00000000E+00

0.00000000E+00 0.00000000E+00 0.00000000E+00

**SRO-initial structure**

CONTCAR

1.0

11.0865001678	0.0000000000	0.0000000000

0.0000000000	11.1504001617	0.0000000000

0.0000000000	0.0000000000	7.8765997887

Sr	Ru	O

16	16	48

Direct

0.237350002	0.248070002	0.750000000

0.487349987	0.001930000	0.750000000

0.262650013	0.251929998	0.250000000

0.012650000	0.498070002	0.250000000

0.737349987	0.248070002	0.750000000

0.987349987	0.001930000	0.750000000

0.762650013	0.251929998	0.250000000

0.512650013	0.498070002	0.250000000

0.237350002	0.748070002	0.750000000

0.487349987	0.501929998	0.750000000

0.262650013	0.751929998	0.250000000

0.012650000	0.998070002	0.250000000

0.737349987	0.748070002	0.750000000

0.987349987	0.501929998	0.750000000

0.762650013	0.751929998	0.250000000

0.512650013	0.998070002	0.250000000

0.250000000	0.000000000	0.000000000

0.000000000	0.250000000	0.500000000

0.250000000	0.000000000	0.500000000

0.000000000	0.250000000	0.000000000

0.750000000	0.000000000	0.000000000

0.500000000	0.250000000	0.500000000

0.750000000	0.000000000	0.500000000

0.500000000	0.250000000	0.000000000

0.250000000	0.500000000	0.000000000

0.000000000	0.750000000	0.500000000

0.250000000	0.500000000	0.500000000

0.000000000	0.750000000	0.000000000

0.750000000	0.500000000	0.000000000

0.500000000	0.750000000	0.500000000

0.750000000	0.500000000	0.500000000

0.500000000	0.750000000	0.000000000

0.109080002	0.108989999	0.968379974

0.359079987	0.141010001	0.531620026

0.390920013	0.391009986	0.468380004

0.140919998	0.358990014	0.031620000

0.390920013	0.391009986	0.031620000

0.140919998	0.358990014	0.468380004

0.109080002	0.108989999	0.531620026

0.359079987	0.141010001	0.968379974

0.002440000	0.280339986	0.750000000

0.252429992	0.469660014	0.750000000

0.497570008	0.219659999	0.250000000

0.247559994	0.030339999	0.250000000

0.609080017	0.108989999	0.968379974

0.859080017	0.141010001	0.531620026

0.890919983	0.391009986	0.468380004

0.640919983	0.358990014	0.031620000

0.890919983	0.391009986	0.031620000

0.640919983	0.358990014	0.468380004

0.609080017	0.108989999	0.531620026

0.859080017	0.141010001	0.968379974

0.502430022	0.280339986	0.750000000

0.752430022	0.469660014	0.750000000

0.997569978	0.219659999	0.250000000

0.747560024	0.030339999	0.250000000

0.109080002	0.608990014	0.968379974

0.359079987	0.641009986	0.531620026

0.390920013	0.891009986	0.468380004

0.140919998	0.858990014	0.031620000

0.390920013	0.891009986	0.031620000

0.140919998	0.858990014	0.468380004

0.109080002	0.608990014	0.531620026

0.359079987	0.641009986	0.968379974

0.002440000	0.780340016	0.750000000

0.252429992	0.969659984	0.750000000

0.497570008	0.719659984	0.250000000

0.247559994	0.530340016	0.250000000

0.609080017	0.608990014	0.968379974

0.859080017	0.641009986	0.531620026

0.890919983	0.891009986	0.468380004

0.640919983	0.858990014	0.031620000

0.890919983	0.891009986	0.031620000

0.640919983	0.858990014	0.468380004

0.609080017	0.608990014	0.531620026

0.859080017	0.641009986	0.968379974

0.502430022	0.780340016	0.750000000

0.752430022	0.969659984	0.750000000

0.997569978	0.719659984	0.250000000

0.747560024	0.530340016	0.250000000

**SRO-optimized structure**

CONTCAR

1.00000000000000

11.0865001678000006	0.0000000000000000	0.0000000000000000

0.0000000000000000	11.1504001617000004	0.0000000000000000

0.0000000000000000	0.0000000000000000	7.8765997887000001

Sr	Ru	O

16	16	48

Direct

0.2373500019999994 0.2480700019999986 0.7500000000000000

0.4873499870000018 0.0019300000000015 0.7500000000000000

0.2626500129999982 0.2519299980000014 0.2500000000000000

0.0126500000000007 0.4980700019999986 0.2500000000000000

0.7373499870000018 0.2480700019999986 0.7500000000000000

0.9873499870000018 0.0019300000000015 0.7500000000000000

0.7626500129999982 0.2519299980000014 0.2500000000000000

0.5126500129999982 0.4980700019999986 0.2500000000000000

0.2373500019999994 0.7480700019999986 0.7500000000000000

0.4873499870000018 0.5019299980000014 0.7500000000000000

0.2626500129999982 0.7519299980000014 0.2500000000000000

0.0126500000000007 0.9980700019999986 0.2500000000000000

0.7373499870000018 0.7480700019999986 0.7500000000000000

0.9873499870000018 0.5019299980000014 0.7500000000000000

0.7626500129999982 0.7519299980000014 0.2500000000000000

0.5126500129999982 0.9980700019999986 0.2500000000000000

0.2500000000000000 0.0000000000000000 0.0000000000000000

0.0000000000000000 0.2500000000000000 0.5000000000000000

0.2500000000000000 0.0000000000000000 0.5000000000000000

0.0000000000000000 0.2500000000000000 0.0000000000000000

0.7500000000000000 0.0000000000000000 0.0000000000000000

0.5000000000000000 0.2500000000000000 0.5000000000000000

0.7500000000000000 0.0000000000000000 0.5000000000000000

0.5000000000000000 0.2500000000000000 0.0000000000000000

0.2500000000000000 0.5000000000000000 0.0000000000000000

0.0000000000000000 0.7500000000000000 0.5000000000000000

0.2500000000000000 0.5000000000000000 0.5000000000000000

0.0000000000000000 0.7500000000000000 0.0000000000000000

0.7500000000000000 0.5000000000000000 0.0000000000000000

0.5000000000000000 0.7500000000000000 0.5000000000000000

0.7500000000000000 0.5000000000000000 0.5000000000000000

0.5000000000000000 0.7500000000000000 0.0000000000000000

0.1090800019999989 0.1089899990000021 0.9683799740000012

0.3590799870000012 0.1410100009999979 0.5316200259999988

0.3909200129999988 0.3910099860000003 0.4683800039999966

0.1409199980000011 0.3589900139999997 0.0316199999999967

0.3909200129999988 0.3910099860000003 0.0316199999999967

0.1409199980000011 0.3589900139999997 0.4683800039999966

0.1090800019999989 0.1089899990000021 0.5316200259999988

0.3590799870000012 0.1410100009999979 0.9683799740000012

0.0024400000000000 0.2803399860000013 0.7500000000000000

0.2524299920000033 0.4696600139999987 0.7500000000000000

0.4975700079999967 0.2196599990000010 0.2500000000000000

0.2475599939999995 0.0303399989999988 0.2500000000000000

0.6090800169999966 0.1089899990000021 0.9683799740000012

0.8590800169999966 0.1410100009999979 0.5316200259999988

0.8909199830000034 0.3910099860000003 0.4683800039999966

0.6409199830000034 0.3589900139999997 0.0316199999999967

0.8909199830000034 0.3910099860000003 0.0316199999999967

0.6409199830000034 0.3589900139999997 0.4683800039999966

0.6090800169999966 0.1089899990000021 0.5316200259999988

0.8590800169999966 0.1410100009999979 0.9683799740000012

0.5024300219999986 0.2803399860000013 0.7500000000000000

0.7524300219999986 0.4696600139999987 0.7500000000000000

0.9975699780000014 0.2196599990000010 0.2500000000000000

0.7475600240000020 0.0303399989999988 0.2500000000000000

0.1090800019999989 0.6089900139999997 0.9683799740000012

0.3590799870000012 0.6410099860000003 0.5316200259999988

0.3909200129999988 0.8910099860000003 0.4683800039999966

0.1409199980000011 0.8589900139999997 0.0316199999999967

0.3909200129999988 0.8910099860000003 0.0316199999999967

0.1409199980000011 0.8589900139999997 0.4683800039999966

0.1090800019999989 0.6089900139999997 0.5316200259999988

0.3590799870000012 0.6410099860000003 0.9683799740000012

0.0024400000000000 0.7803400159999967 0.7500000000000000

0.2524299920000033 0.9696599840000033 0.7500000000000000

0.4975700079999967 0.7196599840000033 0.2500000000000000

0.2475599939999995 0.5303400159999967 0.2500000000000000

0.6090800169999966 0.6089900139999997 0.9683799740000012

0.8590800169999966 0.6410099860000003 0.5316200259999988

0.8909199830000034 0.8910099860000003 0.4683800039999966

0.6409199830000034 0.8589900139999997 0.0316199999999967

0.8909199830000034 0.8910099860000003 0.0316199999999967

0.6409199830000034 0.8589900139999997 0.4683800039999966

0.6090800169999966 0.6089900139999997 0.5316200259999988

0.8590800169999966 0.6410099860000003 0.9683799740000012

0.5024300219999986 0.7803400159999967 0.7500000000000000

0.7524300219999986 0.9696599840000033 0.7500000000000000

0.9975699780000014 0.7196599840000033 0.2500000000000000

0.7475600240000020 0.5303400159999967 0.2500000000000000

0.00000000E+00 0.00000000E+00 0.00000000E+00

0.00000000E+00 0.00000000E+00 0.00000000E+00

0.00000000E+00 0.00000000E+00 0.00000000E+00

0.00000000E+00 0.00000000E+00 0.00000000E+00

0.00000000E+00 0.00000000E+00 0.00000000E+00

0.00000000E+00 0.00000000E+00 0.00000000E+00

0.00000000E+00 0.00000000E+00 0.00000000E+00

0.00000000E+00 0.00000000E+00 0.00000000E+00

0.00000000E+00 0.00000000E+00 0.00000000E+00

0.00000000E+00 0.00000000E+00 0.00000000E+00

0.00000000E+00 0.00000000E+00 0.00000000E+00

0.00000000E+00 0.00000000E+00 0.00000000E+00

0.00000000E+00 0.00000000E+00 0.00000000E+00

0.00000000E+00 0.00000000E+00 0.00000000E+00

0.00000000E+00 0.00000000E+00 0.00000000E+00

0.00000000E+00 0.00000000E+00 0.00000000E+00

0.00000000E+00 0.00000000E+00 0.00000000E+00

0.00000000E+00 0.00000000E+00 0.00000000E+00

0.00000000E+00 0.00000000E+00 0.00000000E+00

0.00000000E+00 0.00000000E+00 0.00000000E+00

0.00000000E+00 0.00000000E+00 0.00000000E+00

0.00000000E+00 0.00000000E+00 0.00000000E+00

0.00000000E+00 0.00000000E+00 0.00000000E+00

0.00000000E+00 0.00000000E+00 0.00000000E+00

0.00000000E+00 0.00000000E+00 0.00000000E+00

0.00000000E+00 0.00000000E+00 0.00000000E+00

0.00000000E+00 0.00000000E+00 0.00000000E+00

0.00000000E+00 0.00000000E+00 0.00000000E+00

0.00000000E+00 0.00000000E+00 0.00000000E+00

0.00000000E+00 0.00000000E+00 0.00000000E+00

0.00000000E+00 0.00000000E+00 0.00000000E+00

0.00000000E+00 0.00000000E+00 0.00000000E+00

0.00000000E+00 0.00000000E+00 0.00000000E+00

0.00000000E+00 0.00000000E+00 0.00000000E+00

0.00000000E+00 0.00000000E+00 0.00000000E+00

0.00000000E+00 0.00000000E+00 0.00000000E+00

0.00000000E+00 0.00000000E+00 0.00000000E+00

0.00000000E+00 0.00000000E+00 0.00000000E+00

0.00000000E+00 0.00000000E+00 0.00000000E+00

0.00000000E+00 0.00000000E+00 0.00000000E+00

0.00000000E+00 0.00000000E+00 0.00000000E+00

0.00000000E+00 0.00000000E+00 0.00000000E+00

0.00000000E+00 0.00000000E+00 0.00000000E+00

0.00000000E+00 0.00000000E+00 0.00000000E+00

0.00000000E+00 0.00000000E+00 0.00000000E+00

0.00000000E+00 0.00000000E+00 0.00000000E+00

0.00000000E+00 0.00000000E+00 0.00000000E+00

0.00000000E+00 0.00000000E+00 0.00000000E+00

0.00000000E+00 0.00000000E+00 0.00000000E+00

0.00000000E+00 0.00000000E+00 0.00000000E+00

0.00000000E+00 0.00000000E+00 0.00000000E+00

0.00000000E+00 0.00000000E+00 0.00000000E+00

0.00000000E+00 0.00000000E+00 0.00000000E+00

0.00000000E+00 0.00000000E+00 0.00000000E+00

0.00000000E+00 0.00000000E+00 0.00000000E+00

0.00000000E+00 0.00000000E+00 0.00000000E+00

0.00000000E+00 0.00000000E+00 0.00000000E+00

0.00000000E+00 0.00000000E+00 0.00000000E+00

0.00000000E+00 0.00000000E+00 0.00000000E+00

0.00000000E+00 0.00000000E+00 0.00000000E+00

0.00000000E+00 0.00000000E+00 0.00000000E+00

0.00000000E+00 0.00000000E+00 0.00000000E+00

0.00000000E+00 0.00000000E+00 0.00000000E+00

0.00000000E+00 0.00000000E+00 0.00000000E+00

0.00000000E+00 0.00000000E+00 0.00000000E+00

0.00000000E+00 0.00000000E+00 0.00000000E+00

0.00000000E+00 0.00000000E+00 0.00000000E+00

0.00000000E+00 0.00000000E+00 0.00000000E+00

0.00000000E+00 0.00000000E+00 0.00000000E+00

0.00000000E+00 0.00000000E+00 0.00000000E+00

0.00000000E+00 0.00000000E+00 0.00000000E+00

0.00000000E+00 0.00000000E+00 0.00000000E+00

0.00000000E+00 0.00000000E+00 0.00000000E+00

0.00000000E+00 0.00000000E+00 0.00000000E+00

0.00000000E+00 0.00000000E+00 0.00000000E+00

0.00000000E+00 0.00000000E+00 0.00000000E+00

0.00000000E+00 0.00000000E+00 0.00000000E+00

0.00000000E+00 0.00000000E+00 0.00000000E+00

0.00000000E+00 0.00000000E+00 0.00000000E+00

0.00000000E+00 0.00000000E+00 0.00000000E+00
